# Ascending neurons convey behavioral state to integrative sensory and action selection brain regions

**DOI:** 10.1038/s41593-023-01281-z

**Published:** 2023-03-23

**Authors:** Chin-Lin Chen, Florian Aymanns, Ryo Minegishi, Victor D. V. Matsuda, Nicolas Talabot, Semih Günel, Barry J. Dickson, Pavan Ramdya

**Affiliations:** 1 Neuroengineering Laboratory, Brain Mind Institute & Interfaculty Institute of Bioengineering, EPFL, Lausanne, Switzerland; 2 Janelia Research Campus, Howard Hughes Medical Institute, Ashburn, VA USA; 3 Computer Vision Laboratory, EPFL, Lausanne, Switzerland

**Keywords:** Neural circuits, High-throughput screening, Spinal cord, Software

## Abstract

Knowing one’s own behavioral state has long been theorized as critical for contextualizing dynamic sensory cues and identifying appropriate future behaviors. Ascending neurons (ANs) in the motor system that project to the brain are well positioned to provide such behavioral state signals. However, what ANs encode and where they convey these signals remains largely unknown. Here, through large-scale functional imaging in behaving animals and morphological quantification, we report the behavioral encoding and brain targeting of hundreds of genetically identifiable ANs in the adult fly, *Drosophila melanogaster*. We reveal that ANs encode behavioral states, specifically conveying self-motion to the anterior ventrolateral protocerebrum, an integrative sensory hub, as well as discrete actions to the gnathal ganglia, a locus for action selection. Additionally, AN projection patterns within the motor system are predictive of their encoding. Thus, ascending populations are well poised to inform distinct brain hubs of self-motion and ongoing behaviors and may provide an important substrate for computations that are required for adaptive behavior.

## Abstract (web summary)

Knowing one’s own behavioral state is important to contextualize sensory cues and identify appropriate future actions. Here the authors show how neurons ascending from the fly motor system convey behavioral state signals to specific brain regions.

## Main

To generate adaptive behaviors, animals^1^ and robots^2^ must not only sense their environment but also be aware of their own ongoing behavioral state. Knowing if one is at rest or in motion permits the accurate interpretation of whether sensory cues, such as visual motion during feature tracking or odor intensity fluctuations during plume following, result from exafference (the movement of objects in the world) or reafference (self-motion of the body through space with respect to stationary objects)^1^. Additionally, being aware of one’s current posture enables the selection of future behaviors that are not destabilizing or physically impossible.

In line with these theoretical predictions, neural representations of ongoing behavioral states have been widely observed across the brains of mice^3–5^ and flies (*Drosophila melanogaster*)^6–9^. Furthermore, studies in *Drosophila* have supported roles for behavioral state signals in sensory contextualization (for example, flight^6^ and walking^7^ modulate neurons in the visual system^8,10^) and action selection (for example, an animal’s walking speed regulates its decision to run or freeze in response to a fear-inducing stimulus^11^). Locomotion has also been shown to play an important role in regulating complex behaviors, including song patterning^12^ and reinforcement learning^13^.

Despite these advances, the cellular origins of behavioral state signals in the brain remain largely unknown. They may arise from efference copies of signals generated by descending neurons (DNs) in the brain that drive downstream motor systems^1^. However, because the brain’s descending commands are further sculpted by musculoskeletal interactions with the environment, a more categorically and temporally precise readout of behavioral states might be obtained from ascending neurons (ANs) in the motor system that process proprioceptive and tactile signals and project to the brain. Although these behavioral signals might be conveyed by a subset of primary mechanosensory neurons in the limbs^14^, they are more likely to be computed and conveyed by second-order and higher-order ANs residing in the spinal cord of vertebrates^15–18^ or in the insect ventral nerve cord (VNC)^19^. In *Drosophila*, ANs process limb proprioceptive and tactile signals^14,20,21^, possibly to generate a readout of ongoing movements and behavioral states.

To date, only a few genetically identifiable AN cell types have been studied in behaving animals. These are primarily in the fly, *D. melanogaster*, an organism that has a relatively small number of neurons that can be genetically targeted for repeated investigation. Microscopy recordings of AN terminals in the brain have shown that Lco2N1 and Les2N1D ANs are active during walking^22^ and that LAL-PS-ANs convey walking signals to the visual system^23^. Additionally, artificial activation of pairs of PER_in_ ANs^24^ or moonwalker ANs^25^ regulates action selection and behavioral persistence, respectively.

**Fig. 1:**
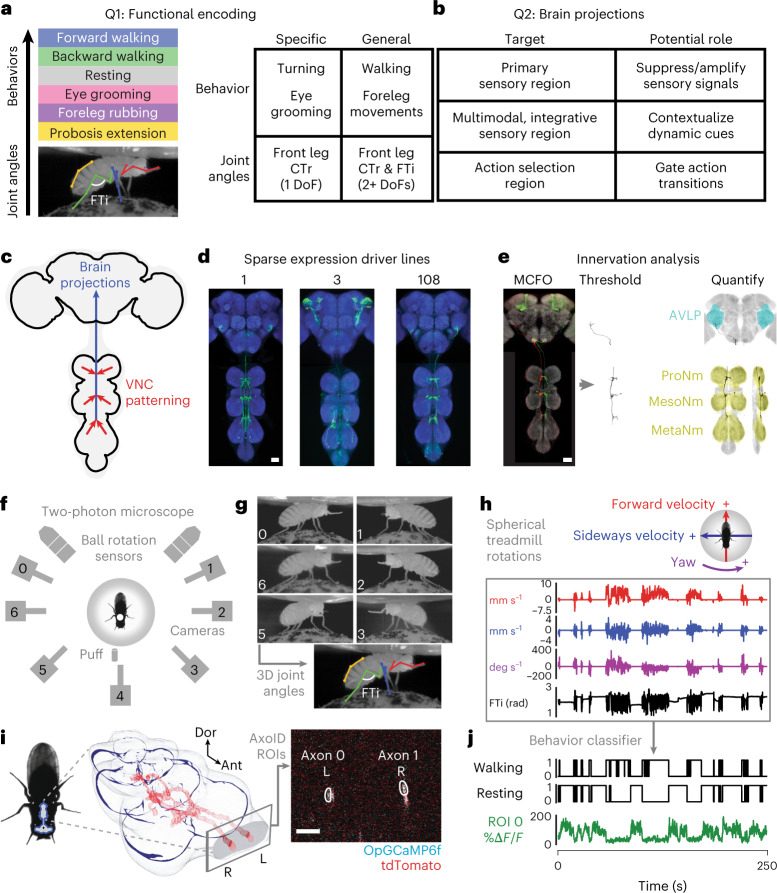
Large-scale functional and morphological screen of AN movement encoding and nervous system targeting. **a**–**c**, Schematics and tables of the main questions addressed. **a**, To what extent do ANs encode longer time-scale behavioral states and limb movements? This encoding may be either specific (for example, encoding specific kinematics of a behavior or one joint degree of freedom) or general (for example, encoding a behavioral state irrespective of specific limb kinematics or encoding multiple joint degrees of freedom). Here, we highlight the CTr and FTi joints. **b**, Where in the brain do ANs convey behavioral states? ANs might target the brain’s (1) primary sensory regions (for example, optic lobe or antennal lobe) for sensory gain control; (2) multimodal and integrative sensory regions (for example, AVLP or mushroom body) to contextualize dynamic, time-varying sensory cues; and (3) action selection centers (for example, GNG or central complex) to gate behavioral transitions. Individual ANs may project broadly to multiple brain regions or narrowly to one region. **c**, To what extent is an AN’s patterning within the VNC predictive of its brain targeting and encoding? **d**, We screened 108 sparsely expressing driver lines. The projection patterns of the lines with active ANs and high SNR (157 ANs) were examined in the brain and VNC. Scale bar, 40 μm. **e**, These were quantified by tracing single-cell MCFO confocal images. We highlight projections of one spGal4 to the brain’s AVLP and the VNC’s prothoracic (‘ProNm’), mesothoracic (‘MesoNm’) and metathoracic neuromeres (‘MetaNm’). Scale bar is as in **d**. **f**, Overhead schematic of the behavior measurement system used during two-photon microscopy. A camera array captures six views of the animal. Two optic flow sensors measure ball rotations. A puff of CO_2_ (or air) is used to elicit behavior from sedentary animals. **g**, 2D poses are estimated for six camera views using DeepFly3D. These data are triangulated to quantify 3D poses and joint angles for six legs and the abdomen (color-coded). The FTi joint angle is indicated (white). **h**, Two optic flow sensors measure rotations of the spherical treadmill as a proxy for forward (red), sideways (blue) and yaw (purple) walking velocities. Positive directions of rotation (‘+’) are indicated. **i**, Left: a volumetric representation of the VNC, including a reconstruction of ANs targeted by the SS27485-spGal4 driver line (red). Indicated are the dorsal-ventral (‘Dor’) and anterior-posterior (‘Ant’) axes as well as the fly’s left (L) and right (R) sides. **i**, Right: sample two-photon cross-section image of the thoracic neck connective showing ANs that express OpGCaMP6f (cyan) and tdTomato (red). AxoID is used to semi-automatically identify two axonal ROIs (white) on the left (L) and right (R) sides of the connective. **j**, Spherical treadmill rotations and joint angles are used to classify behaviors. Binary classifications are then compared with simultaneously recorded neural activity for 250-s trials of spontaneous and puff-elicited behaviors. Shown is an activity trace from ROI 0 (green) in **i**. DoF, degree of freedom.

These first insights motivate a more comprehensive, quantitative analysis of large AN populations to investigate three questions. First, what information do ANs convey to the brain (Fig. 1a)? They might encode posture or movements of the joints or limbs as well as longer time-scale behavioral states, such as whether an animal is walking or grooming. Second, where do ANs convey this information to in the brain (Fig. 1b)? They might project widely across brain regions or narrowly target circuit hubs mediating specific computations. Third, what can an AN’s patterning within the VNC tell us about how it derives its encoding (Fig. 1c, red)? Answering these questions would open the door to a cellular-level understanding of how neurons encode behavioral states by integrating proprioceptive, tactile and other sensory feedback signals. It would also enable the study of how behavioral state signals are used by brain circuits to contextualize multimodal cues and to select appropriate future behaviors.

Here, we address these questions by screening a library of split-Gal4 *Drosophila* driver lines (R.M. and B.J.D., unpublished). These, along with the published MAN-spGal4 (ref. ^25^) and 12 sparsely expressing Gal4 driver lines^26^, allowed us to gain repeated genetic access to 247 regions of interest (ROIs) that may each include one or more ANs (Fig. 1d and Supplementary Table 1). Using these driver lines and a MultiColor FlpOut (MCFO) approach^27^, we quantified the projections of ANs within the brain and VNC (Fig. 1e). Additionally, we screened the encoding of these ANs by performing functional recordings of neural activity within the VNC of tethered, behaving flies^28^. To overcome noise and movement-related deformations in imaging data, we developed ‘AxoID’, a deep-learning-based software that semi-automatically identifies and tracks axonal ROIs (Methods). Finally, we precisely quantified joint angles and limb kinematics using a multi-camera array that recorded behaviors during two-photon imaging. We processed these videos using DeepFly3D, a deep-learning-based three-dimensional (3D) pose estimation software^29^. By combining these 3D joint positions with recorded spherical treadmill rotations (a proxy for locomotor velocities^30^), we could classify behavioral time series to study the relationship between ongoing behavioral states and neural activity using linear models.

These analyses uncovered that, as a population, ANs do not project broadly across the brain but principally target two regions: (1) the anterior ventrolateral protocerebrum (AVLP), a site that may mediate higher-order multimodal convergence—vision^31^, olfaction^32^, audition^33–35^ and taste^36^—and (2) the gnathal ganglia (GNG), a region that receives heavy innervation from descending premotor neurons and has been implicated in action selection^24,37,38^. We found that ANs encode behavioral states but most predominantly encode walking. These distinct behavioral states are systematically conveyed to different brain targets. The AVLP is informed of self-motion states, such as resting and walking, and the presence of gust-like stimuli, possibly to contextualize sensory cues. By contrast, the GNG receives signals about specific behavioral states—turning, eye grooming and proboscis extension—likely to guide action selection.

To understand the relationship between AN behavioral state encoding and brain projection patterns, we then performed a more in-depth investigation of seven AN classes. We observed a correspondence between the morphology of ANs in the VNC and their behavioral state encoding: ANs with neurites targeting all three VNC neuromeres (T1–T3) encode global locomotor states (for example, resting and walking), whereas those projecting only to the T1 prothoracic neuromere encode foreleg-dependent behavioral states (for example, eye grooming). Notably, we also observed AN axons within the VNC. This suggests that ANs are not simply passive relays of behavioral state signals to the brain but may also help to orchestrate movements and/or compute state encoding. This latter possibility is illustrated by a class of proboscis extension ANs (‘PE-ANs’) that appear to encode the number of PEs generated over tens of seconds, possibly through recurrent interconnectivity within the VNC. Taken together, these data provide a first large-scale view of ascending signals to the brain, opening the door for a cellular-level understanding of how behavioral states are computed and how ascending motor signals allow the brain to contextualize sensory signals and select appropriate future behaviors.

## Results

### A screen of AN encoding and projection patterns

**Extended Data Fig. 1:**
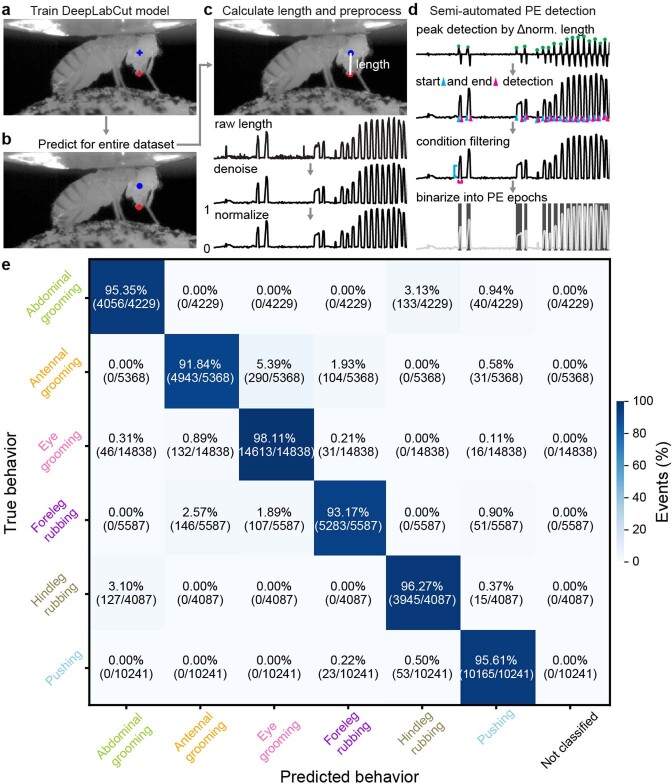
Semi-automated tracking of proboscis extensions, and the accuracy of the behavioral classifier. We detected proboscis extensions using side-view camera images. **(a)** First, we trained a deep neural network model with manual annotations of landmarks on the ventral eye (blue cross) and distal proboscis tip (red cross). **(b)** Then we applied the trained model to estimate these locations throughout the entire dataset. **(c)** Proboscis extension length was calculated as the denoised and normalized distance between landmarks. **(d)** Using these data, we per- formed semi-automated detection of PE epochs by first identifying peaks from normalized proboscis extension lengths. Then we detected the start (cyan triangle) and end (magenta triangle) of these events. We removed false-positive detections by thresholding the amplitude (cyan line) and duration (magenta line) of events. Finally, we generated a binary trace of PE epochs (shaded regions). **(e)** A confusion matrix quantifies the accuracy of behavioral state classification using 10-fold, stratified cross-validation of a histogram gradient boosting classifier. Walking and resting are not included in this evaluation because they are predicted using spherical treadmill rotation data. The percentage of events in each category (‘predicted’ behavior versus ground-truth, manually-labelled ‘true’ behavior) is color-coded.

We performed a screen of 108 driver lines that each express fluorescent reporters in a small number of ANs (Fig. 1d). This allowed us to address to what extent ANs encode particular behavioral states and, to some degree given the limited temporal resolution of calcium imaging, limb movements. To achieve precise behavioral classification, we quantified limb movements by recording each fly using six synchronized cameras (a seventh camera was used to position the fly on the ball) (Fig. 1f). We processed these videos using DeepFly3D (ref. ^29^), a markerless 3D pose estimation software that outputs joint positions and angles (Fig. 1g). We also measured spherical treadmill rotations using two optic flow sensors^30^ and converted these into three fly-centric velocities—forward (millimeters per second), sideways (millimeters per second) and yaw (degrees per second) (Fig. 1h)—that correspond to forward/backward walking, side-slip and turning, respectively. A separate DeepLabCut^39^ deep neural network was used to track PEs from one camera view (Extended Data Fig. 1a–d). We studied spontaneously generated behaviors but also used a puff of CO_2_ to elicit behaviors from sedentary animals.

**Extended Data Fig. 2:**
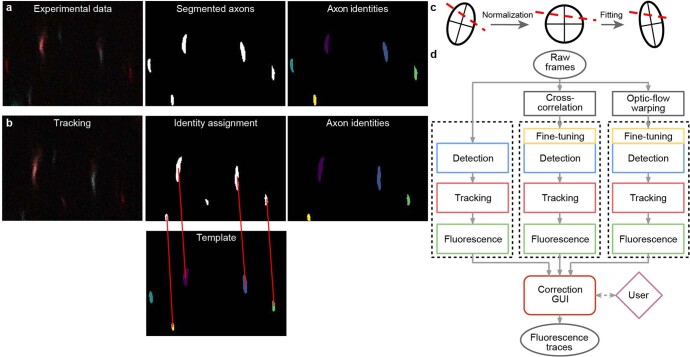
AxoID, a deep learning-based algorithm that detects and tracks axon cross-sections in two-photon microscopy images. **(a)** Pipeline overview: a single image frame (left) is segmented (middle) during the detection stage with potential axons shown (white). Tracking identities (right) are then assigned to these ROIs. **(b)** To track ROIs across time, ROIs in a tracker template (bottom-middle) are matched (red lines) to ROIs in the current segmented frame (top-middle). An undetected axon in the tracker template (cyan) is left unmatched. **(c)** ROI separation is performed for fused axons. An ellipse is first fit to the ROI’s contour and a line is fit to the separation (dashed red line). For normalization, the ellipse is transformed into an axis-aligned circle and the linear separation is transformed accordingly. For another frame, a transformation of the circle into a newly fit ellipse is computed and applied to the line. The ellipse’s main axes are shown for clarity. **(d)** The AxoID workflow. Raw experimental data is first registered via cross-correlation and optic flow warping. Then, raw and registered data are separately processed by the fluorescence extraction pipeline (dashed rectangles). Finally, a GUI is used to select and correct the results.

Synchronized with movement quantification, we recorded the activity of ANs by performing two-photon imaging of the cervical connective within the thoracic VNC^28^. The VNC houses motor circuits that are functionally equivalent to those in the vertebrate spinal cord (Fig. 1i, left). Neural activity was measured using the proxy of changes in the fluorescence intensity of a genetically-encoded calcium indicator, OpGCaMP6f, expressed in a small number of ANs. Simultaneously, we recorded tdTomato fluorescence as an anatomical fiduciary. Imaging coronal (*x*–*z*) sections of the cervical connective kept AN axons within the imaging field of view despite behaviorally induced motion artifacts that would disrupt conventional horizontal (*x*–*y*) section imaging^28^. Sparse spGal4 and Gal4 fluorescent reporter expression facilitated axonal ROI detection. To semi-automatically segment and track AN ROIs across thousands of imaging frames, we developed and used AxoID, a deep-network-based software (Fig. 1i, right, and Extended Data Fig. 2). AxoID also facilitated ROI detection despite large movement-related ROI translations and deformations as well as, for some driver lines, relatively low transgene expression levels and a suboptimal imaging signal-to-noise ratio (SNR).

To relate AN neural activity with ongoing limb movements, we trained classifiers using 3D joint angles and spherical treadmill rotational velocities. This allowed us to accurately and automatically detect nine behaviors: forward and backward walking, spherical treadmill pushing, resting, eye and antennal grooming, foreleg and hindleg rubbing and abdominal grooming (Fig. 1j). This classification was highly accurate (Extended Data Fig. 1e). Additionally, we classified non-orthogonal, co-occurring behaviors, such as PEs, and recorded the timing of CO_2_ puff stimuli (Supplementary Video 1).

Our final dataset comprised 247 ANs/ROIs targeted using 70 sparsely labeled driver lines (more than 32 h of data). We note that an individual ROI may consist of intermingled fibers from several ANs of the same class. These data included (1) anatomical projection patterns and temporally synchronized (2) neural activity, (3) joint angles and (4) spherical treadmill rotations. Here, we focus on the results for 157 of the most active ROIs taken from 50 driver lines (more than 23 h of data) (Supplementary Video 2). The remainder were excluded owing to redundancy with other driver lines, an absence of neural activity or a low SNR (as determined by smFP confocal imaging or two-photon imaging of tdTomato and OpGCaMP6f). Representative data from each of these selected driver lines illustrate the richness of our dataset (Supplementary Videos 3–52; see data repository).

### Behavioral encoding of ANs

Previous studies of AN encoding^22–24^ did not quantify behaviors at high enough resolution or study more than a few ANs. Therefore, it remains unclear to what extent as a population ANs encode specific behavioral states, such as walking, resting and grooming (Fig. 1a). With the data from our large-scale functional screen, we performed a linear regression analysis to quantify the degree to which epochs of behaviors could explain the time course of AN activity. We also examined the encoding of leg movements and joint angles to the extent that the relatively slow temporal resolution of calcium imaging would permit.

Specifically, we quantified the unique explained variance (UEV, or Δ*R*^2^) for each behavioral or movement regressor via cross-validation by subtracting a reduced model *R*^2^ from a full regression model *R*^2^. In the reduced model, the regressor of interest was shuffled while keeping the other regressors intact (Methods). To compensate for the temporal mismatch between fast leg movements and slower calcium signal decay dynamics, every joint angle and behavioral state regressor was convolved with a calcium indicator decay kernel chosen to maximize the explained variance in neural activity, with the aim of reducing the occurrence of false negatives.

**Fig. 2:**
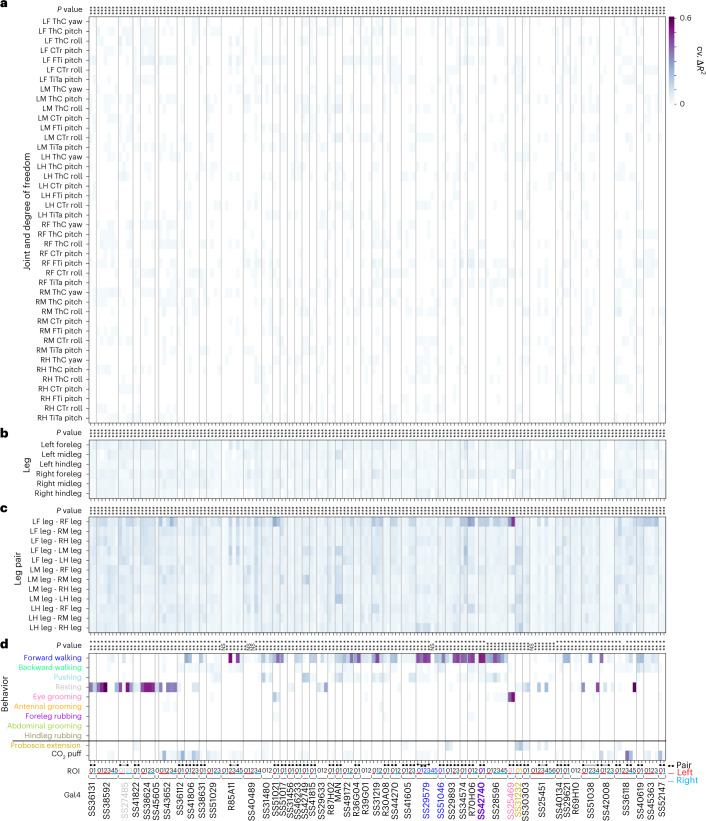
ANs encode behavioral states. Proportion of variance in AN activity that is uniquely explained by regressors (cross-validated Δ*R*^2^) based on joint movements (**a**) (abbreviations refer to the left (L), right (R), front (F), middle (M) or hind (H) legs as well as joints at the thorax (Th), coxa (C), trochanter (Tr), femur (F), tibia (Ti) and tarsus (Ta)). Movements of individual legs (**b**), movements of pairs of legs (**c**) and behaviors (**d**). Regression analyses were performed for 157 ANs recorded from 50 driver lines. Lines selected for more in-depth analysis are color-coded by the behavioral class best explaining their neural activity: SS27485 (resting), SS36112 (puff responses), SS29579 (walking), SS51046 (turning), SS42740 (foreleg movements), SS25469 (eye grooming) and SS31232 (PEs). Non-orthogonal regressors (PE and CO_2_ puffs) are separated from the others. *P* values report the one-tailed *F*-statistic of overall significance of the complete regression model with none of the regressors shuffled without an adjustment for multiple comparisons (*P < 0.05, **P < 0.01 and ***P < 0.001). Indicated are putative pairs of neurons (black ball-and-stick labels) and ROIs that are on the left (red) or right (cyan) side of the cervical connective. Source data

**Extended Data Fig. 3:**
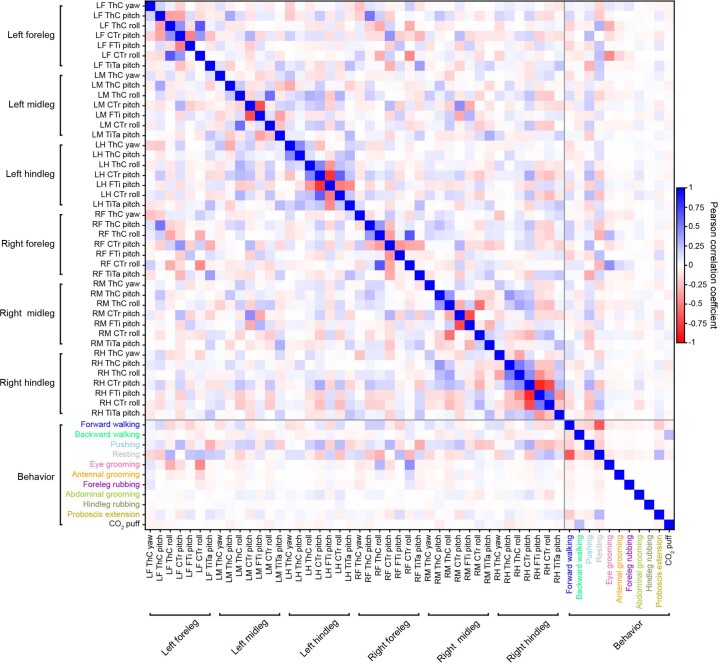
Correlations among and between joint angles and behavioral states. Pearson correlation coefficients (color-coded) for joint angles, behavioral states, proboscis extensions, and puffs. Source data

**Extended Data Fig. 4:**
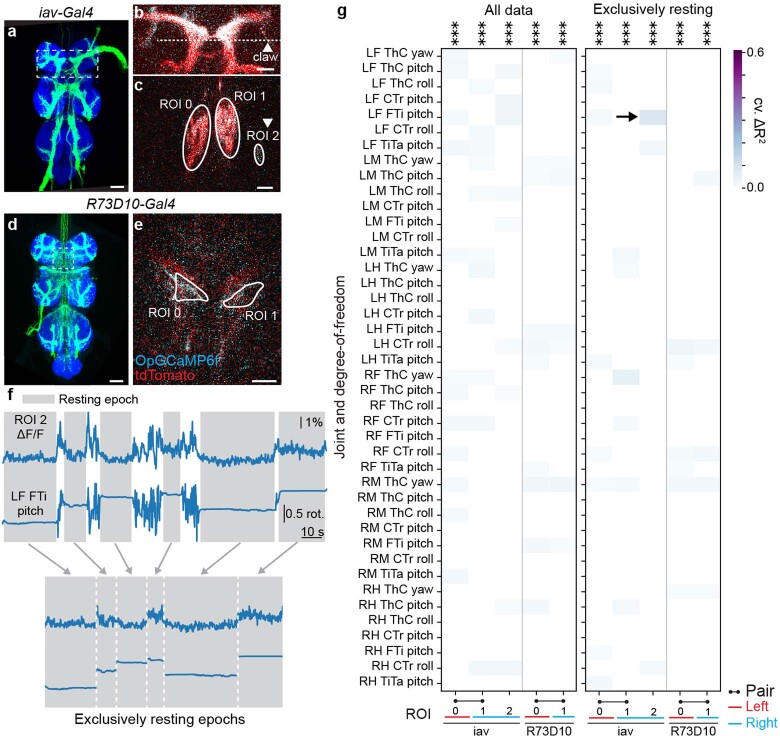
Proprioceptor driver lines and computational pipeline for extracting joint angle encoding in limb proprioceptors. **(a,d)** Standard deviation projection of confocal images showing expression in the leg proprioceptor sensory neuron driver lines **(a)** iav-Gal4 and **(d)** R73D10-Gal4. Indicated are two-photon coronal section imaging regions-of-interest (white dashed boxes). Scale bars are 40 *μ*m. **(b)** Two-photon image of proprioceptor afferent terminals in an iav>OpGCaMP6f;tdTomato animal. Coronal imaging section is indicated (white dashed line). The claw, a region that is implicated in FTi joint-encoding, is also indicated (white arrowhead). **(c)** Two-photon coronal section image of iav-Gal4 showing ROIs. ROI 2 is the claw proprioceptive region in panel b. Images were acquired at 4.3 fps as for the AN functional screen. Scale bar is 20 *μ*m. **(e)** ROIs for two-photon recordings from an R73D10>OpGCaMP6f;tdTomato animal. Here, a horizontal section was imaged at 4.25 fps. Scale bar is 20 *μ*m. **(f)** Schematic showing how resting epochs were extracted and concatenated for linear regression analysis with leg joint angles. **(g)** Proportion of proprioceptor activity variance that is uniquely explained by joint angle regressors (cross-validated ΔR2) for all of the data (left) or exclusively resting epochs (right). P-values report the one-tailed F-statistic of overall significance of the complete regression model with none of the regressors shuffled without adjustment for multiple comparisons (***p<0.001).

**Extended Data Fig. 5:**
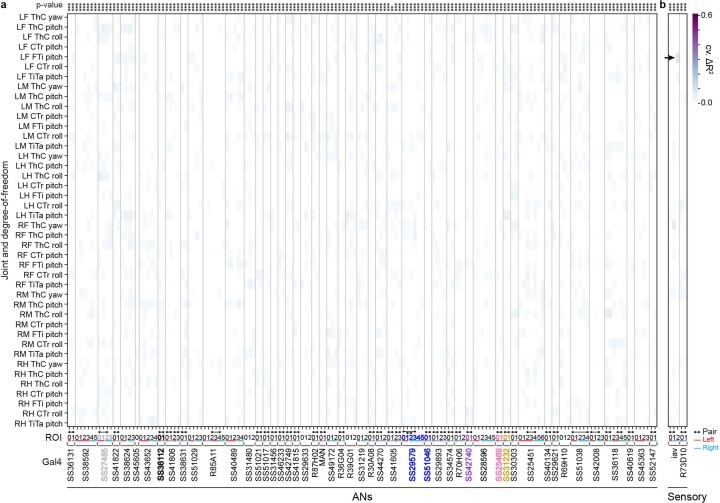
Joint angle encoding in Ascending Neurons and limb proprioceptors exclusively during resting epochs. Proportion of variance in **(a)** AN and **(b)** proprioceptor activity that is uniquely explained by joint angle regressors (cross-validated ΔR2 based on joint movements. P-values report the one-tailed F-statistic of overall significance of the complete regression model with none of the regressors shuffled without adjustment for multiple comparisons (**p<0.01 and ***p<0.001).

First, we examined to what extent individual joint angles could explain the activities of 157 ROIs. Notably, if two regressors are highly correlated, one regressor can compensate when shuffling the other, resulting in a potential false negative. Therefore, we confirmed that the vast majority of joint angles do not co-vary with others—with the exception of the middle and hindleg coxa-trochanter (CTr) and femur-tibia (FTi) pitch angles (Extended Data Fig. 3). We did not find any evidence of joint angles explaining AN activity (Fig. 2a). To assess the strength of this result, we performed a ‘positive’ control experiment by measuring joint angle encoding for limb proprioceptors (iav-Gal4 and R73D10-Gal4 animals^40^) during resting periods that have slow changes in limb position and, thus, do not suffer as strongly from the slow calcium indicator decay dynamics (Extended Data Fig. 4). These experiments yielded only weak joint angle encoding that was not much larger than that observed for ANs (Extended Data Fig. 5). Thus, there is either (1) widespread but weak joint angle encoding among many ANs or (2) noise-related/artifactual correlations between limb movements and neural activity. Owing to technical limitations in our recording and analysis approach, we cannot distinguish between these two possibilities, leaving open the degree to which ANs encode joint angles to more temporally precise approaches, such as electrophysiology.

Similarly, individual leg movements (tested by shuffling all of the joint angle regressors for a given leg) could not explain the variance of AN activity (Fig. 2b). Additionally, with the exception of ANs from SS25469, whose activities could be explained by movements of the front legs (Fig. 2c), AN activity largely could not be explained by the movements of pairs of legs. Notably, the activity of ANs could be explained by behavioral states (Fig. 2d). Most ANs encoded self-motion—forward walking and resting—but some also encoded discrete behavioral states, such as eye grooming, PEs and responses to puff stimuli.

**Extended Data Fig. 6:**
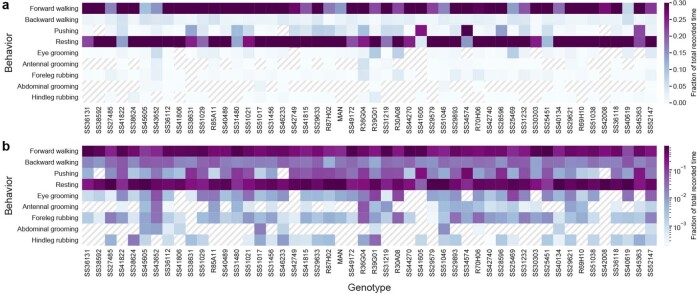
The degree to which representative animals for each genotype displayed each classified behavior. **(a)** Linear and **(b)** log color-coded quantification of the fraction of total recorded time that a representative animal for each spGal4 and Gal4 spent performing each classified behavior. Hashed lines indicate the absence of a behavior.

**Extended Data Fig. 7:**
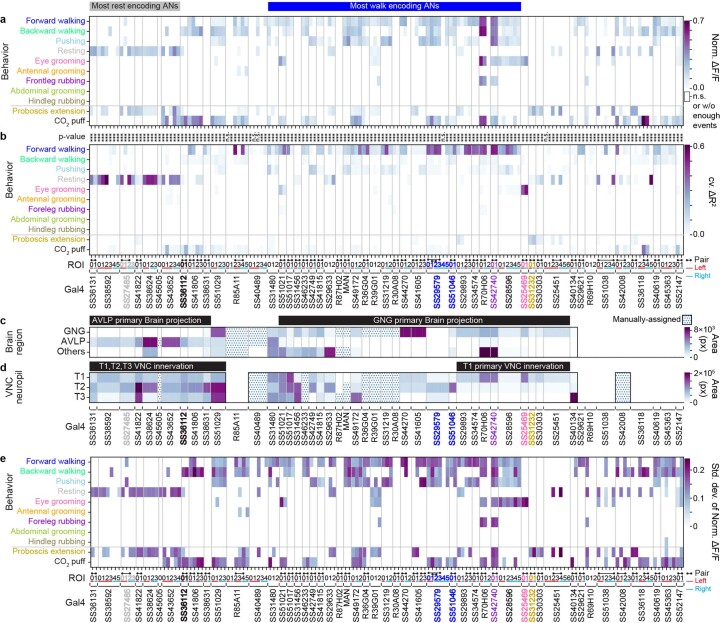
Normalized mean activity (ΔF/F) of ascending neurons during behaviors, and a summary of their behavioral encoding, brain targeting, and VNC patterning. **(a)** Normalized mean ΔF/F, normalized between 0 and 1, for a given AN across all epochs of a specific behavior. Analyses were performed for 157 ANs recorded from 50 driver lines. Note that fluorescence for non-orthogonal behaviors/events may overlap (for example, for backward walking and puff, or resting and proboscis extensions). Conditions with less than ten epochs longer than 0.7 s are masked (white). One-way ANOVA and two-sided posthoc Tukey tests to correct for multiple comparisons were performed to test if values are significantly different from baseline. Non-significant samples are also masked (white). **(b)** Variance in AN activity that can be uniquely explained by a regressor (cross-validated ΔR2) for behaviors as shown in Fig. 2d. Non-orthogonal regressors (PE and CO_2_ puffs) are separated from the others. P-values report the one-tailed F-statistic of overall significance of the complete regression model with no regressors shuffled without adjustment for multiple comparisons (*p<0.05, **p<0.01, and ***p<0.001). **(c,d)** The most substantial AN **(c)** targeting of brain regions, or **(d)** patterning of VNC regions, as quantified by pixel-based analysis of MCFO labelling. Driver lines that were manually quantified are indicated (dotted cells). Projections that could not be unambiguously identified are left blank. Notable encoding and innervation patterns are indicated by bars above each matrix. Lines (and their corresponding ANs) selected for more in-depth analysis are color-coded by the behavioral class that best explains their neural activity: SS27485 (resting), SS36112 (puff responses), SS29579 (walking), SS51046 (turning), SS42740 (foreleg-dependent behaviors), SS25469 (eye grooming), and SS31232 (proboscis extensions). **(e)** Standard deviation of normalized activity (ΔF/F), normalized between 0 and 1, for a given AN across all epochs of a specific behavior. Source data

We note that, because behaviors were generated spontaneously, some rare behaviors, such as abdominal grooming and hindleg rubbing, were not generated by representative animals for specific driver lines (Extended Data Fig. 6). Our regression approach is also inherently conservative: it avoids false positives, but it is, therefore, prone to false negatives for infrequently occurring behaviors. Therefore, as an additional, alternative approach, we measured the mean normalized Δ*F*/*F* of each AN for each behavioral state. Using this complementary approach, we confirmed and extended our results (Extended Data Fig. 7a). For example, in the case of MANs^25^, we found a more prominent expected^28^ encoding of pushing and backward walking as well as weaker encoding of forward walking (a very frequently generated behavior that often co-occurs with pushing). We considered both results from our linear regression as well as our mean normalized Δ*F*/*F* analyses when selecting neurons for further in-depth analysis.

### AN brain targeting as a function of encoding

Having identified the behavioral state encoding of a large population of 157 ROIs, we next wondered to what extent these distinct state signals are routed to specific and distinct brain targets (Fig. 1b). On the one hand, individual ANs might project diffusely to multiple brain regions. Alternatively, they might target one or only a few regions. To address these possibilities, we quantified the brain projections of all ANs by dissecting, immunostaining and imaging the expression of spFP and MCFO reporters in these neurons (Fig. 1e).

**Fig. 3:**
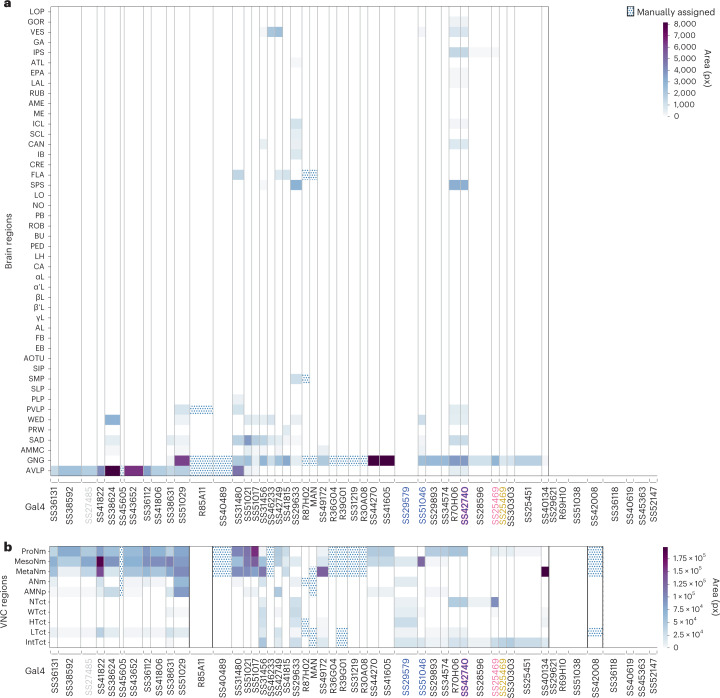
ANs principally project to the brain’s AVLP and GNG and the VNC’s leg neuromeres. Regional innervation of the brain (**a**) or the VNC (**b**). Data are for 157 ANs recorded from 50 driver lines and automatically quantified through pixel-based analyses of MCFO-labeled confocal images. Other, manually quantified driver lines are indicated (dotted). Lines for which projections could not be unambiguously identified are left blank. Lines selected for more in-depth evaluation are color-coded by the behavioral state that best explains their neural activity: SS27485 (resting), SS36112 (puff responses), SS29579 (walking), SS51046 (turning), SS42740 (foreleg-dependent behaviors), SS25469 (eye grooming) and SS31232 (PEs). Here, ROI numbers are not indicated because there is no one-to-one mapping between individual ROIs and MCFO-labeled single neurons. Source data

Strikingly, we found that AN projections to the brain were largely restricted to two regions: the AVLP, a site known for multimodal, integrative sensory processing^31–36^, and the GNG, a hub for action selection^24,37,38^ (Fig. 3a). ANs encoding resting and puff responses almost exclusively target the AVLP (Extended Data Fig. 7b,c), providing a means for interpreting whether sensory cues arise from self-motion or the movement of objects in the external environment. By contrast, the GNG is targeted by ANs encoding a wide variety of behavioral states, including walking, eye grooming and PEs (Extended Data Fig. 7b,c). These signals may help to ensure that future behaviors are compatible with ongoing ones.

Because AN dendrites and axons within the VNC might be used to compute behavioral state encodings, we next asked to what extent their projection patterns within the VNC are predictive of an AN’s encoding. For example, ANs encoding resting might require sampling each VNC leg neuromere (T1, T2 and T3) to confirm that every leg is inactive. By quantifying AN projections within the VNC (Fig. 3b), we found that, indeed, ANs encoding resting (for example, SS27485) each project to all VNC leg neuromeres (Extended Data Fig. 7b,d). By contrast, ANs encoding foreleg-dependent eye grooming (SS25469) project only to T1 VNC neuromeres that control the front legs (Extended Data Fig. 7b,d). To more deeply understand how the morphological features of ANs relate to behavioral state encoding, we next performed a detailed study of a diverse subset of ANs.

### Rest encoding and puff response encoding by morphologically similar ANs

**Fig. 4:**
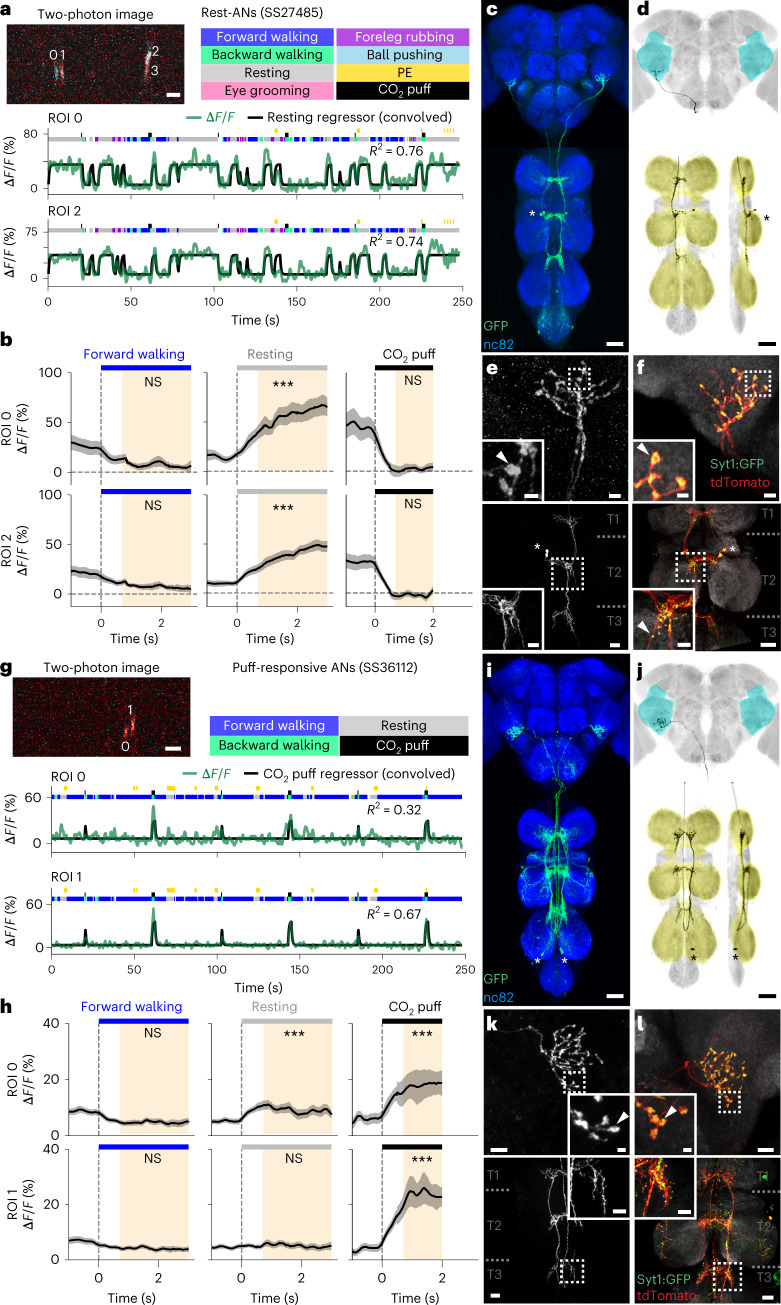
Functional and anatomical properties of ANs that encode resting or responses to puffs. **a**,**g**, Top left: two-photon image of axons from an SS27485-Gal4 (**a**) or an SS36112-Gal4 (**g**) animal expressing OpGCaMP6f (cyan) and tdTomato (red). ROIs are numbered. Scale bars, 5 μm. Bottom: behavioral epochs are color-coded. Representative Δ*F*/*F* time series from two ROIs (green) overlaid with a prediction (black) obtained by convolving resting epochs (**a**) or puff stimuli (**g**) with Ca^2+^ indicator response functions. Explained variances are indicated (*R*^2^). **b**,**h**, Mean (solid line) and 95% confidence interval (gray shading) of Δ*F*/*F* traces for rest ANs (**b**) or puff-responsive ANs (**h**) during epochs of forward walking (left), resting (middle) or CO_2_ puffs (right). 0 s indicates the start of each epoch. Data more than 0.7 s after onset (yellow region) are compared with an Otsu thresholded baseline (one-way ANOVA and two-sided Tukey post hoc comparison, ***P < 0.001, **P < 0.01, *P < 0.05, NS, not significant). **c**,**i**, Standard deviation projection image of an SS27485-Gal4 (**c**) or an SS36112-Gal4 (**i**) nervous system expressing smFP and stained for GFP (green) and Nc82 (blue). Cell bodies are indicated (white asterisk). Scale bars, 40 μm. **d**,**j**, Projection as in **c** and **i** but for one MCFO-expressing, traced neuron (black asterisk). The brain’s AVLP (cyan) and the VNC’s leg neuromeres (yellow) are color-coded. Scale bars, 40 μm. **e**,**f**,**k**,**l**, Higher magnification projections of brains (top) and VNCs (bottom) from SS27485-Gal4 (**e**,**f**) or SS36112-Gal4 (**k**,**l**) animals expressing the stochastic label MCFO (**e**,**k**) or the synaptic marker, syt:GFP (green) and tdTomato (red) (**f**,**l**). Insets magnify dashed boxes. Indicated are cell bodies (asterisks), bouton-like structures (white arrowheads) and VNC leg neuromeres (T1, T2 and T3). Scale bars for brain images and insets are 5 μm (**e**) or 10 μm (**k**) and 2 μm for insets. Scale bars for VNC images and insets are 20 μm and 10 μm, respectively. Source data

**Extended Data Fig. 8:**
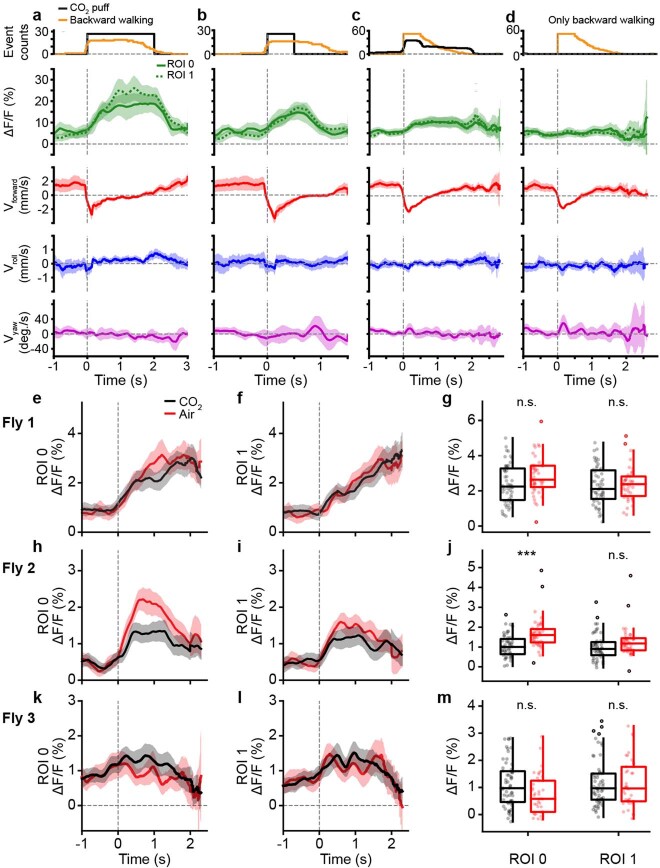
Puff-responsive-ANs do not encode backward walking and respond similarly to puffs of air, or CO_2_. **(a-d)** Puff-responsive-ANs (SS36112) activity (green) and corresponding spherical treadmill rotational velocities (red, blue, and purple) during **(a)** long, 2 s CO_2_-puff stimulation (black) and associated backward walking (orange), **(b)** short, 0.5 s CO_2_-puff stimulation, **(c)** periods with backward walking, and **(d)** the same backward walking events as in c but only during periods without coincident puff stimulation. Shown are the mean (solid and dashed lines) and 95% confidence interval (shaded areas) of multiple ΔF/F and ball rotation time-series. **(e-m)** Activity of puff-responsive-ANs (SS36112) from three flies (**e-g, h-j**, and **k-m**, respectively) in response to puffs of air (red), or CO_2_ (black). (**e-f, h-i, k-l**) Shown are mean (solid and dashed lines) and 95% confidence interval (shaded areas) ΔF/F for ROIs **(e,h,k)** 0 and **(f,i,l)** 1. **(g,j,m)** Mean fluorescence (circles) of traces for ROIs 0 (left) or 1 (right) from 0.7 s after puff onset until the end of stimulation. Overlaid are box plots representing the median, interquartile range (IQR), and 1.5 IQR. Outliers beyond 1.5 IQR are indicated (opaque circles). N = **(g)** 54 for CO_2_ and 43 for air **(j)** 48 for CO_2_ and 45 for air, and **(m)** 58 for CO_2_ and 37 for air-puff epochs. A two-sided Mann-Whitney test (*** p<0.001, ** p<0.01, * p<0.05) was used to compare responses to puffs of CO_2_ (red), or air (black). Source data

AN classes that encode resting and puff-elicited responses have coarsely similar projection patterns: both almost exclusively target the brain’s AVLP while also sampling from all three VNC leg neuromeres (T1–T3) (Extended Data Fig. 7). We next investigated which more detailed morphological features might be predictive of their very distinct encoding by closely examining the functional and morphological properties of specific pairs of ‘rest ANs’ (SS27485) and ‘puff-responsive ANs’ (SS36112). Neural activity traces of rest ANs and puff-responsive ANs could be reliably predicted by regressors for resting (Fig. 4a) and puff stimuli (Fig. 4g), respectively. This was statistically confirmed by comparing behavior-triggered averages of AN responses at the onset of resting (Fig. 4b) versus puff stimulation (Fig. 4h), respectively. Notably, although CO_2_ puffs frequently elicited brief periods of backward walking, close analysis revealed that puff-responsive ANs primarily respond to gust-like puffs and do not encode backward walking (Extended Data Fig. 8a–d). They also did not encode responses to CO_2_ specifically: the same neurons responded equally well to puffs of air (Extended Data Fig. 8e–m).

As mentioned, rest ANs and puff-responsive ANs, despite their very distinct encoding, exhibit similar innervation patterns in the brain and VNC. However, MCFO-based single-neuron analysis revealed a few subtle but potentially important differences. First, rest AN and puff AN cell bodies are located in the T2 (Fig. 4c) and T3 (Fig. 4i) neuromeres, respectively. Second, although both AN classes project medially into all three leg neuromeres (T1–T3), rest ANs have a simpler morphology (Fig. 4d) than the more complex arborizations of puff-responsive ANs in the VNC (Fig. 4j). In the brain, both AN types project to nearly the same ventral region of the AVLP where they have varicose terminals (Fig. 4e,k). Using syt:GFP, a GFP-tagged synaptotagmin (presynaptic) marker, we confirmed that these varicosities house synapses (Fig. 4f, top, and Fig. 4l, top). Notably, in addition to smooth, likely dendritic arbors, both AN classes have axon terminals within the VNC (Fig. 4f, bottom, and Fig. 4l, bottom).

Taken together, these results demonstrate that even very subtle differences in VNC patterning can give rise to markedly different AN tuning properties. In the case of rest ANs and puff-responsive ANs, we speculate that this might be due to physically close but distinct presynaptic partners—possibly leg proprioceptive afferents for rest ANs and leg tactile afferents for puff-responsive ANs.

### Walk encoding or turn encoding correlates with VNC projections

**Fig. 5:**
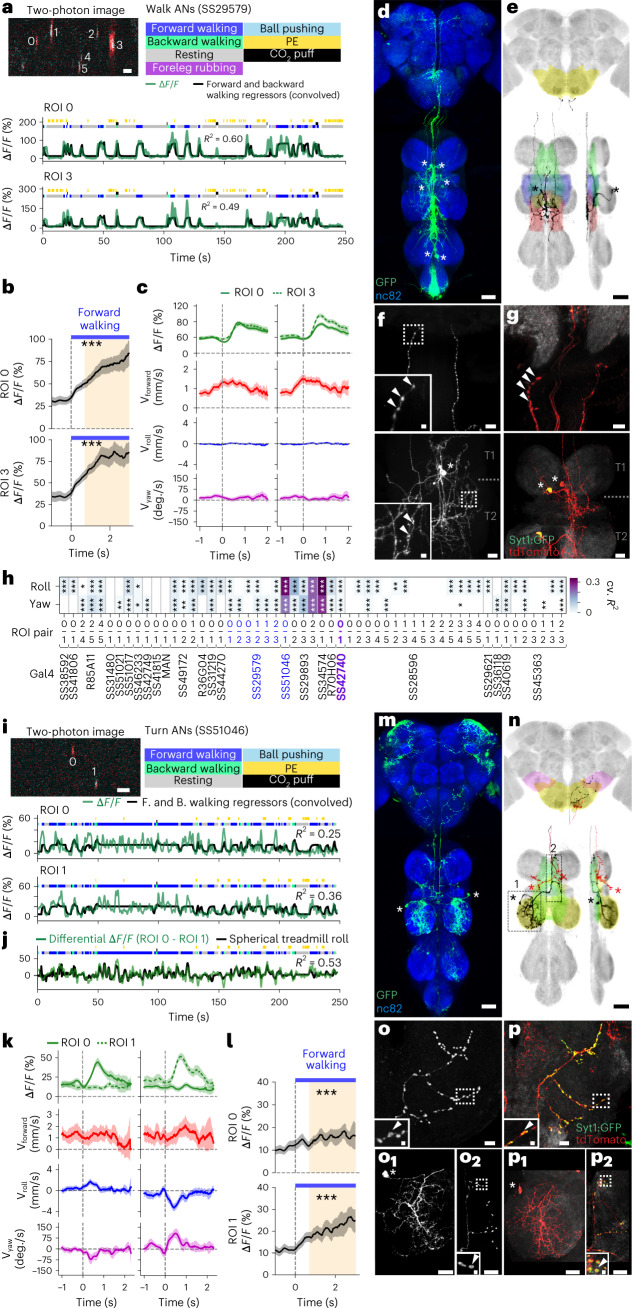
Functional and anatomical properties of ANs that encode walking or turning. **a**,**i**, Top left: two-photon image of axons from an S29579-Gal4 (**a**) or an SS51046-Gal4 (**i**) animal expressing OpGCaMP6f (cyan) and tdTomato (red). ROIs are numbered. Scale bars, 5 μm. Bottom: behavioral epochs are color-coded. Representative Δ*F*/*F* time series from two ROIs (green) overlaid with a prediction (black) obtained by convolving forward and backward walking epochs with Ca^2+^ indicator response functions. Explained variance is indicated (*R*^2^). **b**,**l**, Mean (solid line) and 95% confidence interval (gray shading) of Δ*F*/*F* traces during epochs of forward walking. 0 s indicates the start of each epoch. Data more than 0.7 s after onset (yellow region) are compared with an Otsu thresholded baseline (one-way ANOVA and two-sided Tukey post hoc comparison, ***P < 0.001, **P < 0.01, *P < 0.05, NS, not significant). **c**,**k**, Fluorescence (OpGCaMP6f) event-triggered average ball rotations for ROI 0 (left) or ROI 3 (right) of an SS29579-Gal4 animal (**c**) or ROI 0 (left) or ROI 1 (right) of an SS51046-Gal4 animal (**k**). Fluorescence events are time-locked to 0 s (green). Shown are mean and 95% confidence intervals for forward (red), roll (blue) and yaw (purple) ball rotational velocities. **d**,**m**, Standard deviation projection image for an SS29579-Gal4 (**d**) or an SS51046 (**m**) nervous system expressing smFP and stained for GFP (green) and Nc82 (blue). Cell bodies are indicated (white asterisks). Scale bar, 40 μm. **e**,**n**, Projection as in **d** and **m** but for one MCFO-expressing, traced neuron (black asterisks). The brain’s GNG (yellow) and WED (pink) and the VNC’s intermediate (green), wing (blue), haltere (red), tectulum and mesothoracic leg neuromere (yellow) are color-coded. Scale bar, 40 μm. **f**,**g**,**o**,**p**, Higher magnification projections of brains (top) and VNCs (bottom) of SS29579-Gal4 (**f**,**g**) or SS51046-Gal4 (**o**,**p**) animals expressing the stochastic label MCFO (**f**,**o**) or the synaptic marker, syt:GFP (green) and tdTomato (red) (**g**,**p**). Insets magnify dashed boxes. Indicated are cell bodies (asterisks), bouton-like structures (white arrowheads) and VNC leg neuromeres (T1 and T2). **o**_**1**_ and **p**_**1**_ or **o**_**2**_ and **p**_**2**_ correspond to locations 1 and 2 in **n**. Scale bars for brain images and insets are 10 μm and 2 μm, respectively. Scale bars for VNC images and insets are 20 μm and 4 μm, respectively. **h**, Quantification of the degree to which the difference in pairwise activity of ROIs for multiple AN driver lines can be explained by spherical treadmill yaw or roll velocity—a proxy for turning. *P* values report the one-tailed *F*-statistic of overall significance of the complete regression model with none of the regressors shuffled (*P < 0.05, **P < 0.01 and ***P < 0.001). Source data

Among the ANs that we analyzed, most encode walking (Fig. 2d). We asked whether an AN’s patterning within the VNC may predict its encoding of locomotion generally (for example, walking irrespective of kinematics) or specifically (for example, turning in a particular direction). Indeed, we observed that, whereas the activity of one pair of ANs (SS29579, ‘walk ANs’) was remarkably well explained by the timing and onset of walking epochs (Fig. 5a–c), for other ANs, a simple walking regressor could account for much less of the variance in neural activity (Fig. 2d). We reasoned that these ANs might, instead, encode narrower locomotor dimensions, such as turning. For a bilateral pair of DNa01 DNs, their difference in activity correlates with turning direction^28,41^. To see if this relationship might also hold for some pairs of walk-encoding ANs, we quantified the degree to which the difference in pairwise activity can be explained by spherical treadmill yaw or roll velocity—a proxy for turning (Fig. 5h). Indeed, we found several pairs of ANs for which turning explained a relatively large amount of variance. For one pair of ‘turn ANs’ (SS51046), although a combination of forward and backward walking regressors poorly predicted neural activity (Fig. 5i), a regressor based on spherical treadmill roll velocity strongly predicted the pairwise difference in neural activity (Fig. 5j). When an animal turned right, the right (ipsilateral) turn AN was more active, and the left turn AN was more active during left turns (Fig. 5k). During forward walking, both turn ANs were active (Fig. 5l).

We next asked how VNC patterning might predict this distinction between general (walk ANs) versus specific (turn ANs) locomotor encoding. Both AN classes have cell bodies in the VNC’s T2 neuromere (Fig. 5d,m). However, walk ANs bilaterally innervate the T2 neuromere (Fig. 5e), whereas turn ANs unilaterally innervate T1 and T2 (Fig. 5n, black). Their ipsilateral T2 projections are smooth and likely dendritic (Fig. 5o_1_,p_1_), whereas their contralateral T1 projections are varicose and exhibit syt:GFP puncta, suggesting that they harbor presynaptic terminals (Fig. 5o_2_,p_2_). Both walk ANs (Fig. 5d,e) and turn ANs (Fig. 5m,n) project to the brain’s GNG. However, only turn ANs project to the WED (Fig. 5n). Notably, walk AN terminals in the brain (Fig. 5f) are not labeled by syt:GFP (Fig. 5g), suggesting that they may be neuromodulatory in nature.

**Extended Data Fig. 9:**
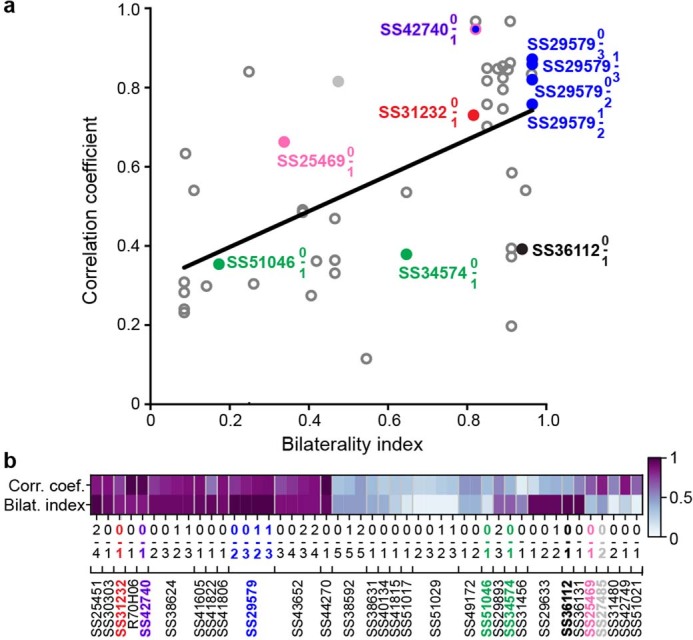
The bilaterality of an ascending neuron pair’s VNC patterning correlates with the synchrony of their activity. **(a)** A bilaterality index, quantifying the differential innervation of the left and right VNC (without distinguishing between axons and dendrites) is compared with the Pearson correlation coefficient computed for the activity of left and right ANs for a driver line pair (R2 = 0.31 and p<0.001 using a two-sided Wald Test with a t-distribution to test whether to reject the null hypothesis that the coefficient of a linear equation equals 0). **(b)** Bilaterality index and Pearson correlation coefficient values for each AN pair. Source data

These data support the notion that general versus specific AN behavioral state encoding may depend on the laterality of VNC patterning. Additionally, whereas pairs of broadly tuned walk ANs that bilaterally innervate the VNC are synchronously active, pairs of narrowly tuned turn ANs are asynchronously active (Extended Data Fig. 9).

### Foreleg-dependent behaviors encoded by anterior VNC ANs

In addition to locomotion, flies use their forelegs to perform complex movements, including reaching, boxing, courtship tapping and several kinds of grooming. An ongoing awareness of these behavioral states is critical to select appropriate future behaviors that do not lead to unstable postures. For example, before deciding to groom its hindlegs, an animal must first confirm that its forelegs are stably on the ground and not also grooming.

**Fig. 6:**
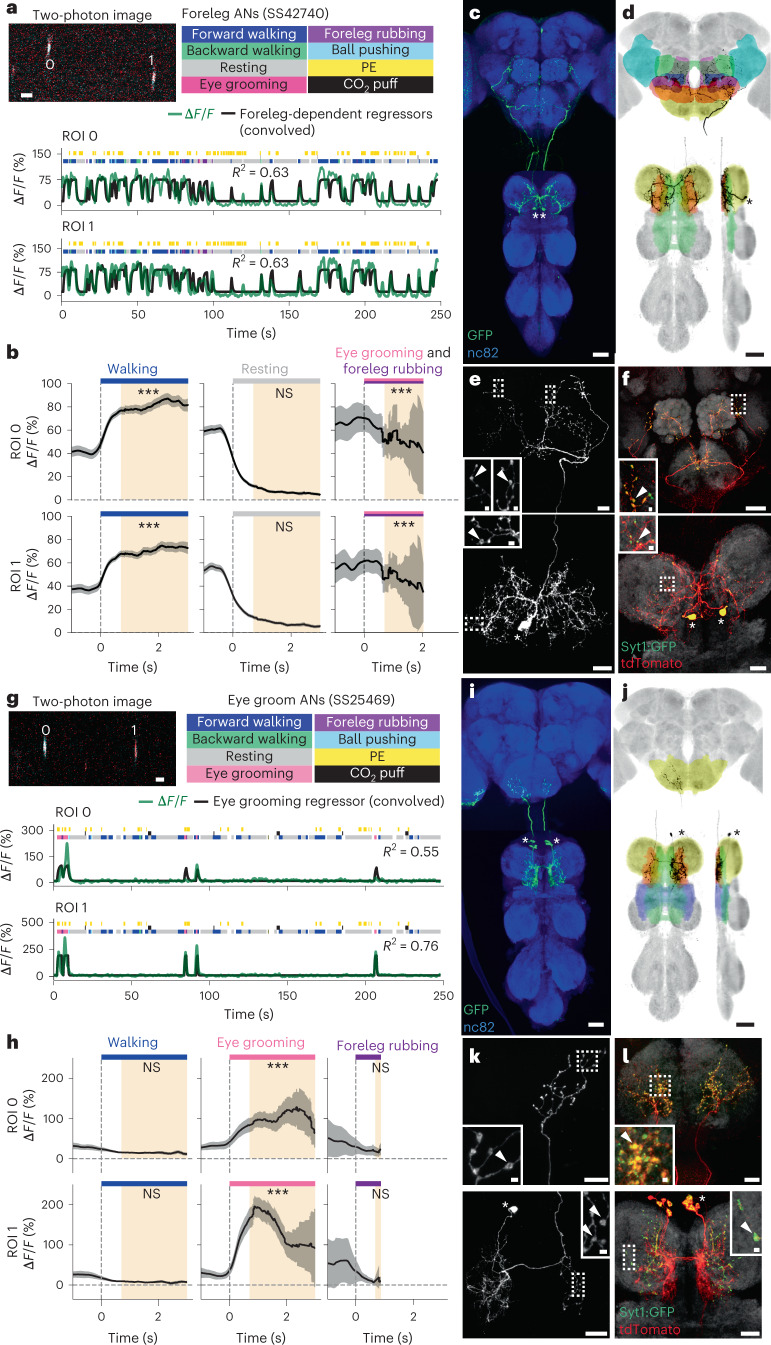
Functional and anatomical properties of ANs that encode multiple foreleg behaviors or only eye grooming. **a**,**g**, Top left: two-photon image of axons from an SS42740-Gal4 (**a**) or an SS25469-Gal4 (**g**) animal expressing OpGCaMP6f (cyan) and tdTomato (red). ROIs are numbered. Scale bar, 5 μm. Bottom: behavioral epochs are color-coded. Representative Δ*F*/*F* time series from two ROIs (green) overlaid with a prediction (black) obtained by convolving all foreleg-dependent behavioral epochs (forward and backward walking as well as eye, antennal and foreleg grooming) for an SS42740-Gal4 animal (**a**) or eye grooming epochs for an SS25469-Gal4 animal (**g**) with Ca^2+^ indicator response functions. Explained variance is indicated (*R*^2^). **b**,**h**, Mean (solid line) and 95% confidence interval (gray shading) of Δ*F*/*F* traces for foreleg ANs (**b**) during epochs of forward walking (left), resting (middle) or eye grooming and foreleg rubbing (right) or eye groom ANs (**h**) during forward walking (left), eye grooming (middle) or foreleg rubbing (right) epochs. 0 s indicates the start of each epoch. Data more than 0.7 s after onset (yellow region) are compared with an Otsu thresholded baseline (one-way ANOVA and two-sided Tukey post hoc comparison, ***P < 0.001, **P < 0.01, *P < 0.05, NS, not significant). **c**,**i**, Standard deviation projection image for an SS42740-Gal4 (**c**) or an SS27485-Gal4 (**i**) nervous system expressing smFP and stained for GFP (green) and Nc82 (blue). Cell bodies are indicated (white asterisks). Scale bars, 40 μm. **d**,**j**, Projections as in **c** and **i** but for one MCFO-expressing, traced neuron (black asterisks). The brain’s GNG (yellow), AVLP (cyan), SAD (green), VES (pink), IPS (blue) and SPS (orange) and the VNC’s neck (orange), intermediate tectulum (green), wing tectulum (blue) and prothoracic leg neuromere (yellow) are color-coded. Scale bars, 40 μm. **e**,**f**,**k**,**l**, Higher magnification projections of brains (top) and VNCs (bottom) from SS42740-Gal4 (**e**,**f**) or SS25469-Gal4 (**k**,**l**) animals expressing the stochastic label MCFO (**e**,**k**) or the synaptic marker, syt:GFP (green) and tdTomato (red) (**f**,**l**). Insets magnify dashed boxes. Indicated are cell bodies (asterisks) and bouton-like structures (white arrowheads). Scale bars for brain images and insets are 20 μm and 2 μm, respectively. Scale bars for VNC images and insets are 20 μm and 2 μm, respectively. Source data

We noted that some ANs project only to the VNC’s anterior-most, T1 leg neuromere (Extended Data Fig. 7d). This pattern implies a potential role in encoding behaviors that depend only on the forelegs. Indeed, close examination revealed two classes of ANs that encode foreleg-related behaviors. We found ANs (SS42740) that were active during multiple foreleg-dependent behaviors, including walking, pushing and grooming (‘foreleg ANs’; overlaps with R70H06) (Extended Data Fig. 7a and Fig. 6a,b). By contrast, another pair of ANs (SS25469) was narrowly tuned and sometimes asynchronously active only during eye grooming (‘eye groom ANs’) (Extended Data Fig. 7a,b and Fig. 6g,h). Similarly to walking and turning, we hypothesized that this general (foreleg) versus specific (eye groom) behavioral encoding might be reflected by a difference in the promiscuity and laterality of AN innervations in the VNC.

To test this hypothesis, we compared the morphologies of foreleg and eye groom ANs. Both had cell bodies in the T1 neuromere, although foreleg ANs were posterior (Fig. 6c), and eye groom ANs were anterior (Fig. 6i). Foreleg ANs and eye groom ANs also both projected to the dorsal T1 neuromere, with eye groom AN neurites restricted to the tectulum (Fig. 6d,j). Notably, foreleg AN puncta (Fig. 6e, bottom) and syt:GFP expression (Fig. 6f, bottom) were bilateral and diffuse, whereas eye groom AN puncta (Fig. 6k, bottom) and syt:GFP expression (Fig. 6l, bottom) were largely restricted to the contralateral T1 neuromere. Projections to the brain paralleled this difference in VNC projection promiscuity: foreleg ANs terminated across multiple brain areas—GNG, AVLP, SAD, VES, IPS and SPS (Fig. 6e,f, top)— whereas eye groom ANs narrowly targeted the GNG (Fig. 6k,l, top).

These results further illustrate how an AN’s encoding relates to its VNC patterning. Here, diffuse, bilateral projections are associated with encoding multiple behavioral states that require foreleg movements, whereas focal, unilateral projections are related to a narrow encoding of eye grooming.

### Temporal integration of PEs by an AN cluster

**Fig. 7:**
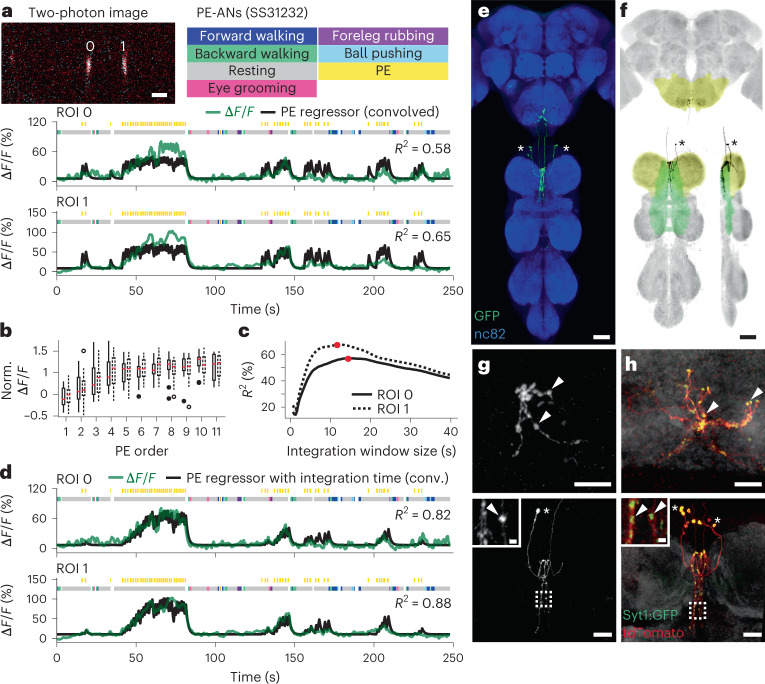
Functional and anatomical properties of ANs that integrate the number of PEs over time. **a**, Top left: two-photon image of axons from an SS31232-Gal4 animal expressing OpGCaMP6f (cyan) and tdTomato (red). ROIs are numbered. Scale bar, 5 μm. Bottom: behavioral epochs are color-coded. Representative Δ*F*/*F* time series from two ROIs (green) overlaid with a prediction (black) obtained by convolving PE epochs with a Ca^2+^ indicator response function. Explained variance is indicated (*R*^2^). **b**, Δ*F*/*F*, normalized with respect to the neuron’s 90th percentile, as a function of PE number within a PE train for ROIs 0 (solid boxes, filled circles) or 1 (dashed boxes, open circles). Data include 25 PE trains from eight animals and are presented as IQR (box), median (center), 1.5× IQR (whisker) and outliers (circles). **c**, Explained variance (*R*^2^) between Δ*F*/*F* time series and a prediction obtained by convolving PE epochs with a Ca^2+^ indicator response function and a time window. Time windows that maximize the correlation for ROIs 0 (solid line) and 1 (dashed line) are indicated (red circles). **d**, Behavioral epochs are color-coded. Representative Δ*F*/*F* time series from two ROIs (green) are overlaid with a prediction (black) obtained by convolving PE epochs with a Ca^2+^ response function as well as the time windows indicated in **c** (red circles). Explained variance is indicated (*R*^2^). **e**, Standard deviation projection image of a SS31232-Gal4 nervous system expressing smFP and stained for GFP (green) and Nc82 (blue). Cell bodies are indicated (white asterisks). Scale bar, 40 μm. **f**, Projection as in **e** but for one MCFO-expressing, traced neuron (black asterisks). The brain’s GNG (yellow) and the VNC’s intermediate tectulum (green) and prothoracic leg neuromere (yellow) are color-coded. Scale bar, 40 μm. **g**,**h**, Higher magnification projections of brains (top) and VNCs (bottom) for SS31232-Gal4 animals expressing the stochastic label MCFO (**g**) or the synaptic marker, syt:GFP (green) and tdTomato (red) (**h**). Insets magnify dashed boxes. Indicated are cell bodies (asterisks) and bouton-like structures (white arrowheads). Scale bars for brain images are 10 μm. Scale bars for VNC images and insets are 20 μm and 2 μm, respectively. Source data

Flies often generate spontaneous PEs while resting (Fig. 7a, yellow ticks). We observed that PE-ANs (SS31232, overlap with SS30303) (Fig. 2d) become active during PE trains—a sequence of PEs that occurs within a short period of time (Fig. 7a). Close examination revealed that PE-AN activity slowly ramped up over the course of PE trains. This made them difficult to model using a simple PE regressor: their activity levels were lower than predicted early in PE trains and higher than predicted late in PE trains. On average, across many PE trains, PE-AN activity reached a plateau by the seventh PE (Fig. 7b).

Thus, PE-AN activity seemed to convey the temporal integration of discrete events^42,43^. Therefore, we next asked if PE-AN activity might be better predicted using a regressor that integrates the number of PEs within a given time window. The most accurate prediction of PE-AN dynamics could be obtained using an integration window of more than 10 s (Fig. 7c, red circles), making it possible to predict both the undershoot and overshoot of PE-AN activity at the start and end of PE trains, respectively (Fig. 7d).

Temporal integration can be implemented using a line attractor model^44,45^ based on recurrently connected circuits. To explore the degree to which PE-AN might support an integration of PE events via recurrent interconnectivity, we examined PE-AN morphologies more closely. PE-AN cell bodies were located in the anterior T1 neuromere (Fig. 7e). From there, they projected dense neurites into the midline of the T1 neuromere (Fig. 7f). Among these neurites in the VNC, we observed puncta and syt:GFP expression consistent with presynaptic terminals (Fig. 7g,h, bottom). Their dense and highly overlapping arbors would be consistent with interconnectivity between PE-ANs, enabling an integration that may filter out sparse PE events associated with feeding and allow PE-ANs to convey long PE trains observed during deep rest states^46^ to the brain’s GNG (Fig. 7g,h, top).

## Discussion

**Fig. 8:**
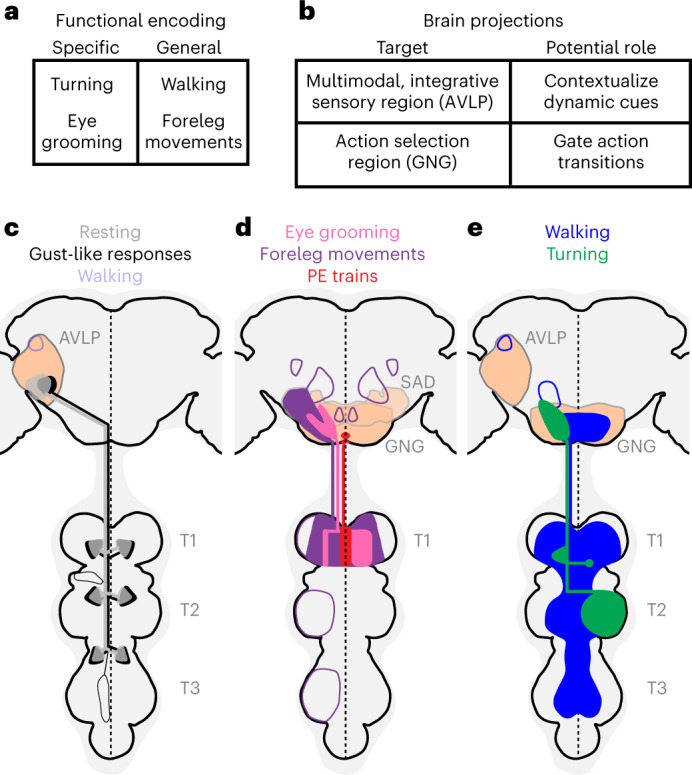
Summary of AN functional encoding, brain targeting and VNC patterning. **a**, ANs encode behavioral states in a specific (for example, eye grooming) or general (for example, any foreleg movement) manner. **b**, Corresponding anatomical analysis shows that ANs primarily target the AVLP, a multimodal, integrative brain region, and the GNG, a region associated with action selection. **c**,**d**, By comparing functional encoding with brain targeting and VNC patterning, we found that signals critical for contextualizing object motion—walking, resting and gust-like stimuli—are sent to the AVLP (**c**), whereas signals indicating diverse ongoing behavioral states are sent to the GNG (**d**), potentially to influence future action selection. **e**, Broad (for example, walking) or narrow (for example, turning) behavioral encoding is associated with diffuse and bilateral or restricted and unilateral VNC innervations, respectively. **c**–**e**, AN projections are color-coded by behavioral encoding. Axons and dendrites are not distinguished from one another. Brain and VNC regions are labeled. Frequently innervated brain regions—the GNG and AVLP—are highlighted (light orange). Less frequently innervated areas are outlined. The midline of the central nervous system is indicated (dashed line).

Animals must be aware of their own behavioral states to accurately interpret sensory cues and select appropriate future behaviors. In this study, we examined how this self-awareness might be conveyed to the brain by studying the activity and targeting of ANs in the *Drosophila* motor system. We discovered that ANs functionally encode behavioral states (Fig. 8a), predominantly those related to self-motion, such as walking and resting. The prevalence of AN walk encoding may represent an important source of global locomotor signals observed in the brain^9,47,48^. These encodings could be further distinguished as either general (for example, walk ANs that are active irrespective of particular locomotor kinematics and foreleg ANs that are active irrespective of foreleg kinematics) or specific (for example, turn ANs and eye groom ANs). Similarly, neurons in the vertebrate dorsal spinocerebellar tract have been shown to be more responsive to whole limb versus individual joint movements^49^. However, we note an important limitation: the time scales of calcium signals with a decay time constant on the order of 1 s (ref. ^50^) are not well matched to the time scales of leg movements, which, during very fast walking, can cycle every 25 ms (ref. ^24^). To partly compensate for the technical hurdle of relating relatively rapid joint movements to slow calcium indicator decay kinetics, we convolved joint angle time series with a kernel that would maximize the explanatory power of our regression analyses. Additionally, we confirmed that potential issues related to the non-orthogonality of joint angles and leg movements would not obscure our ability to explain the variance of AN neural activity (Extended Data Fig. 3). Our observation that eye groom AN activity could be explained by movements of the forelegs gave us further confidence that some leg movement encoding was detectable in our functional screen (Fig. 2c). However, to verify the relative absence of AN leg movement encoding, future work could use faster neural recording approaches or directly manipulate the legs of restrained animals while performing electrophysiological recordings of AN activity^40^.

We found that most ANs do not project diffusely across the brain but, rather, specifically target either the AVLP or the GNG (Fig. 8b). We hypothesize that this may reflect the contribution of AN behavioral state signals to two fundamental brain computations. First, the AVLP is a site known for multimodal, integrative sensory convergence^31–36^. However, we note that only a few studies have examined the functional role of this brain region. We speculate that the projection of ANs encoding resting, walking and gust-like puffs to the AVLP (Fig. 8c) may serve to contextualize time-varying sensory signals to indicate if they arise from self-motion or from objects moving and odors fluctuating in the world. A similar role—conveying self-motion—has been proposed for neurons in the vertebrate dorsal spinocerebellar tract^18^. Second, the GNG is thought to be an action selection center with a substantial innervation by DNs^37,38^ and other ANs^24^. It should be cautioned, however, that relatively little is known about this brain region—and the greater subesophageal zone (SEZ)—beyond its role in taste processing. Nevertheless, here we propose that the projection of ANs encoding diverse behavioral states (Fig. 8d,e) to the GNG may contribute to the computation of whether potential future behaviors are compatible with ongoing ones. This role would be consistent with a hierarchical control approach used in robotics^2^.

Notably, the GNG is also heavily innervated by DNs. Because ANs and DNs both contribute to action selection^24,25,38,51^, we speculate that they may connect within the GNG, forming a feedback loop between the brain and motor system. Specifically, ANs that encode specific behavioral states might excite DNs that drive the same behaviors to generate persistence while also suppressing DNs that drive conflicting behaviors. For example, turn ANs may excite DNa01 and DNa02, which control turning^28,41,52^, and foreleg ANs may excite aDN1 and aDN2, which control grooming^53^. This hypothesis may soon be tested using connectomics datasets^54–56^.

The morphology of an AN’s neurites in the VNC is, to some degree, predictive of its encoding (Fig. 8c–e). We observed this in several ways. First, ANs innervating all three leg neuromeres (T1, T2 and T3) encode global self-motion—walking, resting and gust-like puffs. Thus, rest ANs may sample from motor neurons driving the limb muscle tone needed to maintain a natural resting posture. Alternatively, based on their morphological overlap with femoral chordotonal organs (limb proprioception) afferents^21^ (Fig. 4c), they may be tonically active and then inhibited by joint movement sensing. By contrast, ANs with more restricted projections to one neuromere (T1 or T2) encode discrete behavioral states—turning, eye grooming, foreleg movements and PEs. This might reflect the cost of neural wiring, a constraint that may encourage a neuron to sample the minimal sensory and motor information required to compute a particular behavioral state. For example, to specifically encode eye grooming, these ANs may sample from T1 motor neurons driving cyclical CTr roll movements that are uniquely observed during eye grooming^57^. This is supported by our observation that the front leg pair and, to some degree, right front leg movements alone can account for activity in these neurons (Fig. 2a–c), and this behavior is highly correlated with CTr roll (Extended Data Fig. 3). To confirm this, future efforts should include electrophysiological recordings of eye groom ANs in restrained animals during magnetically controlled joint movements^21,40^. Second, general ANs (encoding walking and foreleg-dependent behaviors) exhibited bilateral projections in the VNC, whereas narrowly tuned ANs (encoding turning and eye grooming) exhibited unilateral and smooth, putatively dendritic projections. This was correlated with the degree of synchrony in the activity of pairs of ANs (Extended Data Fig. 9).

For all ANs that we examined in depth, we found evidence of axon terminals within the VNC. Thus, ANs may not simply relay behavioral state signals to the brain but may also perform other roles. For example, they might contribute to motor control as components of central pattern generators (CPGs) that generate rhythmic movements^58^. Similarly, rest ANs might control the limb muscle tone needed to maintain a natural resting posture. ANs might also participate in computing behavioral states. For example, here we speculate that recurrent interconnectivity among PE-ANs might give rise to their temporal integration and encoding of PE number^44,45^. Finally, ANs might contribute to action selection within the VNC. For example, eye groom ANs might project to the contralateral T1 neuromere to suppress circuits driving other foreleg-dependent behaviors, such as walking and foreleg rubbing.

**Extended Data Fig. 10:**
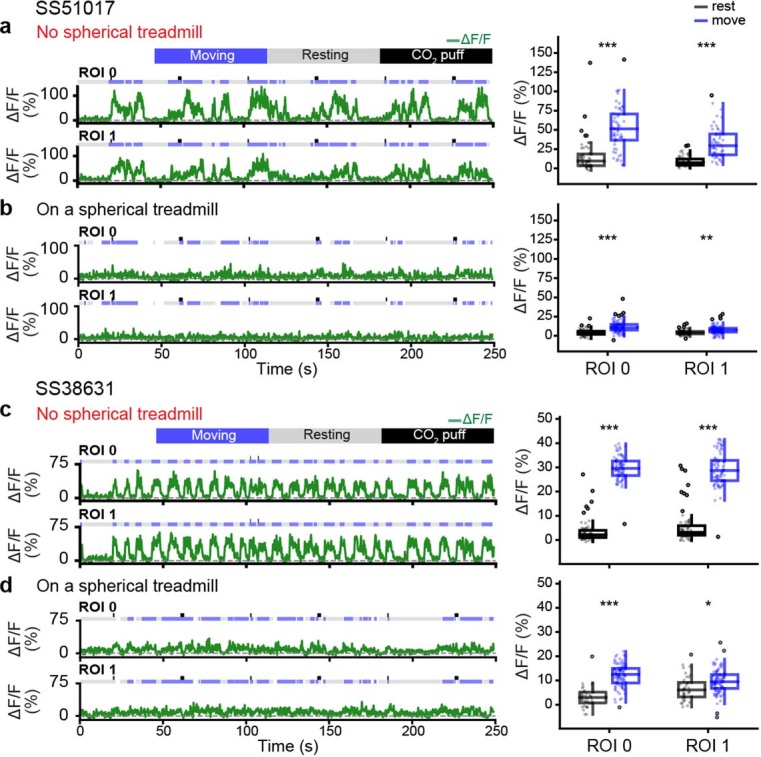
Ascending neurons that become active only when the spherical treadmill is removed. Representative AN recordings from ROIs 0 and 1 for one **(a,b)** SS51017-spGal4, or one **(c,d)** SS38631-spGal4 animal measured when it is **(a,c)** suspended without a spherical treadmill, or in contact with the spherical treadmill. Moving, resting, and puff stimulation epochs are indicated. Shown are (left) representative neural activity traces and (right) summary data including the median, interquartile range (IQR), and 1.5 IQR of AN ΔF/F values for N = **(a)** 55 and 56, **(b)** 80 and 102, **(c)** 77 and 76, **(d)** 38 and 97 epochs when the animals are resting (black) and moving (blue), respectively. Outliers (values beyond 1.5 IQR) are indicated (opaque circles). Statistical comparisons were performed using a two-sided Mann-Whitney test (*** p<0.001, ** p<0.01, * p<0.05). Source data

In this study, we investigated animals that were generating spontaneous and puff-induced behaviors, including walking and grooming. However, ANs likely also encode other behavioral states. This is hinted at by the fact that some ANs’ neural activities were not well explained by any of our behavioral regressors, and nearly one-third of the ANs that we examined were unresponsive, possibly due to the absence of appropriate context. For example, we found that some silent ANs could become very active during leg movements *only* when the spherical treadmill was removed (SS51017 and SS38631) (Extended Data Fig. 10). In the future, it would be of great importance to obtain an even larger sampling of ANs in multiple behavioral contexts and to test the degree to which AN encoding is genetically hardwired or capable of adapting during motor learning or after injury^59,60^. Our finding that ANs encode behavioral states and convey these signals to integrative sensory and action selection centers in the brain may guide the study of ANs in the mammalian spinal cord^17,18,49^ and also accelerate the development of more effective bioinspired algorithms for robotic sensory contextualization and action selection^2^.

## Methods

### Fly stocks and husbandry

Split-Gal4 (spGal4) lines (SS*****) were generated by the Dickson laboratory and the FlyLight project (Janelia Research Campus). When generating split-Gal4 driver lines, we first annotated as many ANs as possible in the Gal4 MCFO image library. Then, we selected neurons based on their innervation patterns within the VNC (that is, disregarding brain innervation patterns and genetic background information). We mainly targeted ANs with major innervation in the ventral part of VNC (that is, leg neuropils: VAC and intermediate neuropils for ProNm/MesoNm/MetaNm) as well as the lower and intermediate regions of the tectulum. We did not include ANs with major innervations of the wing/haltere tectulum and abdominal ganglia. We also did not include putative neuromodulator ANs with large cell bodies in the midline of VNC and characteristic innervation patterns (for example, spreading throughout the VNC or having no branching within the VNC).

GMR lines, MCFO-5 (R57C10-Flp2::PEST in su(Hw)attP8; ; HA-V5-FLAG), MCFO-7 (R57C10-Flp2::PEST in attP18;;HA-V5-FLAG-OLLAS)^27^ and UAS-syt:GFP (Pw[+mC]=UAS-syt.eGFP1, w[*]; ;) were obtained from the Bloomington Stock Center. MAN-spGal4f(; VT50660-AD; VT14014-DBD) and UAS-OpGCaM6f; UAS-tdTomato (; P20XUAS-IVS-Syn21-OpGCamp6F-p10 su(Hw)attp5; Pw[+mC]=UAS-tdTom.S3) were gifts from the Dickinson laboratory (Caltech). UAS-smFP (; ; 10xUAS-IVS-myr::smGdP-FLAG (attP2)) was a gift from the McCabe laboratory (EPFL).

Experimental animals were kept on dextrose cornmeal food at 25 °C and 70% humidity on a 12-hour light/dark cycle using standard laboratory tools. All strains used are listed in Supplementary Table 1. Female flies were subjected to experimentation 3–6 days post eclosion (dpe). Crosses used for experiments were flipped every 2–3 days.

### Ethical compliance

All experiments were performed in compliance with relevant national (Switzerland) and institutional (EPFL) ethical regulations.

### In vivo two-photon calcium imaging experiments

Two-photon imaging was performed as described in ref. ^28^ with minor changes in the recording configuration. We used ThorImage 3.1 software to record coronal sections of AN axons in the cervical connective to avoid having neurons move outside the field of view due to behavior-related tissue deformations. Imaging was performed using a galvo-galvo scanning system. Image dimensions ranged from 256 × 192 pixels (4.3 fps) to 320 × 320 pixels (3.7 fps), depending on the location of axonal ROIs and the degree of displacement caused by animal behavior. During two-photon imaging, a seven-camera system was used to record fly behaviors as described in ref. ^29^. Rotations of the spherical treadmill and the timing of puff stimuli were also recorded. Air or CO_2_ puffs (0.08 L min^−1^) were controlled either using a custom Python script or manually with an Arduino controller. Puffs were delivered through a syringe needle positioned in front of the animal to stimulate behavior in sedentary animals or to interrupt ongoing behaviors. To synchronize signals acquired at different sampling rates—optic flow sensors, two-photon images, puff stimuli and videography—signals were digitized using a BNC 2110 terminal block (National Instrument) and saved using ThorSync 3.1 software (Thorlabs). Sampling pulses were then used as references to align data based on the onset of each pulse. Then, signals were interpolated using custom Python scripts.

### Immunofluorescence tissue staining and confocal imaging

Fly brains and VNCs from 3–6-dpe female flies were dissected and fixed as described in ref. ^28^ with small modifications in staining, including antibodies and incubation conditions (see details below). Both primary antibodies (rabbit anti-GFP at 1:500, Thermo Fisher Scientific, RRID: AB_2536526; mouse anti-Bruchpilot/nc82 at 1:20, Developmental Studies Hybridoma Bank, RRID: AB_2314866) and secondary antibodies (goat anti-rabbit secondary antibody conjugated with Alexa Fluor 488 at 1:500, Thermo Fisher Scientific, RRID: AB_143165; goat anti-mouse secondary antibody conjugated with Alexa Fluor 633 at 1:500, Thermo Fisher Scientific, RRID: AB_2535719) for smFP and nc82 staining were performed at room temperature for 24 h.

To perform high-magnification imaging of MCFO samples, nervous tissues were incubated with primary antibodies: rabbit anti-HA-tag at 1:300 dilution (Cell Signaling Technology, RRID: AB_1549585), rat anti-FLAG-tag at 1:150 dilution (DYKDDDDK, Novus, RRID: AB_1625981) and mouse anti-Bruchpilot/nc82 at 1:20 dilution. These were diluted in 5% normal goat serum in PBS with 1% Triton-X (PBSTS) for 24 h at room temperature. The samples were then rinsed 2–3 times in PBS with 1% Triton-X (PBST) for 15 min before incubation with secondary antibodies: donkey anti-rabbit secondary antibody conjugated with Alexa Fluor 594 at 1:500 dilution (Jackson ImmunoResearch, RRID: AB_2340621), donkey anti-rat secondary antibody conjugated with Alexa Fluor 647 at 1:200 dilution (Jackson ImmunoResearch, RRID: AB_2340694) and donkey anti-mouse secondary antibody conjugated with Alexa Fluor 488 at 1:500 dilution (Jackson ImmunoResearch, RRID: AB_2341099). These were diluted in PBSTS for 24 h at room temperature. Again, samples were rinsed 2–3 times in PBS with PBST for 15 min before incubation with the last diluted antibody: rabbit anti-V5-tag (GKPIPNPLLGLDST) conjugated with DyLight 550 at 1:300 dilution (Cayman Chemical, 11261) for another 24 h at room temperature.

To analyze single-neuron morphological patterns, we crossed spGal4 lines with MCFO-7 (ref. ^27^). Dissections and MCFO staining were performed by Janelia FlyLight according to the FlyLight ‘IHC-MCFO’ protocol: https://www.janelia.org/project-team/flylight/protocols. Samples were imaged on an LSM 710 confocal microscope (Zeiss) with a Plan-Apochromat ×20/0.8 M27 objective.

To prepare samples expressing tdTomato and syt:GFP, we chose to stain only tdTomato to minimize false-positive signals for the synaptotagmin marker. Samples were incubated with a diluted primary antibody: rabbit polyclonal anti-DsRed at 1:1,000 dilution (Takara Biomedical Technology, RRID: AB_10013483) in PBSTS for 24 h at room temperature. After rinsing, samples were then incubated with a secondary antibody: donkey anti-rabbit secondary antibody conjugated with Cy3 at 1:500 dilution (Jackson ImmunoResearch, RRID: AB_2307443). Finally, all samples were rinsed two to three times for 10 min each in PBST after staining and then mounted onto glass slides with bridge coverslips in SlowFade mounting media (Thermo Fisher Scientific, S36936).

Confocal imaging was performed as described in ref. ^28^. In addition, high-resolution images for visualizing fine structures were captured using a ×40 oil-immersion objective lens with an NA of 1.3 (Plan-Apochromat ×40/1.3 DIC M27, Zeiss) on an LSM 700 confocal microscope (Zeiss). The zoom factor was adjusted based on the ROI size of each sample between 84.23 × 84.23 μm^2^ and 266.74 × 266.74 μm^2^. For high-resolution imaging, *z*-steps were fixed at 0.33 μm. Confocal images were acquired using Zen 2011 14.0 software. Images were denoised; their contrasts were tuned; and standard deviation *z*-projections were generated using Fiji version 2.9.0 (ref. ^61^).

### Two-photon image analysis

Raw two-photon imaging data were converted to grayscale TIFF image stacks for both green and red channels using custom Python scripts. RGB image stacks were then generated by combining both image stacks in Fiji (ref. ^61^). We used AxoID to perform ROI segmentation and to quantify fluorescence intensities. In brief, AxoID was used to register images using cross-correlation and optic-flow-based warping^28^. Then, raw and registered image stacks underwent ROI segmentation, allowing %Δ*F*/*F* values to be computed across time from absolute ROI pixel values. Simultaneously, segmented RGB image stacks overlaid with ROI contours were generated. Each frame of these segmented image stacks was visually examined to confirm AxoID segmentation or to perform manual corrections using the AxoID graphical user interface (GUI). In these cases, manually corrected %Δ*F*/*F* and segmented image stacks were updated. Our calculated value of 247 ANs is based on the number of ROIs observed in two-photon imaging data. However, we caution that each ROI may actually include closely intermingled fibers from several neurons.

### Behavioral data analysis

To reduce computational and data storage requirements, we recorded behaviors at 30 fps. This is nearly the Nyquist frequency for rapid walking (up to 16 step cycles per second^62^).

3D joint positions were estimated using DeepFly3D (ref. ^29^). Owing to the amount of data collected, manual curation was not practical. Therefore, we classified points as outliers when the absolute value of any of their coordinates (*x*, *y*, *z*) was greater than 5 mm (much larger than the fly’s body size). Furthermore, we made the assumption that joint locations would be incorrectly estimated for only one of the three cameras used for triangulation. The consistency of the location across cameras could be evaluated using the reprojection error. To identify a camera with a bad prediction, we calculated the reprojection error using only two of the three cameras. The outlier was then replaced with the triangulation result of the pair of cameras with the smallest reprojection error. The output was further processed and converted to angles as described in ref. ^57^.

We classified behaviors based on a combination of 3D joint angle dynamics and rotations of the spherical treadmill. First, to capture the temporal dynamics of joint angles, we calculated wavelet coefficients for each angle using 15 frequencies between 1 Hz and 15 Hz (refs. ^63,64^). We then trained a histogram gradient boosting classifier^65^ using joint angles, wavelet coefficients and ball rotations as features. Because flies perform behaviors in an unbalanced way (some behaviors are more frequent than others), we balanced our training data using SMOTE^66^. In brief, for less frequent behaviors, SMOTE upsamples the number of data points to match that of the most frequent behavior. To do this, it adds new data points through linear interpolation. Note that we only processed the training data in this way to get better classification accuracy for less common behaviors. The test data were not upsampled. Thus, we show a different number of frames in Extended Data Fig. 1e. The model was validated using five-fold, three-times-repeated, stratified cross-validation.

Fly speeds and heading directions were estimated using optical flow sensors^28^. To further improve the accuracy of the onset of walking, we applied empirically determined thresholds (pitch: 0.0038; roll: 0.0038; yaw: 0.014) to the rotational velocities of the spherical treadmill. The rotational velocities were smoothed and denoised using a moving average filter (length 81). All frames that were not previously classified as grooming or pushing, and for which the spherical treadmill was classified as moving, were labeled as ‘walking’. These were furthered subdivided into forward or backward walking depending on the sign of the pitch velocity. Conversely, frames for which the spherical treadmill was not moving were labeled as ‘resting’. To reduce the effect of optical flow measurement jitter, walking and resting labels were processed using a hysteresis filter that changes state only if at least 15 consecutive frames are in a new state. Classification in this manner was generally effective but most challenging for kinematically similar behaviors, such as eye and antennal grooming or hindleg rubbing and abdominal grooming (Extended Data Fig. 1e).

PE events were classified based on the length of the proboscis (Extended Data Fig. 1a–d). First, we trained a deep network^39^ to identify the tip of the proboscis and a static landmark (the ventral part of the eye) from side-view camera images. Then, the distance between the tip of proboscis and this static landmark was calculated to obtain the PE length for each frame. A semi-automated PE event classifier was made by first denoising the traces of PE distances using a median filter with a 0.3-s running average. Traces were then normalized to be between 0 (baseline values) and 1 (maximum values). Next, PE speed was calculated using a data point interval of 0.1 s to detect large changes in PE length. This way, only peaks larger than a manually set threshold of 0.03 upon Δnormalized length per 0.1 s were considered. Because the peak speed usually occurred during the rising phase of a PE, a kink in PE speed was identified by multiplying the peak speed with an empirically determined factor ranging from 0.4 to 0.6 and finding that speed within 0.5 s before the peak speed. The end of a PE was the timepoint at which the same speed was observed within 2 s after the peak PE speed. This filtered out occasions where the proboscis remained extended for long periods of time. All quantified PE lengths and durations were then used to build a filter to remove false positives. PEs were then binarized to define PE epochs.

To quantify animal movements when the spherical treadmill was removed, we manually thresholded the variance of pixel values from a side-view camera within a region of the image that included the fly. Pixel value changes were calculated using a running window of 0.2 seconds. Next, the standard deviation of pixel value changes was generated using a running window of 0.25 seconds. This trace was then smoothed, and values lower than the empirically determined threshold were called ‘resting’ epochs. The remainder were considered ‘movement’ periods.

### Regression analysis of PE integration time

To investigate the integrative nature of the PE-AN responses, we convolved PE traces with uniform time windows of varying sizes. This convolution was performed such that the fluorescence at each timepoint would be the sum of fluorescence during the previous ‘window_size’ frames (that is, not a *centered* sliding window but one that uses only previous timepoints), effectively integrating over the number of previous PEs. This integrated signal was then masked such that all timepoints where the fly was not engaged in PE were set to zero. Then, this trace was convolved with a calcium indicator decay kernel, notably yielding non-zero values in the time intervals between PEs. We then determined the explained variance as described elsewhere and finally chose a window size maximizing the explained variance.

### Linear modeling of neural fluorescence traces

Each regression matrix contains elements corresponding to the results of a ridge regression model for predicting the time-varying fluorescence ($\% \frac{{{\Delta }}F}{F}$) of ANs using specific regressors (for example, joint angles or behaviors). To account for slow calcium indicator decay dynamics, each regressor was convolved with a calcium response function. The half-life of the calcium response function was chosen from a range of 0.2 s to 0.95 s (ref. ^50^) in 0.05-s steps to maximize the variance in fluorescence traces that convolved regressors could explain. The rise time was fixed at 0.1415 seconds^50^. The ridge penalty parameter was chosen using nested ten-fold stratified cross-validation^67^. The intercept and weights of all models examining behavioral regressors were restricted to be positive, limiting our analysis to excitatory neural activity (this was not the case for models examining joint angle encoding, which could be either positive or negative). This constraint was required to study the UEV of behavioral regressors. For example, otherwise the variance of a walk-encoding AN could be nearly equally well explained by a positive walking regressor as by a negative resting regressor. Although our approach to %Δ*F*/*F* baseline normalization confounds the search for negative (putative inhibitory) deflections, our thorough visual inspection of neural activity traces did not reveal bi-phasic deflections from baseline. These would be expected if ANs were excited or inhibited depending on the ongoing behavioral state. Values shown in the matrices are the mean of ten-fold stratified cross-validation. We calculated UEV and all-explained variance (AEV) by temporally shuffling the regressor in question or all other regressors, respectively^4^. We tested the overall significance of our models using an *F*-statistic to reject the null hypothesis that the model does not perform better than an intercept-only model. The prediction of individual traces was performed using a single regressor plus intercept. Therefore, they were not regularized.

### Behavior-based neural activity analysis

For a given behavior, Δ*F*/*F* traces were compiled, cropped and aligned with respect to their onset times. Mean and 95% confidence intervals for each timepoint were then calculated from these data. Because the duration of each behavioral epoch was different, we computed mean and confidence intervals only for epochs that had at least five data points.

To test if each behavior-triggered average Δ*F*/*F* was significantly different from the baseline, first, we aligned and upsampled fluorescence data that were normalized between 0 (baseline mean) and 1 (maximum) for each trial. For each behavioral epoch, the first 0.7 s of data were removed. This avoided contaminating signals with neural activity from preceding behaviors (due to the slow decay dynamics of OpGCaMP6f). Next, to be conservative in judging whether data reflected noisy baseline or real signals, we studied their distributions. Specifically, we tested the normality of 20 resampled groups of 150 bootstrapped data points—a size that reportedly maximizes the power of the Shapiro–Wilk test^68^. If a majority of results did not reject the null hypothesis, the entire recording was considered baseline noise, and the Δ*F*/*F* for a given behavioral class was not considered significantly different from baseline. On the other hand, if the data points were not normally distributed, the baseline was determined using an Otsu filter. For recordings that passed this test of normality, if the majority of six ANOVA tests on the bootstrapped data rejected the null hypothesis, and the data points of a given behavior were significantly different (***P < 0.001, **P < 0.01, *P < 0.05) from baseline (as indicated by a post hoc Tukey test), these data were considered signal and not noise.

To analyze PE-AN responses to each PE during PE trains, putative trains of PEs were manually identified to exclude discrete PE events. PE trains included at least three consecutive PEs in which each PE lasted at least 1 second, and there was less than 3 s between each PE. Then, the mean fluorescence of each PE was computed for 25 PE trains (*n* = 11 animals). The median, interquartile range (IQR) and 1.5× IQR were then computed for PEs depending on their ordered position within their PE trains. We focused our analysis on the first 11 PEs because they had a sufficiently large amount of data.

### Neural fluorescence-triggered averages of spherical treadmill rotational velocities

A semi-automated neural fluorescence event classifier was constructed by first denoising Δ*F*/*F* traces by averaging them using a 0.6-s running window. Traces were then normalized to be between 0 (their baseline values) and 1 (their maximum values). To detect large deviations, the derivative of the normalized Δ*F*/*F* time series was calculated at an interval of 0.1 seconds. Only peaks greater than an empirically determined threshold of 0.03 upon Δnormalized Δ*F*/*F* per 0.1 s were considered events. Because peak fluorescence derivatives occurred during the rising phase of neural fluorescence events, the onset of a fluorescence event was identified as the time where the Δ*F*/*F* derivative was 0.4–0.6× the peak within the preceding 0.5-s time window. The end of the event was defined as the time that the Δ*F*/*F* signal returned to the amplitude at event onset before the next event. False positives were removed by filtering out events with amplitudes and durations that were lower than the empirically determined threshold. Neural activity event analysis for turn ANs was performed by testing if the mean normalized fluorescence event for one ROI was larger than the other ROI by an empirically determined factor of 0.2×. Corresponding ball rotations for events that pass these criteria were then collected. Fluorescence events onsets were then set to 0 s and aligned with spherical treadmill rotations. Using these rotational velocity data, we calculated the mean and 95% confidence intervals for each timepoint with at least five data points. A 1-s period before each fluorescence event was also analyzed as a baseline for comparison.

### Brain and VNC confocal image registration

All confocal images, except for MCFO image stacks, were registered based on nc82 neuropil staining. We built a template and registered images using the CMTK munger extension^69^. Code for this registration process can be found at https://github.com/NeLy-EPFL/MakeAverageBrain/tree/workstation. Brain and VNC of MCFO images were registered to JRC 2018 templates^70^ using the Computational Morphometry Toolkit: https://www.nitrc.org/projects/cmtk. The template brain and VNC can be downloaded here: https://www.janelia.org/open-science/jrc-2018-brain-templates.

### Analysis of individual AN innervation patterns

Single AN morphologies were traced by masking MCFO confocal images using either active tracing or manual background removal in Fiji^61^. Axons in the brain were manually traced using the Fiji plugin ‘SNT’. Most neurites in the VNC were isolated by (1) thresholding to remove background noise and outliers and (2) manually masking debris in images. In the case of ANs from SS29579, a band-pass color filter was applied to isolate an ROI that spanned across two color channels. The boundary of the color filter was manually tuned to acquire the stack for a single-neuron mask. After segmentation, the masks of individual neurons were applied across frames to calculate the intersectional pixel-wise sum with another mask containing (1) neuropil regions of the brain and VNC, (2) VNC segments or (3) left and right halves of the VNC. Brain and VNC neuropil regions and their corresponding abbreviations were according to established nomenclature^71^. Neuropil region masks can be downloaded here: https://v2.virtualflybrain.org. These were also registered to the JRC 2018 template. Masks for T1, T2 and T3 VNC segments were based on previously delimited boundaries^38^. The laterality of a neuron’s VNC innervation was calculated as the ratio of the absolute difference between its left and right VNC innervations divided by its total innervation. The bilaterality index is thus 1-laterality. Masks for the left and right VNC were generated by dividing the VNC mask across its midline.

### Statistics and reproducibility

This study was designed as a functional and anatomical screen of many *Drosophila* driver lines. Each line was functionally examined in 2–5 animals each. Anatomical studies were very reliable across samples. AN encodings were qualitatively reliable for the same driver line across animals aside from differences in SNR as well as minor variability in the number of ROIs for a subset of driver lines. No statistical methods were used to predetermine sample sizes. Our sample sizes are justified by AN functional response reliability and the long time required to functionally screen 70 driver lines in behaving animals. Experimental flies were excluded from functional analysis if two-photon microscopy data had a low SNR or occlusions or if animals appeared unhealthy after dissection. Because we performed a functional screen without prior hypotheses, the experiments were not randomized, and data collection and analyses were not performed blinded to the conditions of the experiments. To avoid false-positives due to statistical comparisons across a large numbers of tests, the data were bootstrapped (10 groups with sample size 30) and the majority of results for multiple Mann–Whitney *U*-tests determined whether or not to reject the null hypothesis. For the analysis of normalized mean Δ*F*/*F* responses, for a given AN across all epochs of a specific behavior, the data distribution was assumed to be normal, but this was not formally tested. Otherwise, statistical analyses were non-parametric.

### AxoID: a deep-learning-based software for tracking axons in imaging data

AxoID aims to extract the GCaMP fluorescence values for axons present on coronal section two-photon microscopy imaging data. In this manuscript, it is used to record activity from ANs passing through the *D. melanogaster* cervical connective. Fluorescence extraction works by performing the following three main steps (Extended Data Fig. 2). First, during a *detection* stage, ROIs corresponding to axons are segmented from images. Second, during a *tracking* stage, these ROIs are tracked across frames. Third, *fluorescence* is computed for each axon over time.

To track axons, we used a two-step approach: detection and then tracking. This allowed us to improve each problem separately without the added complexity of developing a detector that must also do tracking. Additionally, this allowed us to detect axons without having to know how many there are in advance. Finally, substantial movement artifacts between consecutive frames pose additional challenges for robustness in temporal approaches, although, in our case, we can apply the detection on a frame-by-frame basis. However, we note that we do not leverage temporal information.

#### Detection

Axon detection consists of finding potential axons by segmenting the background and foreground of each image. An ROI or putative axon is defined as a group of connected pixels segmented as foreground. Pixels are considered connected if they are next to one another.

By posing detection as a segmentation problem, we have the advantage of using standard computer vision methods, such as thresholding or artificial neural networks, that have been developed for medical image segmentation. Nevertheless, this simplicity has a drawback: if axons appear very close to one another and their pixels are connected, they may be segmented as one ROI rather than two. We try to address this issue using an ROI separation approach described later.

Image segmentation is performed using deep learning on a frame-by-frame basis, whereby a network generates a binary segmentation of a single image. As a post-processing step, all ROIs smaller than a minimum size are discarded. Here, we empirically chose 11 pixels as the minimum size as a tradeoff between removing small spurious regions while still detecting small axons.

We chose to use a U-Net model^72^ with slight modifications because of its, or its derivatives’, performance on recent biomedical image segmentation problems^73–75^. We add zero-padding to the convolutions to ensure that the output segmentation has the same size as the input image, thus fully segmenting it in a single pass, and modify the last convolution to output a single channel rather than two. Batch normalization^76^ is used after each convolution and its non-linearity function. Finally, we reduce the width of the network by a factor of 4: each feature map has four times fewer channels than the original U-Net, not counting the input or output. The input pixel values are normalized to the range [−1, 1], and the images are sufficiently zero-padded to ensure that the size can be correctly reduced by half at each max-pooling layer.

To train the deep learning network, we use the Adam optimizer^77^ on the binary cross-entropy loss with weighting. Each background pixel is weighted based on its distance to the closest ROI, given by $1+exp(-{\frac{d}{3}}^{2})$ with *d* as the Euclidean distance, plus a term that increases if the pixel is a border between two axons, given by $exp(-{\frac{{d}_{1}+{d}_{2}}{6}}^{2})$, with *d*_1_ and *d*_2_ as the distances to the two closest ROIs, as in ref. ^72^. These weights aim to encourage the network to correctly segment the border of the ROI and to keep a clear separation between two neighboring regions. At training time, the background and foreground weights are scaled by $\frac{b+f}{2b}$ and $\frac{b+f}{2f}$, respectively, to take into account the imbalance in the number of pixels, where *b* and *f* are the quantity of background and foreground (that is, ROI) pixels in the entire training dataset. To evaluate the resulting deep network, we use the Sørensen–Dice coefficient^78,79^ at the pixel level, which is equivalent to the F1 score. The training is stopped when the validation performance does not increase anymore.

The network was trained on a mix of experimental and synthetic data. We also apply random gamma corrections to the training input images, with *γ* sampled in [0.7, 1.3] to keep reasonable values and to encourage robustness against intensity variations between experiments. The target segmentation of the axons on the experimental data was generated with conventional computer vision methods. First, the images were denoised with the non-local means algorithm^80^ using the Python implementation of OpenCV^81^. We used a temporal window size of 5 and performed the denoising separately for the red and green channels, with a filter strength *h* = 11. The grayscale result was then taken as the per-pixel maximum over the channels. After this, the images were smoothed with a Gaussian kernel of standard deviation 2 pixels and thresholded using the Otsu method^82^. A final erosion was applied, and small regions below 11 pixels were removed. All parameter values were set empirically to generate good qualitative results. In the end, the results were manually filtered to keep only data with satisfactory segmentation.

Because the experimental data have a fairly simply visual structure, we constructed a pipeline in Python to generate synthetic images visually similar to real ones. This was achieved by first sampling an image size for a given synthetic experiment and then by sampling 2D Gaussians over it to simulate the position and shape of axon cross-sections. After this, synthetic tdTomato levels were uniformly sampled, and GCaMP dynamics were created for each axon by convolving a GCaMP response kernel with Poisson noise to simulate spikes. Then, the image with the Gaussian axons was deformed multiple times to make different frames with artificial movement artifacts. Eventually, we sampled from the 2D Gaussians to make the axons appear pixelated and added synthetic noise to the images.

In the end, we chose a deep-learning-based approach because our computer vision pipeline alone was not be robust enough. Our pipeline is used to generate a target segmentation dataset from which we manually select a subset of acceptable results. These results are then used to train the deep learning model.

Fine-tuning. At the beginning of the detection stage, an optional fine-tuning of the network can be applied to try to improve the segmentation of axons. The goal is to have a temporary network adapted to the current data for better performance. To do this, we train the network on a subset of experimental frames using automatically generated target segmentations.

The subset of images is selected by finding a cluster of frames with high cross-correlation-based similarity. For this, we consider only the tdTomato channel to avoid the effects of GCaMP dynamics. Each image is first normalized by its own mean pixel intensity *μ* and standard deviation *σ*: $p(i,j)\leftarrow \frac{p(i,j)-\mu }{\sigma }$, where *p*(*i*, *j*) is the pixel intensity *p* at the pixel location *i*, *j*. The cross-correlation is then computed between each pair of normalized images *p*_*m*_ and *p*_*n*_ as ∑_*i*,*j*_*p*_*m*_(*i*, *j*) ⋅ *p*_*n*_(*i*, *j*). Afterwards, we take the opposite of the cross-correlation as a distance measure and use it to cluster the frames with the OPTICS algorithm^83^. We set the minimal number of samples for a cluster to 20 to maintain at least 20 frames for fine-tuning and a maximum neighborhood distance of half the largest distance between frames. Finally, we select the cluster of images with the highest average cross-correlation (that is, the smallest average distance between its elements).

Then, to generate a target segmentation image for these frames, we take their temporal average and optionally smooth it, if there are fewer than 50 images, to help remove the noise. The smoothing is done by filtering with a Gaussian kernel of standard deviation 1 pixel and then median filtering over each channel separately. The result is then thresholded through a local adaptive method, computed by taking the weighted mean of the local neighborhood of the pixel, subtracted by an offset. We apply Gaussian weighting over windows of 25 × 25 pixels, with an offset of −0.05, determined empirically. Finally, we remove regions smaller than 11 pixels. The result serves as a target segmentation image for all of the fine-tuning images.

The model is then trained on 60% of these frames with some data augmentation, whereas the other 40% are used for validation. The fine-tuning stops automatically if the performance on the validation frames drops. This avoids bad generalization for the rest of the images. The binary cross-entropy loss is used, with weights computed as discussed previously. For the data augmentation, we use random translation (±20%), rotation (±10°), scaling (±10%) and shearing (±5°).

#### Tracking

Once the ROIs are segmented, the next step of the pipeline consists of tracking the axons through time. This means defining which axons exist and then finding the ROI they correspond to in each frame.

Tracking template. To accomplish this, the tracker records the number of axons, their locations with respect to one another and their areas. It stores this information into what we call the ‘tracker template’. Then, for each frame, the tracker matches its template axons to the ROIs to determine which regions correspond to which axons.

The tracker template is built iteratively. It is first initialized and then updated by matching with all experimental data. The initialization depends on the optional fine-tuning in the detection step. If there is fine-tuning, then the smoothed average of the similar frames and its generated segmentation are used. Otherwise, one frame of the experiment is automatically selected. For this, AxoID considers only the frames with a number of ROIs equal to the most frequent number of ROIs and then selects the image with the highest cross-correlation with the temporal average of these frames. It is then smoothed and taken with the segmentation produced by the detection network as initialization. The cross-correlation and smoothing are computed identically as in the fine-tuning. Each ROI in the initialization segmentation defines an axon in the tracker template, with its area and position recorded as initial properties.

Afterwards, we update them by matching each experimental frame to the tracker template. It consists of assigning the ROI to the tracker axons and then using these regions’ areas and positions to update the tracker. The images are matched sequentially, and the axons properties are taken as running averages of their matched regions. For example, considering the *n*th match, the area of an axon is updated as:

$${\mathrm{area}}\leftarrow \frac{{\mathrm{area}} \times n+{\mathrm{area}}_{{\mathrm{ROI}}}}{n+1}$$

Because of this, the last frames are matched to a tracker template that is different from the one used for the first frames. Therefore, we fix the axon properties after the updates and match each frame again to obtain the final identities of the ROIs.

Matching. To assign axon identities to the ROIs of a frame, we perform a matching between them as discussed above. To solve it, we define a cost function for matching a template axon to a region that represents how dissimilar they are. Then, using the Hungarian assignment algorithm^84^, we find the optimal matching with the minimum total cost (Extended Data Fig. 2b).

Because some ROIs in the frame may be wrong detections, or some axons may not be correctly detected, the matching has to allow for the regions and axons to end up unmatched for some frames. Practically, we implement this by adding ‘dummy’ axons to the matching problem with a flat cost. To guarantee at least one real match, the flat cost is set to the maximum between a fixed value and the minimum of the costs between regions and template axons with a margin of 10%: dummy = max(*v*, 1.1 ⋅ min(costs)) with *v* = 0.3 the fixed value. Then, we can use the Hungarian method to solve the assignment, and all ROIs linked to these dummy axons can be considered unmatched.

We define the cost of assigning a frame’s ROI *i* to a tracker template axon *k* by their absolute difference in area plus the mean cost of an optimal inner matching of the other ROI to the other axons, assuming *i* and *k* are already matched:

$${{{{{\mathrm{cost}}}}}}(i,k)={w}_{{\mathrm{area}}}| {\mathrm{are{a}}}_{i}-{\mathrm{are{a}}}_{k}| +\frac{1}{{N}_{\mathrm{{ROI}}}-1}\mathop{\sum}\limits_{{i}^{{\prime}}\ne i}{{{{\rm{cost}}}}}^{{\prime}}({i}^{{\prime}},{k}_{{i}^{{\prime}}}^{*})$$

where *w*_area_ = 0.1 is a weight for balancing the importance of the area; *N*_ROI_ is the number of ROIs in the frame; and ${{{{\rm{cost}}}}}^{{\prime} }({i}^{{\prime} },{k}_{{i}^{{\prime} }}^{* })$ is the inner cost of assigning region ${i}^{{\prime} }\ne i$ to axon ${k}_{{i}^{{\prime} }}^{* }\ne k$ selected in an ‘inner’ assignment problem—see below. In other words, the cost is relative to how well the rest of the regions and axons match if we assume that *i* and *k* are already matched.

The optimal inner matching is computed through another Hungarian assignment, for which we define another cost function. For this ‘inner’ assignment problem, the cost of matching an ROI ${i}^{{\prime} }\ne i$ and a template axon ${k}^{{\prime} }\ne k$ is defined by how far they are and their radial difference with respect to the matched *i* and *k*, plus their difference in area:

$$\begin{array}{l}{{{{\rm{cost}}}}}^{{\prime} }({i}^{{\prime} },{k}^{{\prime} })=\left(\frac{{w}_{{\rm{dist}}}}{{\eta }_{{\rm{dist}}}}| | ({x}_{{i}^{{\prime} }}-{x}_{i})-({x}_{{k}^{{\prime} }}-{x}_{k})| | +\frac{{w}_{\theta }}{{\eta }_{\theta }}| {\theta }_{{i}^{{\prime} }}-{\theta }_{{k}^{{\prime} }}| \right)\\\frac{H}{H+{x}_{{k}^{{\prime} }}^{y}}+{w}_{{\rm{area}}}| {\rm{are{a}}}_{{i}^{{\prime} }}-{\rm{are{a}}}_{{k}^{{\prime} }}|\end{array}$$

$${{{\rm{with}}}}\,\,{\eta }_{\theta }=\arctan \left({\alpha }_{\theta }\frac{{\eta }_{dist}}{| | {x}_{{k}^{{\prime} }}-{x}_{k}| | }\right)$$

where *w*_dist_ = 1.0, *w*_*θ*_ = 0.1 and *w*_area_ = 0.1 are weighting parameters; ${\eta }_{{\rm{dist}}}=\min (H,W)$ and *η*_*θ*_ are normalization factors with *H* and *W* as the height and width of the frame; and *α*_*θ*_ = 0.1 is a secondary normalization factor. The ⋅^*y*^ operation returns the height component of a vector, and the $\frac{H}{H+{x}_{{k}^{{\prime} }}^{y}}$ term is useful to reduce the importance of the first terms if the axon ${k}^{{\prime} }$ is far from axon *k* in the height direction. This is needed as the scanning of the animal’s cervical connective is done from top to bottom; thus, we need to allow for some movement artifacts between the top and bottom of the image. Note that the dummy axons for unmatched regions are also added to this inner problem.

This inner assignment is solved for each possible pair of axon–ROI to get all final costs. The overall matching is then performed with them. Because we are embedding assignments, the computational cost of the tracker increases exponentially with the number of ROIs and axons. It stays tractable in our case as we generally deal with few axons at a time. All parameter values used in the matching were found empirically by trial and error.

Identities post-processing. ROI separation: In the case of fine-tuning at the detection stage, AxoID will also automatically try to divide ROIs that are potentially two or more separate axons. We implement this to address the limitation introduced by detecting axons as a segmentation: close or touching axons may get segmented together.

To do this, it first searches for potential ROIs to be separated by reusing the temporal average of the similar frames used for the fine-tuning. This image is initially segmented as described before. Then, local intensity maxima are detected on a grayscale version of this image. To avoid small maxima due to noise, we keep only those with an intensity ≥0.05, assuming normalized grayscale values in [0, 1]. After this, we use the watershed algorithm, with the scikit-image^85^ implementation, to segment the ROI based on the gray level and detected maxima. In the previous stages, we discarded ROIs under 11 pixels to avoid small spurious detections. Similarly, here we fuse together adjacent regions that are under 11 pixels to output results only after the watershedding above or equal to that size. Finally, a border of 1 pixel width is inserted between regions created from the separation of an ROI.

These borders are the divisions separating the ROI, referred to as ‘cuts’. We parameterize each of these as a line, defined as its normal vector **n** and distance *d* to the origin of the image (top left). To report them on each frame, we first normalize this line to the current ROI and then reverse that process with respect to the corresponding regions on the other frames. To normalize the line to an ROI, we fit an ellipse on the ROI contour in a least-square sense. Then, the line parameters are transformed into this ellipse’s local coordinates following Algorithm 1. It is essentially like transforming the ellipse into a unit circle, centered and axis-aligned, and applying a similar transformation to the cutting line (Extended Data Fig. 2c, middle). The choice of fitting an ellipse is motivated by the visual aspect of the axons in the experimental data as they are fairly similar to elongated ellipses. Considering this, a separation between two close ellipses could be simplified to a linear border, motivating the linear representation of the ROI separation.

Because this is done as a post-processing step after tracking, we can apply that division on all frames. To do this, we again fit an ellipse to their ROI contours in the least-squares sense. Then, we take the normalized cutting line and fit it back to each of them according to Algorithm 2. This is similar to transforming the normalized unit circle to the region ellipse and applying the same transform to the line (Extended Data Fig. 2c, right).

Finally, a new axon is defined for each cut. In each frame, the pixels of the divided region on the furthest side of the linear separation (with respect to the fitting ellipse center) are taken as the new ROI of that axon for that given frame.

In case there are multiple cuts of the same ROI (for example, because three axons were close), the linear separations are ordered by distance to the center of the fitting ellipse and are then applied in succession. This is simple and efficient but assumes there is little to no crossing between linear cuts.

Fluorescence extraction. With the detection and tracking results, we know where each axon is in the experimental data. Therefore, to compute tdTomato and GCaMP fluorophore time series, we take the average of non-zero pixel intensities of the corresponding regions in each frame. We report the GCaMP fluorescence at time *t* as *F*_*t*_ and the ratio of GCaMP to tdTomato fluorescence at time *t* as *R*_*t*_ to gain robustness against image intensity variations.

**Algorithm 1:** Normalize a line with an ellipse.

**Input**: *line*, *ellipse*

**Output**: normalized line *line*’

/* Initialization */

**n** ← *line*. **normal**;

*d* ← *line*. *distance*;

**c** ← *ellipse*. **center**;

*w* ← *ellipse*. *width*/2;

*h* ← *ellipse*. *height*/2;

*θ* ← *ellipse*. *rotation*;

**R**_−*θ*_: = rotation matrix of angle − *θ*;

/* Normalization */

$${d}^{{\prime} }\leftarrow d-{{{\bf{c}}}}\cdot {{{\bf{n}}}}$$;

$${{{{\bf{n}}}}}^{{\prime} }\leftarrow {{{{\bf{R}}}}}_{-\theta }\,{{{\bf{n}}}}$$;

$${{{{\bf{n}}}}}^{{\prime} }.x\leftarrow {{{{\bf{n}}}}}^{{\prime} }.x/{{{\bf{c}}}}.y$$;

$${{{{\bf{n}}}}}^{{\prime} }.y\leftarrow {{{{\bf{n}}}}}^{{\prime} }.y/{{{\bf{c}}}}.x$$;

$${d}^{{\prime} }\leftarrow {d}^{{\prime} }/(w* h)$$;

$$lin{e}^{{\prime} }.distance\leftarrow {d}^{{\prime} }/| | {{{{\bf{n}}}}}^{{\prime} }| |$$;

$$lin{e}^{{\prime} }.{{{\bf{normal}}}}\leftarrow {{{{\bf{n}}}}}^{{\prime} }/| | {{{{\bf{n}}}}}^{{\prime} }| |$$;

**Algorithm 2:** Fit a line to an ellipse

**Input**: *line*, *ellipse*

**Output**: fitted line *line*’

/* Initialization */

**n** ← *line*. **normal**;

*d* ← *line*. *distance*;

**c** ← *ellipse*. **center**;

*w* ← *ellipse*. *width*/2;

*h* ← *ellipse*. *height*/2;

*θ* ← *ellipse*. *rotation*;

**R**_*θ*_: = rotation matrix of angle *θ*;

/* Fitting */

$${{{{\bf{n}}}}}^{{\prime} }\leftarrow {{{\bf{n}}}}$$;

$${{{{\bf{n}}}}}^{{\prime} }.x\leftarrow {{{{\bf{n}}}}}^{{\prime} }.x* {{{\bf{c}}}}.y$$;

$${{{{\bf{n}}}}}^{{\prime} }.y\leftarrow {{{{\bf{n}}}}}^{{\prime} }.y* {{{\bf{c}}}}.x$$;

$${d}^{{\prime} }\leftarrow d* (w* h)$$;

$${d}^{{\prime} }\leftarrow {d}^{{\prime} }/| | {{{{\bf{n}}}}}^{{\prime} }| |$$;

$${{{{\bf{n}}}}}^{{\prime} }\leftarrow {{{{\bf{n}}}}}^{{\prime} }/| | {{{{\bf{n}}}}}^{{\prime} }| |$$;

$$lin{e}^{{\prime} }.{{{\bf{normal}}}}\leftarrow {R}_{\theta }\,{{{{\bf{n}}}}}^{{\prime} }$$;

$$lin{e}^{{\prime} }.distance\leftarrow {d}^{{\prime} }+{{{\bf{c}}}}\cdot {{{{\bf{n}}}}}^{{\prime} }$$;

The final GCaMP fluorescence is reported as in ref. ^28^:

$${{\Delta }}F/F=\frac{{F}_{t}-F}{F}$$

where *F* is a baseline fluorescence. Similarly, we report the ratio of GCaMP over tdTomato as in refs. ^28,86^:

$${{\Delta }}R/R=\frac{{R}_{t}-R}{R}$$

where *R* is the baseline. The baseline fluorescences *F* and *R* are computed as the minimal temporal average over windows of 10 s of the fluorophore time series *F*_*t*_ and *R*_*t*_, respectively. Note that axons can be missing in some frames—for instance, if they were not detected or leave the image during movement artifacts. In this case, the fluorescence of that axon will have missing values at the time index *t* in which it was absent.

#### Overall workflow

To improve the performance of AxoID, the fluorescence extraction pipeline is applied three times: once over the raw data, once over the data registered using cross-correlation and once over the data registered using optic flow warping. Note that the fine-tuning in the detection stage is not used with the raw experimental data as it is based on the cross-correlation between the frames and would, therefore, lead to worse or redundant results with the data registered using cross-correlation. Eventually, the three fluorescence results can be visualized, chosen from and corrected by a user through a GUI (Extended Data Fig. 2d).

Data registration. Registration of the experimental frames consists in transforming each image to make them similar to a reference image. The goal is to reduce the artifacts introduced by animal movements and to align axons across frames. This should help to improve the results of the detection and tracking.

Cross-correlation. Cross-correlation registration consists of translating an image so that its correlation to a reference is maximized. Note that the translated image wraps around (for example, pixels disappearing to the left reappear on the right). This aims to align frames against translations but is unable to counter rotations or local deformations. We used the single-step Discrete Fourier Transform (DFT) algorithm^87^ to find the optimal translation of the frame. It first transforms the images into the Fourier domain, computes an initial estimate of the optimal translation and then refines this result using a DFT. We based our Python implementation on previous work^88^.

For each experiment, the second frame is taken as the reference frame to avoid recording artifacts that sometimes appear on the first recorded image.

Optic flow registration. Optic-flow-based registration was previously published^28^. In brief, this approach computes an optic flow from the frame to a reference image and then deforms it by moving the pixels along that flow. The reference image is taken as the first frame of the experiment. This method has the advantage of being able to compute local deformations but at a high computational cost.

AxoID GUI. Finally, AxoID contains a GUI where a user can visualize the results, select the best one and manually correct it.

First, the user is presented with three outputs of the fluorescence extraction pipeline from the raw and registered data with the option of visualizing different information to select the one to keep and correct. Here, the detection and tracking outputs are shown as well as other information, such as the fluorescence traces in Δ*F*/*F* or Δ*R*/*R*. One of the results is then selected and used throughout the rest of the pipeline.

After this, the user can edit the tracker template, which will then automatically update the ROI identities across frames. The template and the identities for each frame are shown, with additional information, such as the image used to initialized the template. The user has access to different tools: axons can be fused, for example, if they actually correspond to a single real axon that was incorrectly detected as two, and, conversely, one axon can be manually separated in two if two close ones are detected together. Moreover, useless axons or wrong detections can be discarded.

Once the user is satisfied with the overall tracker, they can correct individual frames. At this stage, it is possible to edit the detection results by discarding, modifying or adding ROIs onto the selected image. Then, the user may change the tracking results by manually correcting the identities of these ROIs. In the end, the final fluorescence traces are computed on the selected outputs, including user corrections.

### Reporting Summary

Further information on research design is available in the Nature Portfolio Reporting Summary linked to this article.

## Online content

Any methods, additional references, Nature Portfolio reporting summaries, source data, extended data, supplementary information, acknowledgements, peer review information; details of author contributions and competing interests; and statements of data and code availability are available at 10.1038/s41593-023-01281-z.

## Supplementary information

Supplementary Information Supplementary Table 1. Sparse AN driver lines and associated properties. Supplementary videos (right-most column) for each driver line can be found here: https://dataverse.harvard.edu/dataverse/AN.

Reporting Summary

Supplementary Video 1 High-level behaviors, their associated 3D poses and spherical treadmill rotational velocities. Behaviors were captured from six camera views. Illuminated text (top) indicates the behavioral class being illustrated. Also shown are corresponding 3D poses (bottom left) and spherical treadmill rotational velocities, PE lengths and puff stimulation periods (bottom right).

Supplementary Video 2 Representative data for 50 comprehensively analyzed, AN-targeting sparse driver lines (see also Supporting Information pdf). Shown are: spFP staining (a), a representative two-photon microscope image (b), outline of the associated cervical connective after filling the surrounding bath with fluorescent dye (c) and PE length, puff stimuli, spherical treadmill rotational velocities and AN (ROI) Δ*F*/*F* traces (d). Indicated above are regressors for forward walking (‘F.W.’), backward walking (‘B.W.’), resting (‘Rest’), eye grooming (‘Eye groom’), antennal grooming (‘Ant. groom’), foreleg rubbing (‘Fl. rub’), abdominal grooming (‘Abd. groom’), hindleg rubbing (‘Hl. rub’) and proboscis extension (‘PE’). For each driver line, the title indicates ‘date-Gal4-reporters-fly#-trial#’.

Supplementary Data 1 Supplementary Data. Data for each examined driver line.

## Data Availability

Data are available at https://dataverse.harvard.edu/dataverse/AN. Owing to data storage limits, this does not include raw behavior camera images or raw two-photon imaging files. This repository includes synchronized neural fluorescence, behavior and ball rotational velocities; raw and traced MCFO confocal image data; neural data used for regression analyses, responses of PE-ANs and AN responses on and off of the spherical treadmill; behavioral data and the deep learning model for measuring proboscis extensions and annotations for training the behavior classifier; linear regression results; and a machine-readable version of Supplementary Table 1. For brain and VNC image registration, templates can be downloaded here: https://www.janelia.org/open-science/jrc-2018-brain-templates. Neuropil region masks can be downloaded here: https://v2.virtualflybrain.org. Source data are provided with this paper.

## Source data

Source Data Fig. 2 Raw data points for Fig. 2a–d.
Source Data Fig. 3 Raw data points for Fig. 3a,b.
Source Data Fig. 4 Raw data points for Fig. 4b,h.
Source Data Fig. 5 Raw data points for Fig. 5b,c,h,k,l.
Source Data Fig. 6 Raw data points for Fig. 6b,h.
Source Data Fig. 7 Raw data points for Fig. 7b.
Source Data Extended Data Fig. 3 Raw data points for Extended Data Fig. 3.
Source Data Extended Data Fig. 7 Raw data points for Extended Data Fig. 7c,d.
Source Data Extended Data Fig. 8 Raw data points for Extended Data Fig. 8g,j,m.
Source Data Extended Data Fig. 9 Raw data points for Extended Data Fig. 9a,b.
Source Data Extended Data Fig. 10 Raw data points for Extended Data Fig. 10a–d.

## Code availability

Analysis code is available at https://github.com/NeLy-EPFL/Ascending_neuron_screen_analysis_pipeline. AxoID code is available at https://github.com/NeLy-EPFL/AxoID.
