# Supplementary material for: Ascending neurons convey behavioral state to integrative sensory and action selection brain regions
Source: Nat Neurosci. 2023 Mar 23;26(4):682–95. doi: 10.1038/s41593-023-01281-z (PMC10076225; doi:10.1038/s41593-023-01281-z)
Supplement: Supplementary file 1 — Supplementary Table 1. Sparse AN driver lines and associated properties. Supplementary videos (right-most column) for each driver line can be found here: https://dataverse.harvard.edu/dataverse/AN. [file 41593_2023_1281_MOESM1_ESM.pdf]

nature neuroscience

Resource https://doi.org/10.1038/s41593-023-01281-z

Ascending neurons convey behavioral state to integrative sensory and action selection brain regions

In the format provided by the authors and unedited

Supplementary Table 1: **Sparse AN driver lines and associated properties.**
Supplementary videos (right-most column) for each driver line can be found here:
https://dataverse.harvard.edu/dataverse/AN

No.	GAL4	Confocal SNR of smFP	2P SNR of OpGCaMP6f and tdTomato	Number ROIs (representative fly, (other flies))	ROI# (left-right seperated by —)	Encoding (left-right seperated by —)	Level synchronous activity (ROI# vs ROI#: Corr. coef.)	Redundant GAL4	Supp. Video#
1	SS36131	strong	strong	2<br>(2,2)	0<br>—<br>1	rest<br>—<br>rest	0 vs 1 (pair): 0.7	SS36132<br>SS36133	3
2	SS38592	strong	strong	6<br>(5,4,3)	0<br>1<br>2<br>3<br>—<br>4<br>5	rest<br>rest<br>rest<br>unclear<br>—<br>unclear<br>rest	0 vs 5: 0.4<br>1 vs 5: 0.36<br>2 vs 5: 0.49	SS38598	4
3	SS27485	strong	strong	4<br>(6,2,2,4)	0<br>1<br>—<br>2<br>3	rest<br>unclear<br>—<br>rest<br>rest	0 vs 2 (pair): 0.85	-	5
4	SS41822	strong	strong	2<br>(3)	0<br>—<br>1	rest<br>—<br>rest	0 vs 1 (pair): 0.71	SS41808<br>SS41809<br>SS41820<br>SS41821	6
5	SS38624	strong	strong	4<br>(bifurcated)<br>(4,4)	0<br>1<br>—<br>2<br>3	rest<br>rest<br>—<br>rest<br>rest	0 vs 2 (pair): 0.84<br>0 vs 3 (pair): 0.7<br>1 vs 2 (pair): 0.9<br>1 vs 3 (pair): 0.87	-	7
6	SS45605	strong	strong	1<br>(1)	0	unclear	-	-	8
7	SS43652	strong	medium	5<br>(3,5)	0<br>1<br>2<br>—<br>3<br>4	rest<br>puff<br>rest<br>—<br>rest<br>rest	0 vs 3: 0.91<br>0 vs 4: 0.84<br>2 vs 3: 0.77<br>2 vs 4: 0.89	-	9
8	SS36112	strong	strong	2<br>(2,2)	0<br>—<br>1	puff<br>—<br>puff	0 vs 1 (pair): 0.51	-	10
9	SS41806	strong	strong	4<br>(3,4,4)	0<br>2<br>—<br>1<br>3	unclear<br>puff<br>—<br>unclear<br>puff	0 vs 1 (pair): 0.90<br>2 vs 3 (pair): 0.67	-	11
10	SS38631	strong	strong	2<br>(1)	0<br>—<br>1	off ball movement<br>—<br>off ball movement	0 vs 1 (pair): 0.88	-	12
11	SS51029	strong	medium	4<br>(4,3,4)	0<br>1<br>—<br>2<br>3	puff<br>puff<br>—<br>puff<br>puff	0 vs 2: 0.32<br>0 vs 3: 0.33<br>1 vs 2: 0.37<br>1 vs 3: 0.4	SS51024	13
12	R85A11	strong	strong	6<br>(6,6)	0<br>2<br>3<br>—<br>1<br>4<br>5	unresponsive<br>walk<br>unclear<br>—<br>unresponsive<br>walk<br>unclear	2 vs 4 (pair): 0.87	-	14
13	SS40489	-	strong	5<br>(5)	0<br>1<br>2<br>—<br>3<br>4	unclear<br>unclear<br>unclear<br>—<br>unclear<br>unclear	0 vs 3: 0.38<br>0 vs 4: 0.43<br>1 vs 3: 0.53<br>1 vs 4: 0.53	-	15
14	SS31480	strong	strong	3<br>(3)	0<br>1<br>2	walk<br>unclear<br>walk	0 vs 2: 0.61	-	16
15	SS51021	strong	strong	2<br>(2,2)	0<br>—<br>1	foreleg movement<br>—<br>foreleg movement	0 vs 1 (pair): 0.69	-	17
16	SS51017	strong	strong	2<br>(2,4,2)	0<br>—<br>1	off ball movement<br>—<br>off ball movement	0 vs 1 (pair): 0.67	-	18
17	SS31456	strong	strong	2<br>(2,2,2)	0<br>—<br>1	off ball movement<br>—<br>off ball movement	0 vs 1 (pair): 0.20	-	19
18	SS46233	strong	medium	2<br>(2,3,3,2)	0<br>—<br>1	walk<br>—<br>walk	0 vs 1 (pair): 0.63	-	20

Table 1 continued from previous page

No.	GAL4	Confocal SNR of smFP	2P SNR of OpGCaMP6f and tdTomato	Number ROIs (representative fly, (other flies))	ROI# (left-right seperated by —)	Encoding (left-right seperated by —)	Level synchronous activity (ROI# vs ROI#: Corr. coef.)	Redundant GAL4	Supp. Video#
19	SS42749	strong	strong	2<br>(2,2)	0<br>—<br>1	push<br>—<br>push	0 vs 1 (pair): 0.89	-	21
20	SS41815	strong	medium	2<br>(2,2,2)	0<br>—<br>1	unclear<br>—<br>unclear	0 vs 1 (pair): 0.42	-	22
21	SS29633	strong	strong	3<br>(3,2)	0<br>1<br>2	unclear<br>unclear<br>unclear	0 vs 1: 0.11<br>0 vs 2: 0.41<br>1 vs 2: 0.42	-	23
22	R87H02	strong	strong	2<br>(2,2,2)	0<br>—<br>1	unclear<br>—<br>unclear	0 vs 1 (pair): 0.48	-	24
23	MAN	strong	strong	2<br>(2,2,3,3,2)	0<br>—<br>1	push<br>—<br>push	0 vs 1 (pair): 0.82	-	25
24	SS49172	strong	strong	3<br>(2,5,3)	0<br>—<br>1<br>2	walk<br>—<br>walk<br>walk	0 vs 1: 0.54<br>0 vs 2: 0.53	-	26
25	R36G04	strong	strong	2<br>(4,3,3,3)	0<br>—<br>1	walk<br>—<br>walk	0 vs 1 (pair): 0.55	-	27
26	R39G01	strong	strong	3<br>(3,2,3,3)	0<br>1<br>2	unclear<br>unclear<br>unclear	0 vs 1: 0.09<br>0 vs 2: 0.82<br>1 vs 2: 0.19	-	28
27	SS31219	strong	strong	3<br>(4,3,3)	0<br>—<br>1<br>2	walk<br>walk<br>walk	0 vs 1: 0.67<br>0 vs 2: 0.74	-	29
28	R30A08	strong	medium	2<br>(1,2,2,1)	0<br>—<br>1	unclear<br>unclear	0 vs 1 (pair): 0.22	-	30
29	SS44270	strong	strong	3<br>(1,2)	0<br>—<br>1<br>2	walk<br>—<br>walk<br>unresponsive	0 vs 1 (pair): 0.98	SS41605	31
30	SS41605	strong	strong	4<br>(4,4)	0<br>2<br>—<br>1<br>3	unclear<br>push<br>—<br>unclear<br>push	0 vs 1 (pair): 0.90<br>2 vs 3 (pair): 0.94	SS44270	32
31	SS29579	strong	strong	6<br>(6,8)	0<br>1<br>5<br>—<br>2<br>3<br>4	walk<br>walk<br>walk<br>—<br>walk<br>walk<br>unresponsive	0 vs 2: 0.84<br>0 vs 3 (pair): 0.89<br>1 vs 2 (pair): 0.78<br>1 vs 3: 0.87	-	33
32	SS51046	medium	strong	2<br>(2,2)	0<br>—<br>1	turn<br>—<br>turn	0 vs 1 (pair): 0.48	-	34
33	SS29893	strong	strong	4<br>(4)	0<br>2<br>—<br>1<br>3	unclear<br>turn<br>—<br>unclear<br>turn	0 vs 1 (pair): 0.60<br>2 vs 3 (pair): 0.62	SS34574	35
34	SS34574	strong	strong	2<br>(2)	0<br>—<br>1	turn<br>—<br>turn	0 vs 1 (pair): 0.44	SS29893	36
35	R70H06	strong	strong	3<br>(2,2)	0<br>—<br>1<br>2	foreleg movement<br>—<br>foreleg movement<br>unresponsive	0 vs 1 (pair): 0.98	SS42740<br>SS42707	37
36	SS42740	strong	strong	2<br>(2,2)	0<br>—<br>1	foreleg movement<br>—<br>foreleg movement	0 vs 1 (pair): 0.97	R70H06<br>SS42707	38
37	SS28596	strong	strong	6<br>(4,5,5,4)	0<br>1<br>—<br>2<br>3<br>4<br>5	foreleg movement<br>foreleg movement<br>—<br>foreleg movement<br>foreleg movement<br>foreleg movement<br>foreleg movement	0 vs 2: 0.4<br>0 vs 3: 0.65<br>0 vs 4: 0.61<br>0 vs 5: 0.21<br>1 vs 2: 0.52<br>1 vs 3: 0.5<br>1 vs 4: 0.46<br>1 vs 5: 0.28	R86H08	39
38	SS25469	strong	strong	2<br>(3,5,3)	0<br>—<br>1	eye groom<br>—<br>eye groom	0 vs 1 (pair): 0.75	SS52106<br>SS52107<br>SS52108	40
39	SS31232	strong	strong	2<br>(2,2,2,2,2,2,<br>2,2,2,2,2)	0<br>—<br>1	proboscis extension<br>—<br>proboscis extension	0 vs 1 (pair): 0.81	SS30303<br>SS25451	41

Table 1 continued from previous page

No.	GAL4	Confocal SNR of smFP	2P SNR of OpGCaMP6f and tdTomato	Number ROIs (representative fly, (other flies))	ROI# (left-right seperated by —)	Encoding (left-right seperated by —)	Level synchronous activity (ROI# vs ROI#: Corr. coef.)	Redundant GAL4	Supp. Video#
40	SS30303		strong	2<br>(2,2)	0<br>—<br>1	proboscis extension<br>—<br>proboscis extension	0 vs 1 (pair): 0.89	SS31232<br>SS25451	42
41	SS25451	strong	strong	7<br>(5,6,6,6)	0<br>1<br>2<br>3<br>—<br>4<br>5<br>6	unresponsive<br>unresponsive<br>proboscis extension<br>unresponsive<br>—<br>proboscis extension<br>unresponsive<br>unresponsive	2 vs 4 (pair): 0.93	SS31232<br>SS30303	43
42	SS40134	strong	medium	2<br>(2,2,2)	0<br>—<br>1	unclear<br>unclear	0 vs 1 (pair): 0.34	-	44
43	SS29621	strong	strong	2<br>(5,4)	0<br>—<br>1	walk<br>walk	0 vs 1 (pair): 0.74	-	45
44	R69H10	strong	strong	3<br>(3)	0<br>1<br>2	unclear<br>unclear<br>unclear	0 vs 1: 0.21<br>0 vs 2: 0.07<br>1 vs 2: 0.02	-	46
45	SS51038	strong	strong	5<br>(5,3)	0<br>1<br>—<br>2<br>3<br>4	rest<br>unclear<br>—<br>unclear<br>unresponsive<br>rest	0 vs 2: -0.41<br>0 vs 4 (pair): 0.86<br>2 vs 4: -0.43	-	47
46	SS42008	strong	strong	4<br>(2,4,2)	0<br>1<br>—<br>2<br>3	walk<br>unclear<br>—<br>walk<br>unclear	0 vs 2 (pair): 0.53	SS42007	48
47	SS36118	strong	strong	6<br>(7,4,6,6,7)	0<br>2<br>3<br>—<br>1<br>4<br>5	unclear<br>unresponsive<br>puff<br>—<br>unclear<br>puff<br>rest	0 vs 1 (pair): 0.83<br>2 vs 4: 0.25<br>2 vs 5: 0.12<br>3 vs 4 (pair): 0.75<br>3 vs 5: 0.27	-	49
48	SS40619	medium	strong	2<br>(4,2,2,4)	0<br>—	walk<br>walk	0 vs 1 (pair): 0.74	-	50
49	SS45363	strong	strong	4<br>(7,4,5,4,4,6)	0<br>1<br>2<br>—<br>3	puff<br>puff<br>unclear<br>—<br>puff	0 vs 3: 0.83<br>1 vs 3: 0.85<br>2 vs 3: 0.71	-	51
50	SS52147	medium	medium	2<br>(2,2,2)	0<br>—<br>1	puff<br>—<br>puff	0 vs 1 (pair): 0.32	-	52
51	R38F09	-	strong	10<br>(5,6,6)	-	unresponsive	-	-	-
52	SS46269	strong	strong	12<br>(10,10)	-	unresponsive	-	-	-
53	SS25470	strong	strong	5<br>(4)	-	unresponsive	-	SS48406	-
54	SS25478	strong	strong	5<br>(4,3,4)	-	unresponsive	-	-	-
55	SS28382	strong	strong	6<br>(7,7)	-	unresponsive	-	-	-
56	SS29574	strong	strong	2<br>(2,3)	-	unresponsive	-	-	-
57	SS31899	strong	strong	8<br>(7,7)	-	unresponsive	-	-	-
58	SS33380	strong	strong	7<br>(6,8)	-	unresponsive	-	-	-
59	SS33433	strong	strong	2<br>(2,2)	-	unresponsive	-	-	-
60	SS38012	strong	strong	6<br>(6,5,6)	-	unresponsive	-	SS43528	-
61	SS38386	medium	strong	3<br>(4,4,4)	-	unresponsive	-	-	-
62	SS38687	strong	strong	3<br>(3,4)	-	unresponsive	-	-	-
63	SS46290	medium	medium	2<br>(-)	-	unresponsive	-	-	-
64	SS46300	strong	medium	2<br>(2)	-	unresponsive	-	-	-
65	SS48406	strong	strong	5<br>(7,6,10,7)	-	unresponsive	-	SS25470	-
66	SS48409	strong	strong	2<br>(2,2)	-	unresponsive	-	SS48632	-

Table 1 continued from previous page

No.	GAL4	Confocal SNR of smFP	2P SNR of OpGCaMP6f and tdTomato	Number ROIs (representative fly, (other flies))	ROI# (left-right seperated by —)	Encoding (left-right seperated by —)	Level synchronous activity (ROI# vs ROI#: Corr. coef.)	Redundant GAL4	Supp. Video#
67	SS49982	strong	medium	4 (3,2,2,3,4)	-	unresponsive	-	-	-
68	SS50004	strong	strong	2 (2,2)	-	unresponsive	-	-	-
69	SS50013	medium	medium	1 (-)	-	unresponsive	-	-	-
70	SS50652	strong	medium	3 (4,3)	-	unresponsive	-	-	-
71	SS36132	strong	-	-	-	not imaged (redundant)	-	SS36131 SS36133	-
72	SS36133	strong	-	-	-	not imaged (redundant)	-	SS36131 SS36132	-
73	SS38598	strong	-	-	-	not imaged (redundant)	-	SS38592	-
74	SS41808	strong	-	-	-	not imaged (redundant)	-	SS41822 SS41809 SS41820 SS41821	-
75	SS41809	strong	-	-	-	not imaged (redundant)	-	SS41822 SS41808 SS41820 SS41821	-
76	SS41820	strong	-	-	-	not imaged (redundant)	-	SS41822 SS41808 SS41809 SS41821	-
77	SS41821	strong	-	-	-	not imaged (redundant)	-	SS41822 SS41808 SS41809 SS41820	-
78	SS42007	strong	-	-	-	not imaged (redundant)	-	SS42008	-
79	SS42707	medium	-	-	-	not imaged (redundant)	-	SS42740 R70H06	-
80	SS43528	strong	weak	-	-	not imaged (undetectable in 2P; redundant)	-	SS38012	-
81	SS48632	medium	-	-	-	not imaged (redundant)	-	SS48409	-
82	SS51024	strong	-	-	-	not imaged (redundant)	-	SS51029	-
83	SS52108	strong	weak	-	-	not imaged (redundant)	-	SS25469 SS52106 SS52107	-
84	SS52106	medium	-	-	-	not imaged (redundant)	-	SS25469 SS52107 SS52108	-
85	SS52107	medium	-	-	-	not imaged (redundant)	-	SS25469 SS52106 SS52108	-
86	R86H08	medium	weak	-	-	not imaged (redundant)		SS28596	-
87	SS29889	strong (indistinguishable brain neurons)	-	-	-	not imaged (redundant)	-	SS29890	-
88	SS29890	strong (indistinguishable brain neurons)	-	-	-	not imaged (redundant)	-	SS29889	-
89	SS29605	-	unreliable expression	-	-	not imaged (unreliable expression)	-	-	-
90	SS31246	unreliable expression	unreliable expression	-	-	not imaged (unreliable expression)	-	-	-
91	SS46696	unreliable expression	unreliable expression	-	-	not imaged (unreliable expression)	-	-	-
92	R75E01	strong but with glia	-	-	-	not imaged (glia included)	-	-	-
93	SS37652	medium	weak	-	-	not imaged (undetectable)	-	-	-
94	SS41602	strong	weak	-	-	not imaged (undetectable)	-	-	-
95	SS43651	strong	weak	-	-	not imaged (undetectable)	-	-	-
96	SS44305	strong	weak	-	-	not imaged (undetectable)	-	-	-
97	SS46255	strong	weak	-	-	not imaged (undetectable)	-	-	-
98	SS41824	strong	weak	-	-	not imaged (undetectable)	-	-	-

Table 1 continued from previous page

No.	GAL4	Confocal SNR of smFP	2P SNR of OpGCaMP6f and tdTomato	Number ROIs (representative fly, (other flies))	ROI# (left-right seperated by —)	Encoding (left-right seperated by —)	Level synchronous activity (ROI# vs ROI#: Corr. coef.)	Redundant GAL4	Supp. Video#
99	SS25488	-	weak	-	-	not imaged (undetectable)	-	-	-
100	R81G07	weak	weak	-	-	not imaged (undetectable)	-	-	-
101	SS45635	weak	weak	-	-	not imaged (undetectable)	-	-	-
102	SS45648	weak	weak	-	-	not imaged (undetectable)	-	-	-
103	SS46290	weak	weak	-	-	not imaged (undetectable)	-	-	-
104	SS46847	weak	weak	-	-	not imaged (undetectable)	-	-	-
105	SS47868	weak	weak	-	-	not imaged (undetectable)	-	-	-
106	SS50282	weak	weak	-	-	not imaged (undetectable)	-	-	-
107	SS50829	weak	weak	-	-	not imaged (undetectable)	-	-	-
108	R88C08	weak	weak	-	-	not imaged (undetectable)	-	-	-
