# Supplementary material for: Ascending neurons convey behavioral state to integrative sensory and action selection brain regions
Source: Nat Neurosci. 2023 Mar 23;26(4):682–95. doi: 10.1038/s41593-023-01281-z (PMC10076225; doi:10.1038/s41593-023-01281-z)
Supplement: Supplementary file 2 — Reporting Summary [file 41593_2023_1281_MOESM2_ESM.pdf]

Corresponding author(s): Pavan Ramdya
Last updated by author(s): Dec 23, 2022

# Reporting Summary

Nature Portfolio wishes to improve the reproducibility of the work that we publish. This form provides structure for consistency and transparency in reporting. For further information on Nature Portfolio policies, see our Editorial Policies and the Editorial Policy Checklist.

## Statistics

For all statistical analyses, confirm that the following items are present in the figure legend, table legend, main text, or Methods section.

| n/a | Confirmed | |
|---|---|---|
| ☐ | ☒ | The exact sample size ($n$) for each experimental group/condition, given as a discrete number and unit of measurement |
| ☐ | ☒ | A statement on whether measurements were taken from distinct samples or whether the same sample was measured repeatedly |
| ☐ | ☒ | The statistical test(s) used AND whether they are one- or two-sided<br>*Only common tests should be described solely by name; describe more complex techniques in the Methods section.* |
| ☐ | ☒ | A description of all covariates tested |
| ☐ | ☒ | A description of any assumptions or corrections, such as tests of normality and adjustment for multiple comparisons |
| ☐ | ☒ | A full description of the statistical parameters including central tendency (e.g. means) or other basic estimates (e.g. regression coefficient) AND variation (e.g. standard deviation) or associated estimates of uncertainty (e.g. confidence intervals) |
| ☐ | ☒ | For null hypothesis testing, the test statistic (e.g. $F$, $t$, $r$) with confidence intervals, effect sizes, degrees of freedom and $P$ value noted<br>*Give P values as exact values whenever suitable.* |
| ☒ | ☐ | For Bayesian analysis, information on the choice of priors and Markov chain Monte Carlo settings |
| ☒ | ☐ | For hierarchical and complex designs, identification of the appropriate level for tests and full reporting of outcomes |
| ☐ | ☒ | Estimates of effect sizes (e.g. Cohen's $d$, Pearson's $r$), indicating how they were calculated |

*Our web collection on statistics for biologists contains articles on many of the points above.*

## Software and code

Policy information about availability of computer code

| | |
|---|---|
| Data collection | Two-photon microscope images were acquired using ThorImage 3.1 software. Data synchronization was performed using ThorSync 3.1 software. Behavior images were acquired using custom Python scripts. Confocal images were acquired using Zen 2011 14.0 software. |
| Data analysis | Data analyses were performed using custom code written in Python 3. The code is available in the following repository: https://github.com/NeLy-EPFL/Ascending_neuron_screen_analysis_pipeline<br>Fiji v.2.9.0 software was used to generate standard deviation z-projections of image stacks, combine monochromatic images to generate RGB images, mask MCFO confocal images, and trace neurons.<br>AxoID software was developed and used to track ROIs in two-photon imaging data. The code is available in the following repository: https://github.com/NeLy-EPFL/AxoID<br>Code for brain and VNC confocal image registration can be found at: https://github.com/NeLy-EPFL/MakeAverageBrain/tree/workstation<br>MCFO brain and VNC confocal image registration was performed using the Computational Morphometry Toolkit: https://www.nitrc.org/projects/cmtk |

For manuscripts utilizing custom algorithms or software that are central to the research but not yet described in published literature, software must be made available to editors and reviewers. We strongly encourage code deposition in a community repository (e.g. GitHub). See the Nature Portfolio guidelines for submitting code & software for further information.

## Data

Policy information about availability of data

All manuscripts must include a data availability statement. This statement should provide the following information, where applicable:

- Accession codes, unique identifiers, or web links for publicly available datasets
- A description of any restrictions on data availability
- For clinical datasets or third party data, please ensure that the statement adheres to our policy

Data are available at: https://dataverse.harvard.edu/dataverse/AN . Due to data storage limits, this does not include raw behavior camera images or raw two-photon imaging files. This repository includes: synchronized neural fluorescence, behavior, and ball rotation velocities; raw and traced MCFO confocal image data; neural data used for regression analyses, responses of PE-ANs, and neural responses on and off of the spherical treadmill; behavioral data as well as the deep-learning model for measuring proboscis extensions and annotations for training the behavior classifier; linear regression results; a machine-readable version of Table S1.
For brain and VNC image registration, templates can be downloaded here: https://www.janelia.org/open-science/jrc-2018-brain-templates.
Neuropil region masks can be downloaded here: https://v2.virtualflybrain.org.

## Human research participants

Policy information about studies involving human research participants and Sex and Gender in Research.

| | |
|---|---|
| Reporting on sex and gender | N/A |
| Population characteristics | N/A |
| Recruitment | N/A |
| Ethics oversight | N/A |

Note that full information on the approval of the study protocol must also be provided in the manuscript.

# Field-specific reporting

Please select the one below that is the best fit for your research. If you are not sure, read the appropriate sections before making your selection.

☒ Life sciences ☐ Behavioural & social sciences ☐ Ecological, evolutionary & environmental sciences

For a reference copy of the document with all sections, see nature.com/documents/nr-reporting-summary-flat.pdf

# Life sciences study design

All studies must disclose on these points even when the disclosure is negative.

| | |
|---|---|
| Sample size | The study was designed as a functional and anatomical screen of many Drosophila driver lines. Each line was functionally examined in 2-5 animals each. Anatomical studies were very reliable across samples. AN encodings were qualitatively reliable for the same driver line across animals aside from differences in signal-to-noise ratio as well as minor variability in the number of ROIs for a subset of driver lines. No statistical methods were used to pre-determine sample sizes. Our sample sizes are justified by AN functional response reliability and the constraint of the time required to functionally screen 70 driver lines in behaving animals.<br>The data presented in this manuscript were acquired from 245 flies:<br>108 flies for confocal imaging of smFP expression.<br>70 flies for two-photon AN imaging during behavior.<br>42 flies for tracing single AN morphologies using MCFO.<br>7 flies for imaging MCFO single neuron morphologies at high magnification.<br>7 flies for confocal imaging of syt:GFP.<br>3 flies for comparing puff-AN responses to air versus carbon dioxide.<br>8 flies for examining the ramping increase in PE-AN activity during PE trains. |
| Data exclusions | Data from two-photon recordings of behaving flies were excluded for animals and trials in which we observed abnormal limb movements, or low vitality. Two-photon imaging data were also excluded if they suffered from optical occlusions due to tissue debris, or extreme motion artifacts resulting from animal behavior. |
| Replication | For the two-photon functional imaging screen, 2-5 replicates (animals) were recorded for each genotype. All attempts at replication were successful. Recordings from one representative fly of each genotype were used in linear modeling. This decision was made due to the difficulty, for some genotypes, of confidently identifying the same neurons across animals.<br>For the SS31232 (PE-ANs) line, we analyzed 25 PE-trains from 8 replicates (animals).<br>For the SS36112 (puff-ANs) line, we analyzed responses to air versus carbon dioxide for 3 replicates (animals).<br>To measure syt:GFP expression in each genotype, 3-6 replicates (animals) were analyzed. One representative animal is shown for each of 7 |

lines studied in-depth.
To measure single neuron labeling with MCFO, 2-7 replicates (animals) were analyzed per genotype. One representative animal is shown for each of 42 lines. The same number of replicates were used for high-magnification studies of single neuron morphologies.
To examine the expression of smFP, 2-3 replicates (animals) were examined for each genotype. One representative example is shown for each of the 108 lines.

Randomization

Because we performed a functional screen without prior hypotheses, the experiments were not randomized.

Blinding

Because we performed a functional screen without prior hypotheses, the data collection and analyses were not performed blind to the conditions of the experiments.

# Reporting for specific materials, systems and methods

We require information from authors about some types of materials, experimental systems and methods used in many studies. Here, indicate whether each material, system or method listed is relevant to your study. If you are not sure if a list item applies to your research, read the appropriate section before selecting a response.

## Materials & experimental systems

| n/a | Involved in the study |
|---|---|
| ☐ | ☒ Antibodies |
| ☒ | ☐ Eukaryotic cell lines |
| ☒ | ☐ Palaeontology and archaeology |
| ☐ | ☒ Animals and other organisms |
| ☒ | ☐ Clinical data |
| ☒ | ☐ Dual use research of concern |

## Methods

| n/a | Involved in the study |
|---|---|
| ☒ | ☐ ChIP-seq |
| ☒ | ☐ Flow cytometry |
| ☒ | ☐ MRI-based neuroimaging |

## Antibodies

Antibodies used

- Mouse Anti-nc82,  Bruchpilot (DSHB, RRID: AB_2314866)
- Rabbit anti-GFP (Thermofisher, RRID: AB_2536526)
- rabbit anti-HA-tag (Cell Signaling Technology, RRID: AB_1549585)
- rat anti-FLAG-tag (DYKDDDDK; Novus, RRID:AB_1625981)
- rabbit anti-V5-tag (GKPIPNPLLGLDST) conjugated with DyLight 550 (Cayman Chemical, 11261)
- rabbit polyclonal anti-DsRed (Takara Biomedical Technology, RRID: AB_10013483)
- goat anti-rabbit secondary antibody conjugated with Alexa 488 (Thermofisher, RRID: AB_143165)
- goat anti-mouse secondary antibody conjugated with Alexa 633 (Thermofisher, RRID: AB_2535719)
- donkey anti-rabbit secondary antibody conjugated with AlexaFluor 594(Jackson ImmunoResearch Labs, RRID:AB_2340621)
- donkey anti-rat secondary antibody conjugated with AlexaFluor 647 (Jackson ImmunoResearch Labs, RRID:AB_2340694)
- donkey anti-mouse secondary antibody conjugated with AlexaFluor 488 (Jackson ImmunoResearch Labs, RRID:AB_2341099)
- donkey anti-rabbit secondary antibody conjugated with Cy3 (Jackson ImmunoResearch Labs, RRID:AB_2307443)

Validation

Primary antibodies were validated by the suppliers as follows: Rabbit anti-GFP (Thermofisher, RRID: AB_2536526) was validated through relative expression, rabbit anti-HA-tag (Cell Signaling Technology, RRID: AB_1549585) was validated through immunohistochemical expression analysis, and rabbit polyclonal anti-DsRed (Takara Biomedical Technology, RRID: AB_10013483) was validated by western blot. No manufacturer notes are available for the validation of other primary antibodies. No additional validation was performed.
- DSHB - https://dshb.biology.uiowa.edu/nc82
- Thermofisher - https://www.thermofisher.com/antibody/product/
- Cell Signaling Technology - https://www.cellsignal.com/products/primary-antibodies/ha-tag-c29f4-rabbit-mab/3724
- Cayman Chemical - https://www.caymanchem.com/product/11261/rabbit-anti-v5-tag-igg%3Adylight%C2%AE-550
- Takara Biomedical Technology - https://www.takarabio.com/products/antibodies-and-elisa/fluorescent-protein-antibodies/red-fluorescent-protein-antibodies
- Jackson ImmunoResearch Labs - https://www.jacksonimmuno.com/

## Animals and other research organisms

Policy information about studies involving animals; ARRIVE guidelines recommended for reporting animal research, and Sex and Gender in Research

Laboratory animals

Female Drosophila melanogaster flies 3-6 days post-eclosion (dpe) from the following driver lines were used in this study:
Split-Gal4 lines (SS*****),
GMR Gal4 lines,
MAN-spGal4 (; VT50660-AD; VT14014-DBD),
MCFO-5 (R57C10-Flp2::PEST in su(Hw)attP8; ; HA-V5-FLAG), MCFO-7 (R57C10-Flp2::PEST in attP18;;HA-V5-FLAG-OLLAS),
UAS-syt:GFP (P{w[+mC]=UAS-syt.eGFP}1, w[*]; ; ),
UAS-OpGCaM6f; UAS-tdTomato (; P{20XUAS-IVS-Syn21-OpGCamp6F-p10} su(Hw)attp5; P{w[+mC]=UAS-tdTom.S}3),
UAS-smFP (; ; 10xUAS-IVS-myr::smGdP-FLAG (attP2))

| | |
|---|---|
| Wild animals | No wild animals were used. |
| Reporting on sex | All studies were performed on female flies due to their larger body size. This property facilitates neural data analysis and behavioral quantification. |
| Field-collected samples | No field-collected samples were used. |
| Ethics oversight | All experiments were performed in compliance with relevant national (Switzerland) and institutional (EPFL) ethical regulations. |

Note that full information on the approval of the study protocol must also be provided in the manuscript.
