# Supplementary material for: Ascending neurons convey behavioral state to integrative sensory and action selection brain regions
Source: Nat Neurosci. 2023 Mar 23;26(4):682–95. doi: 10.1038/s41593-023-01281-z (PMC10076225; doi:10.1038/s41593-023-01281-z)

20190521-SS36131-tdTomGC6fopt-fly1-001

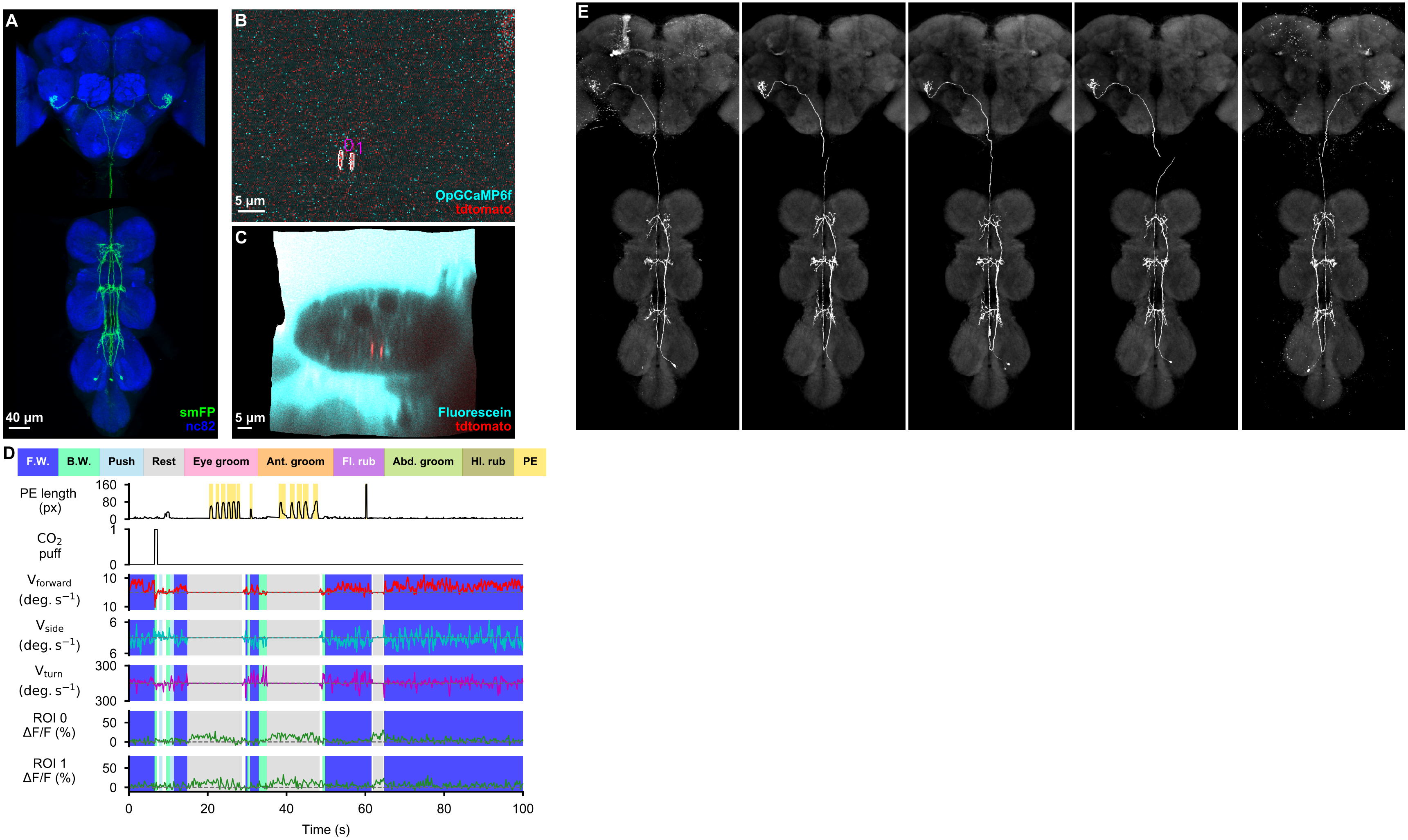

20190531-SS38592-tdTomGC6fopt-fly3-004

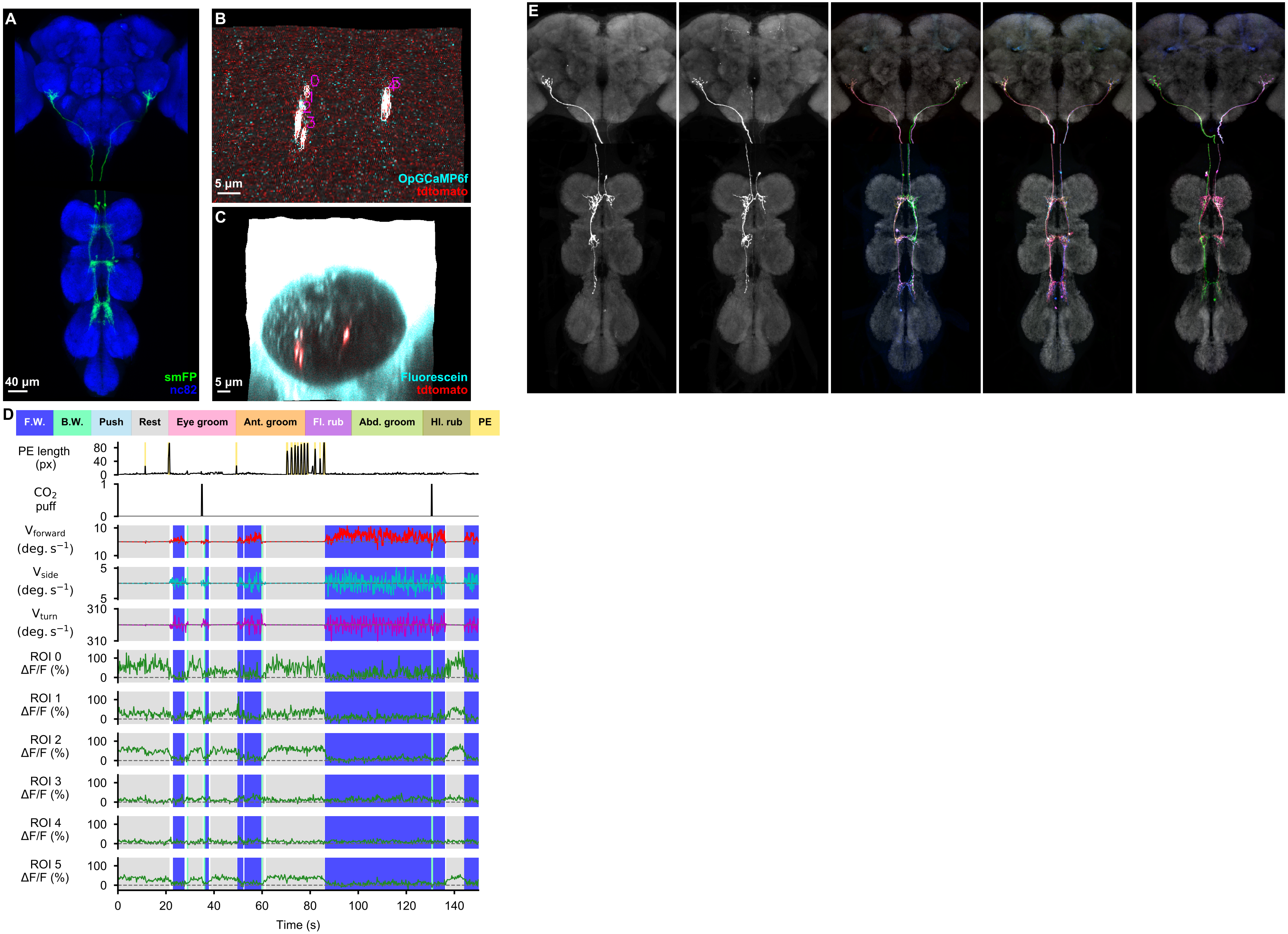

20190311-SS27485-tdTomGC6fopt-fly2-004

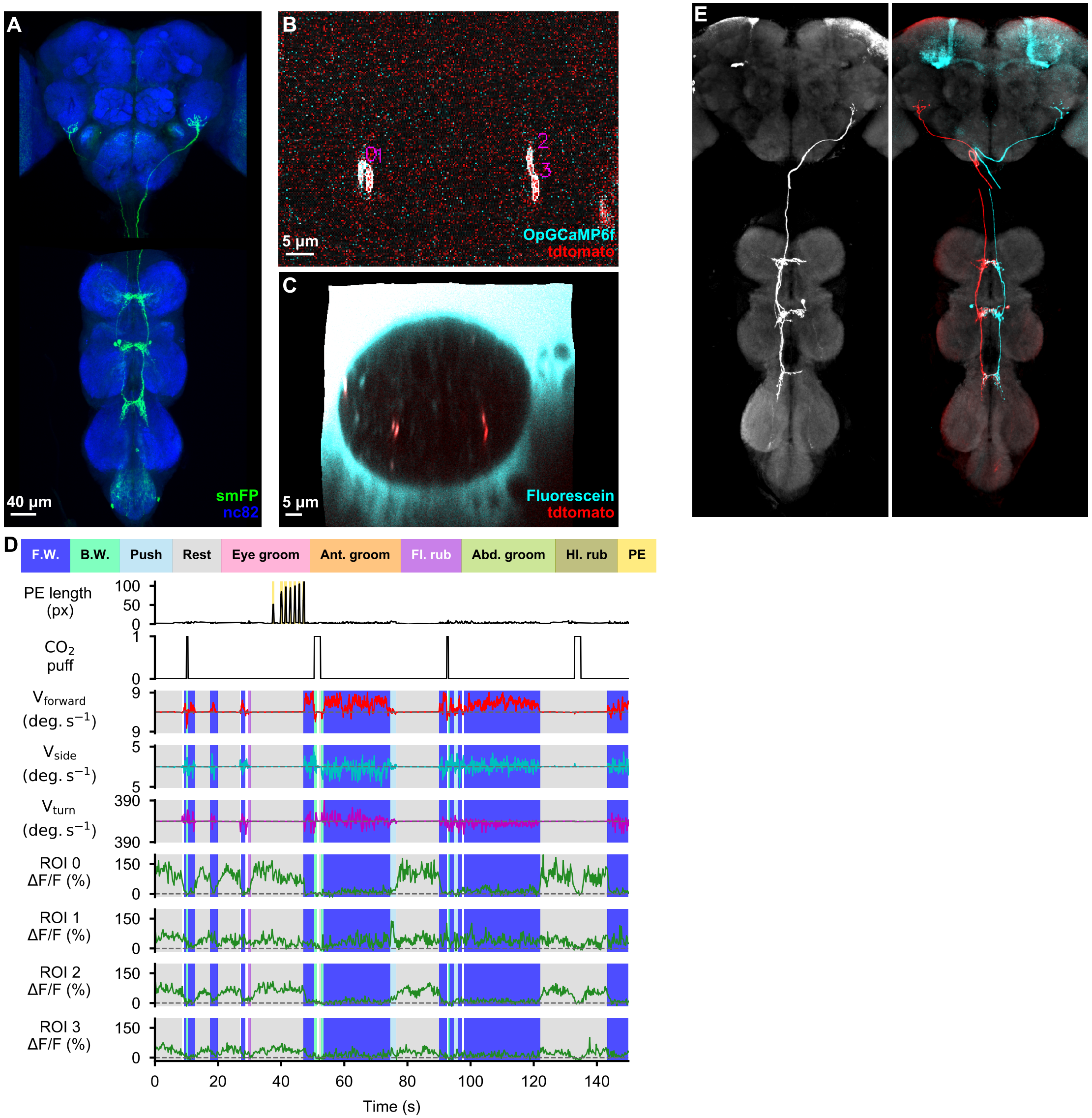

20190624-SS41822-tdTomGC6fopt-fly2-002

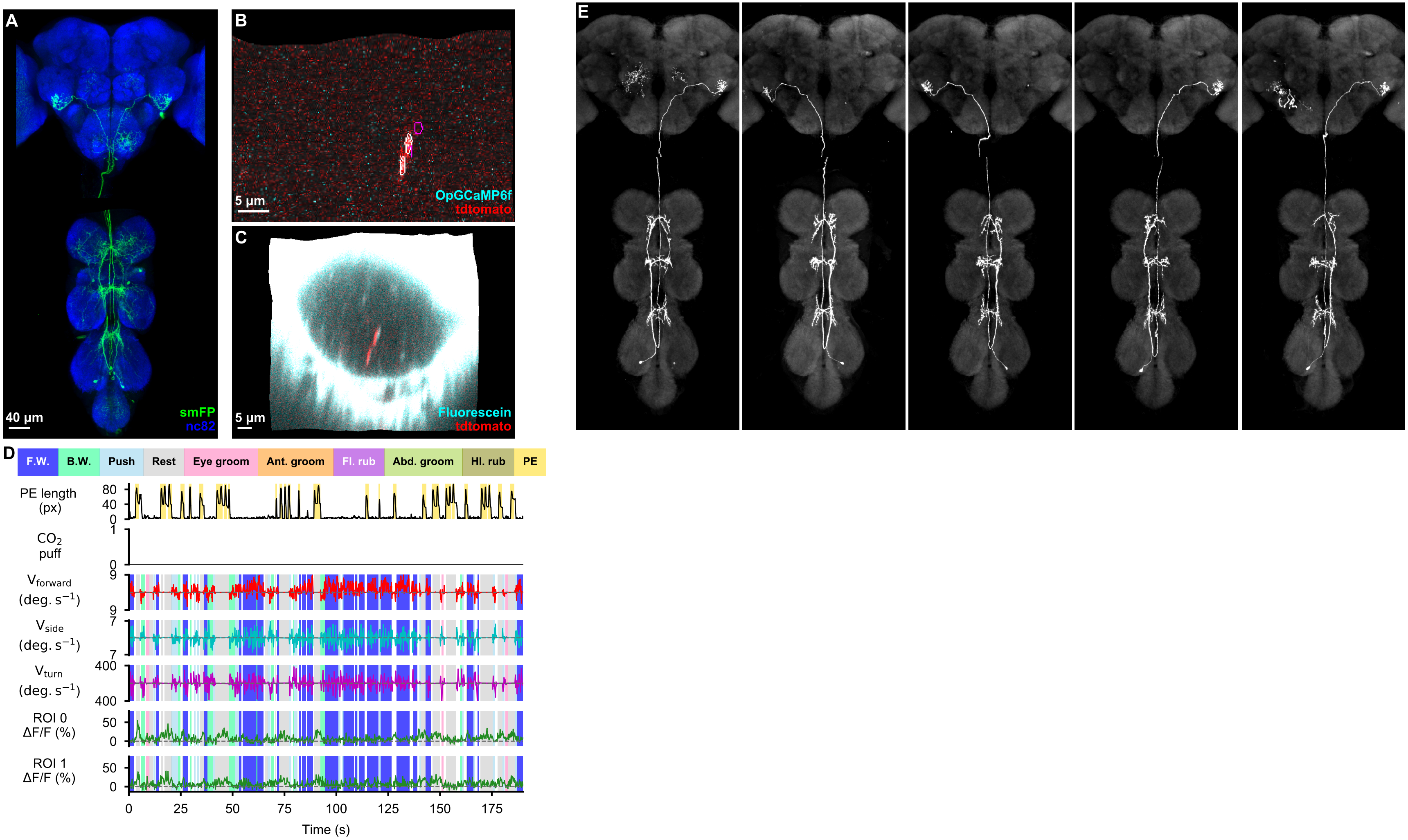

20190606-SS38624-tdTomGC6fopt-fly3-003

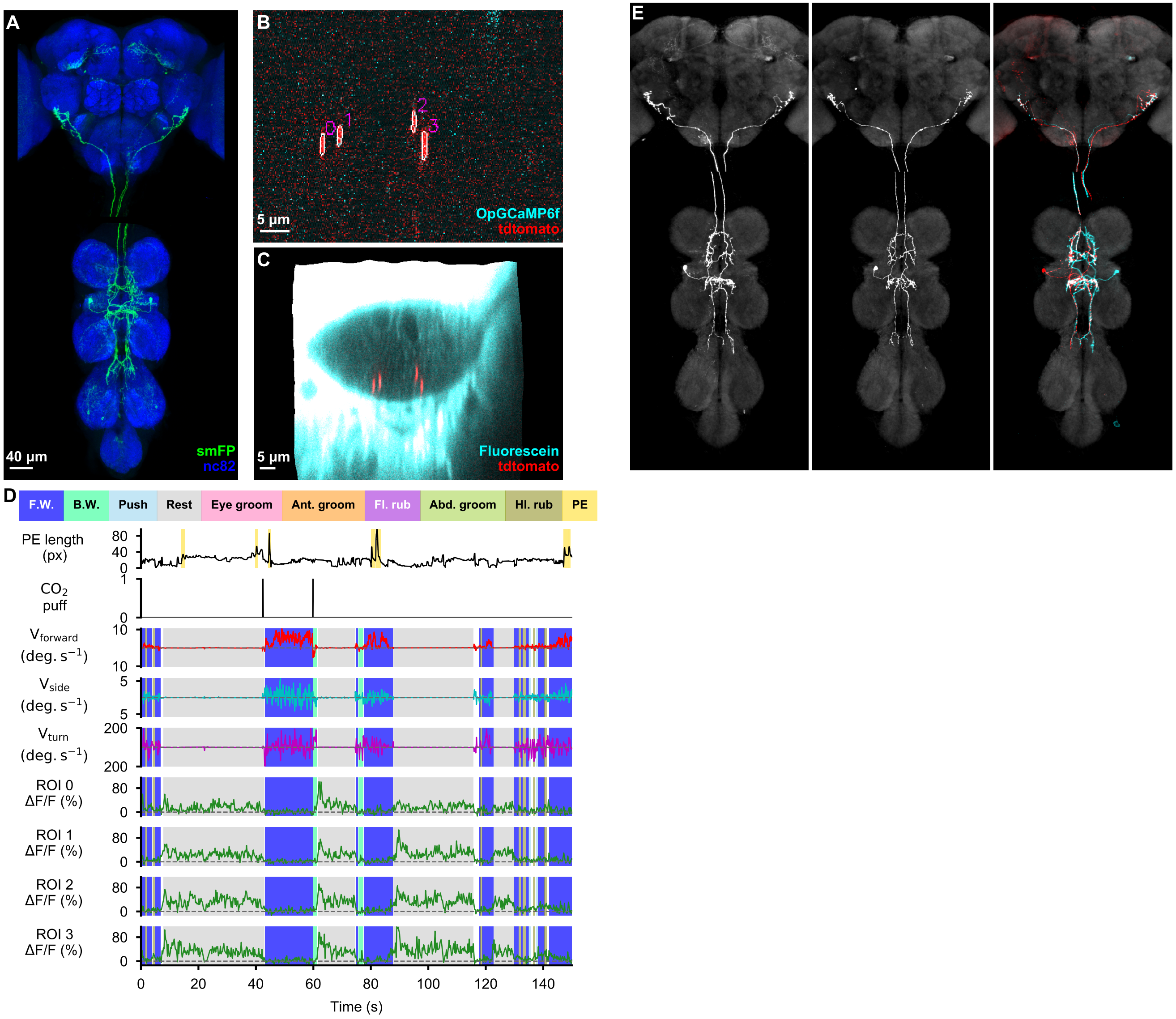

20190719-SS45605-tdTomGC6fopt-fly1-003

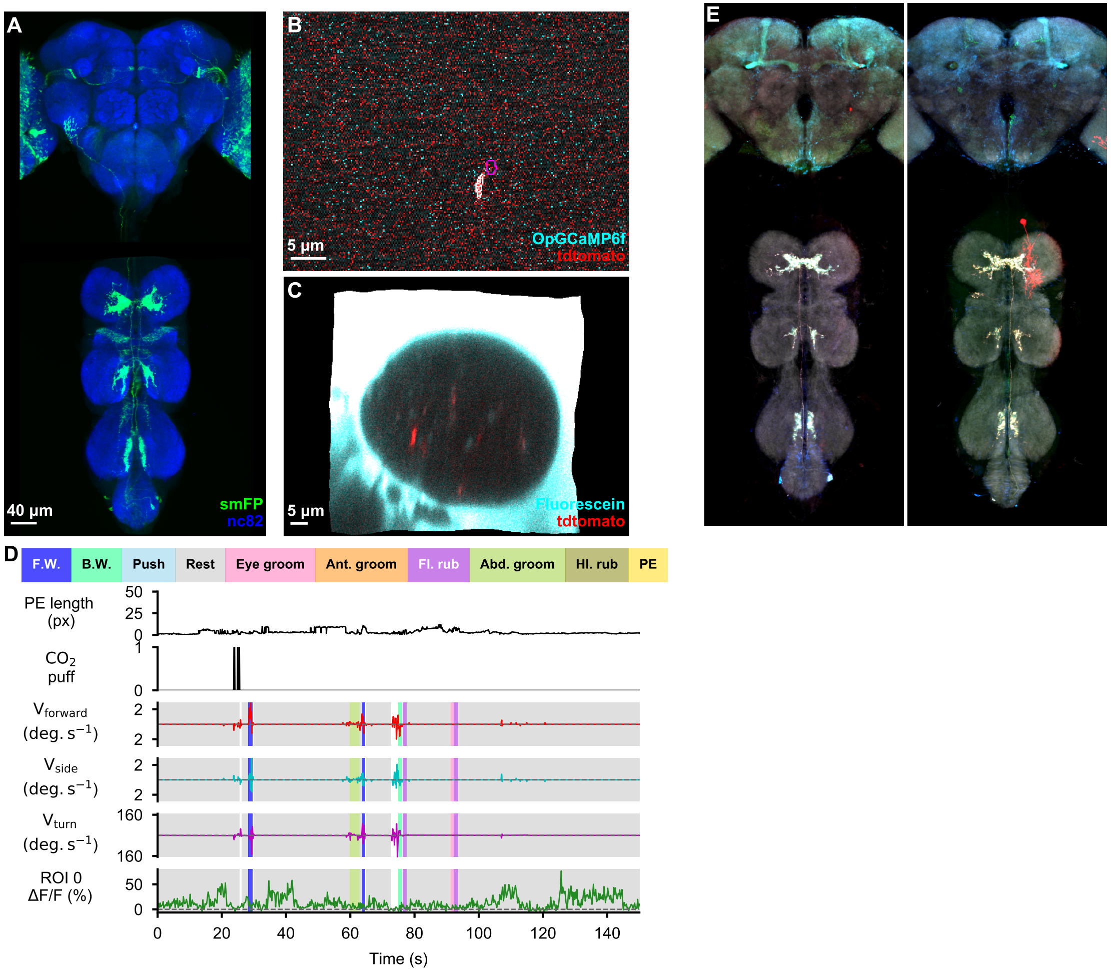

20190712-SS43652-tdTomGC6fopt-fly2-002

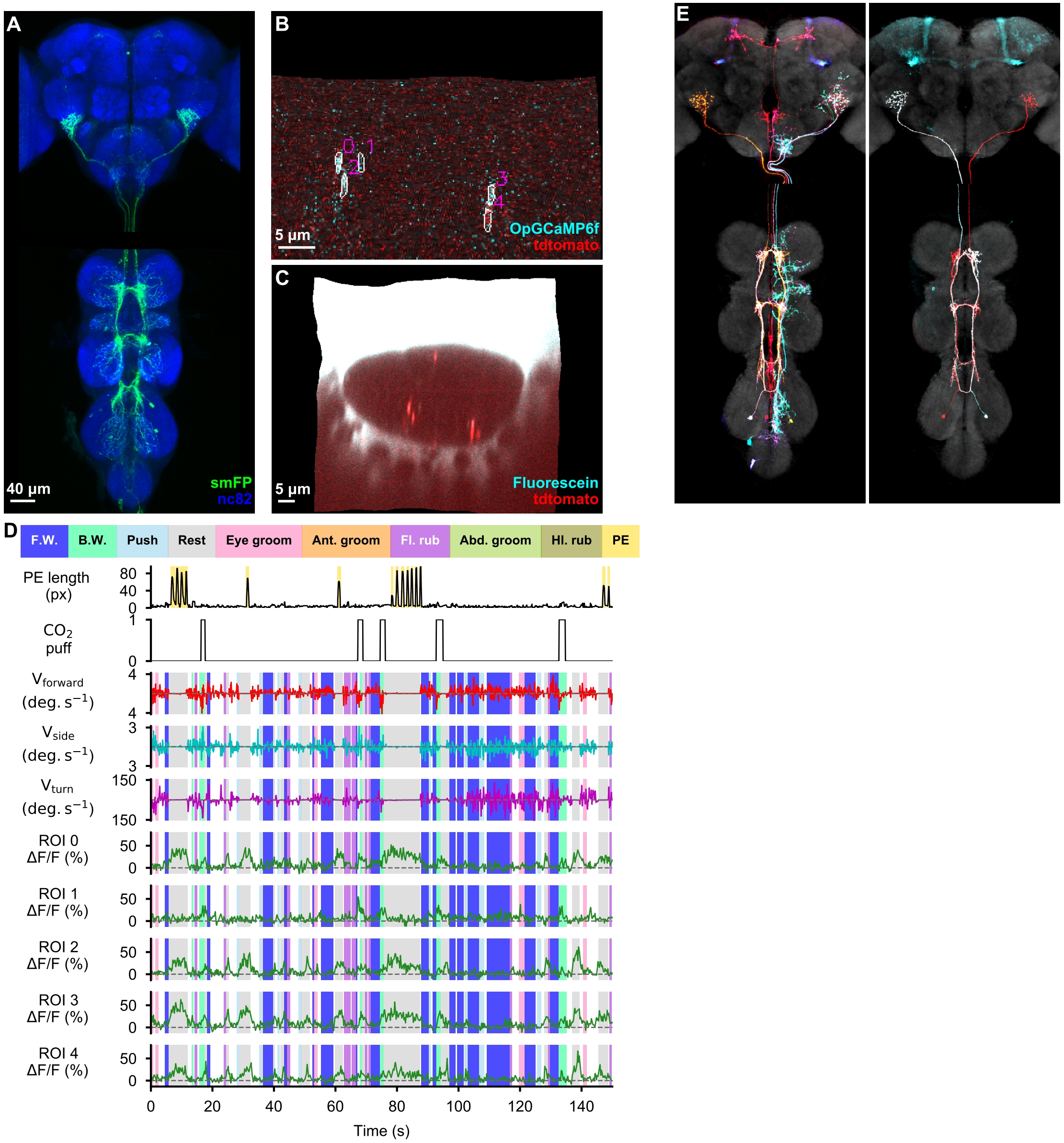

20190517-SS36112-tdTomGC6fopt-fly1-007

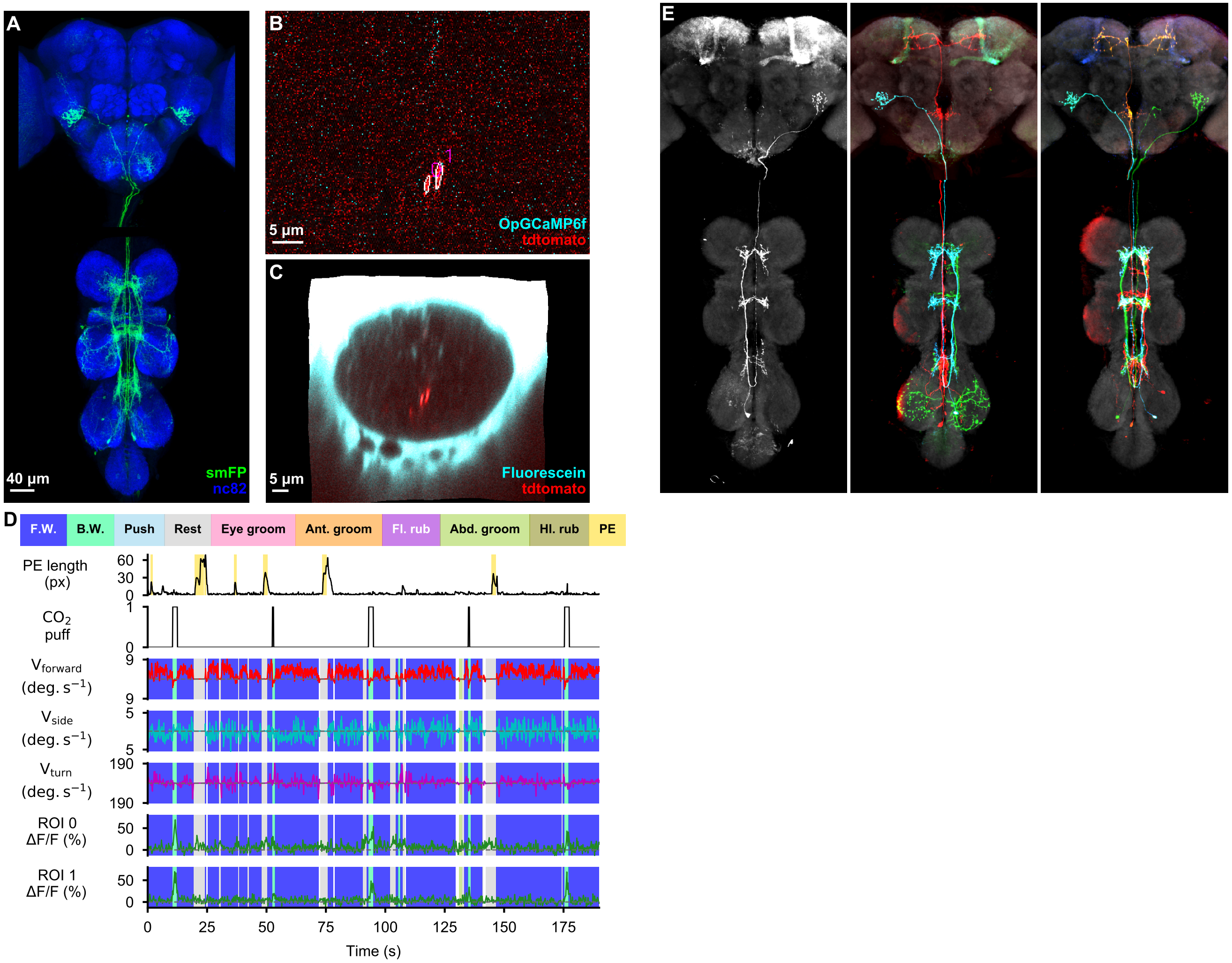

20190625-SS41806-tdTomGC6fopt-fly4-007

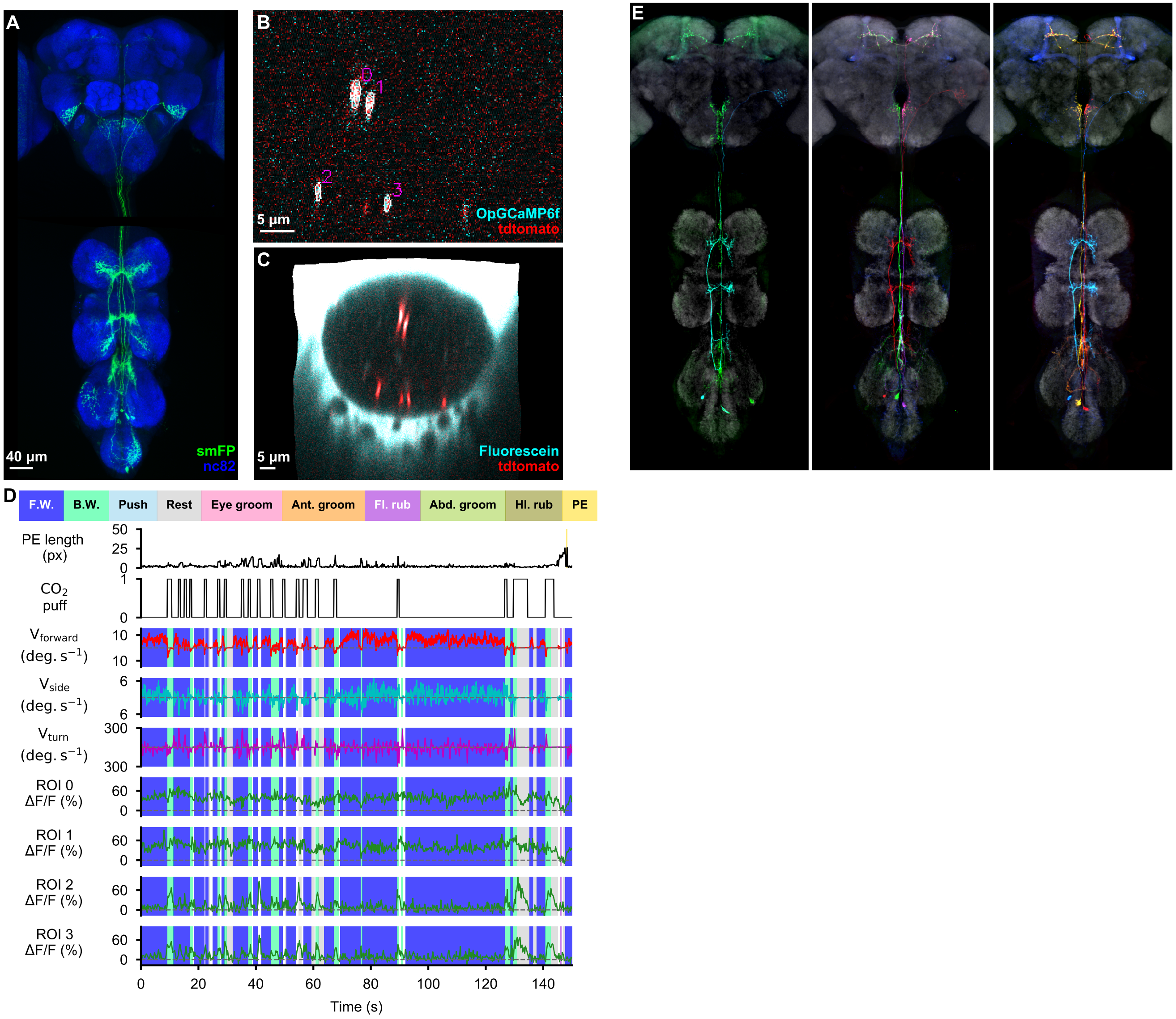

20190604-SS38631-tdTomGC6fopt-fly2-004

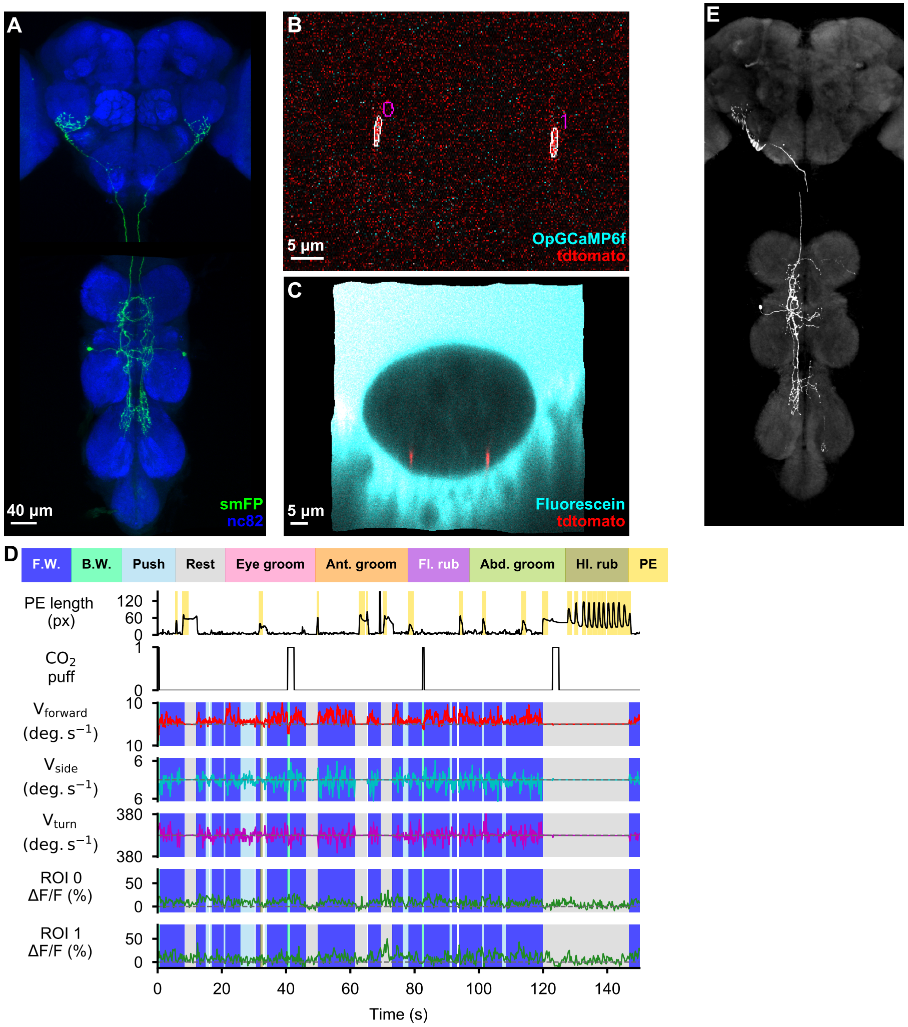

20190906-SS51029-tdTomGC6fopt-fly4-006

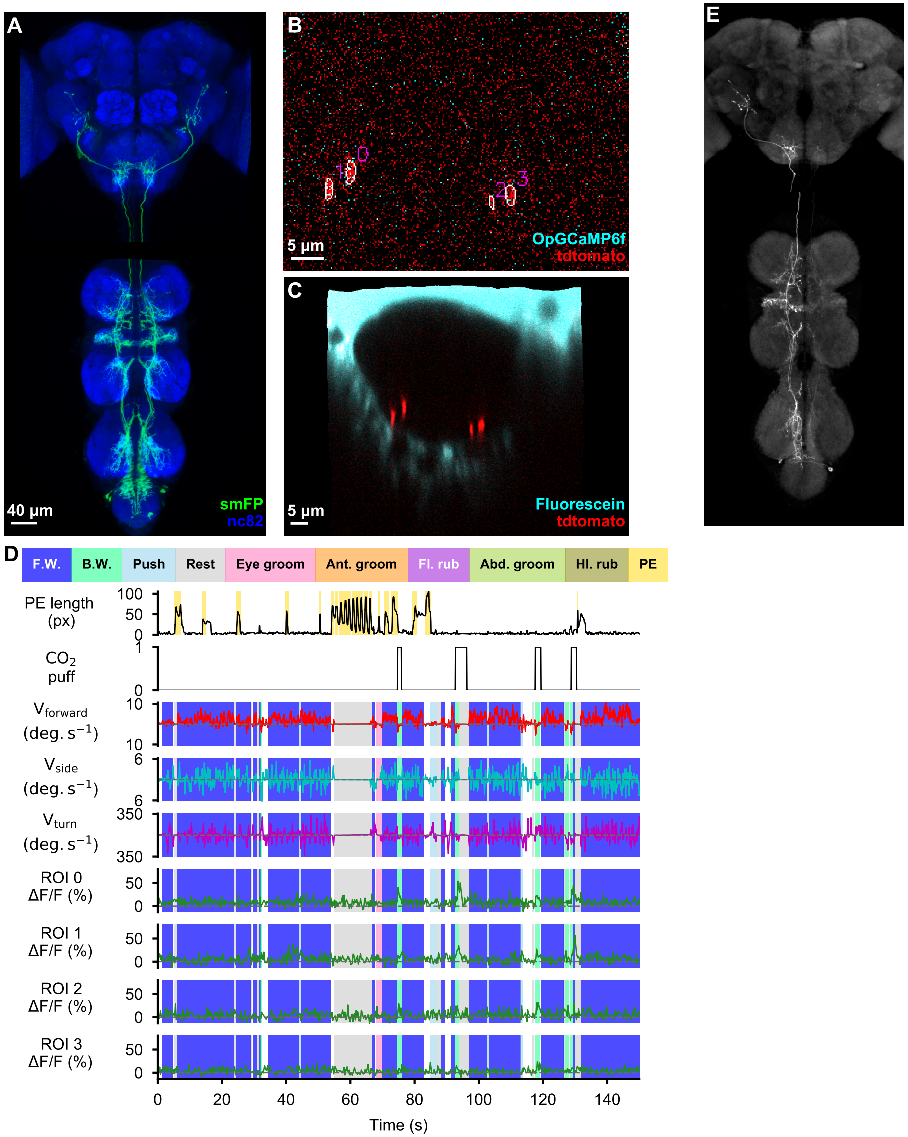

20181125-R85A11-tdTomGC6fopt-fly1-002

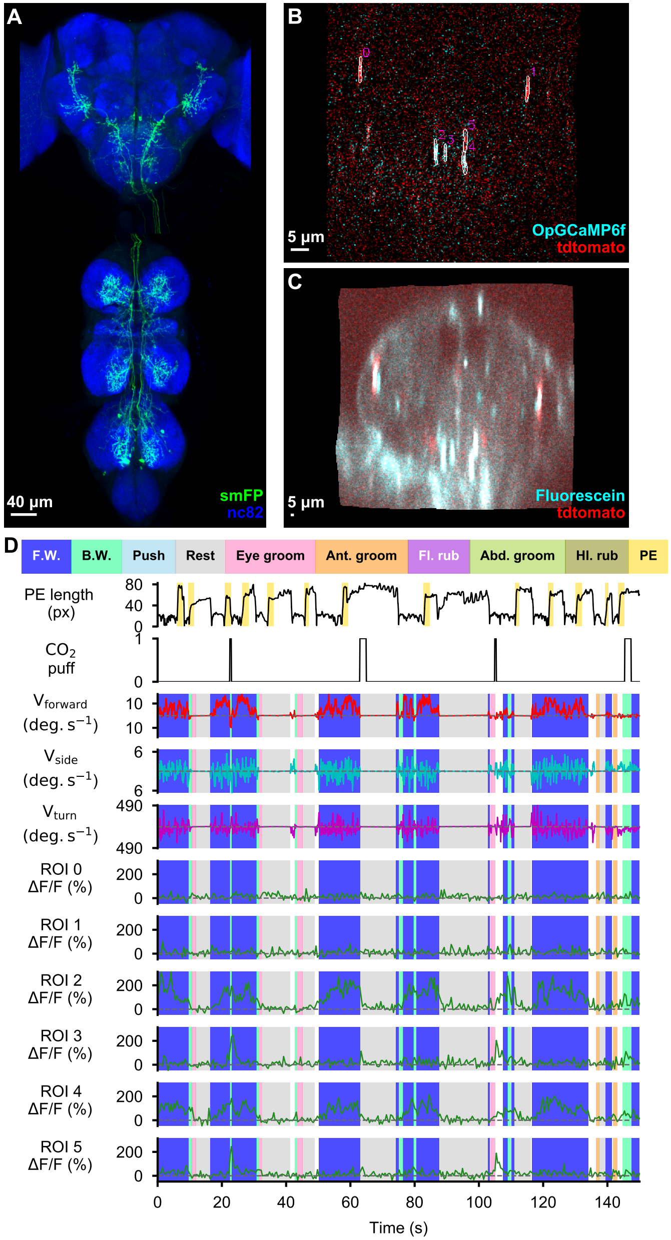

E

MCFO experiment was not performed.

20190610-SS40489-tdTomGC6fopt-fly3-004

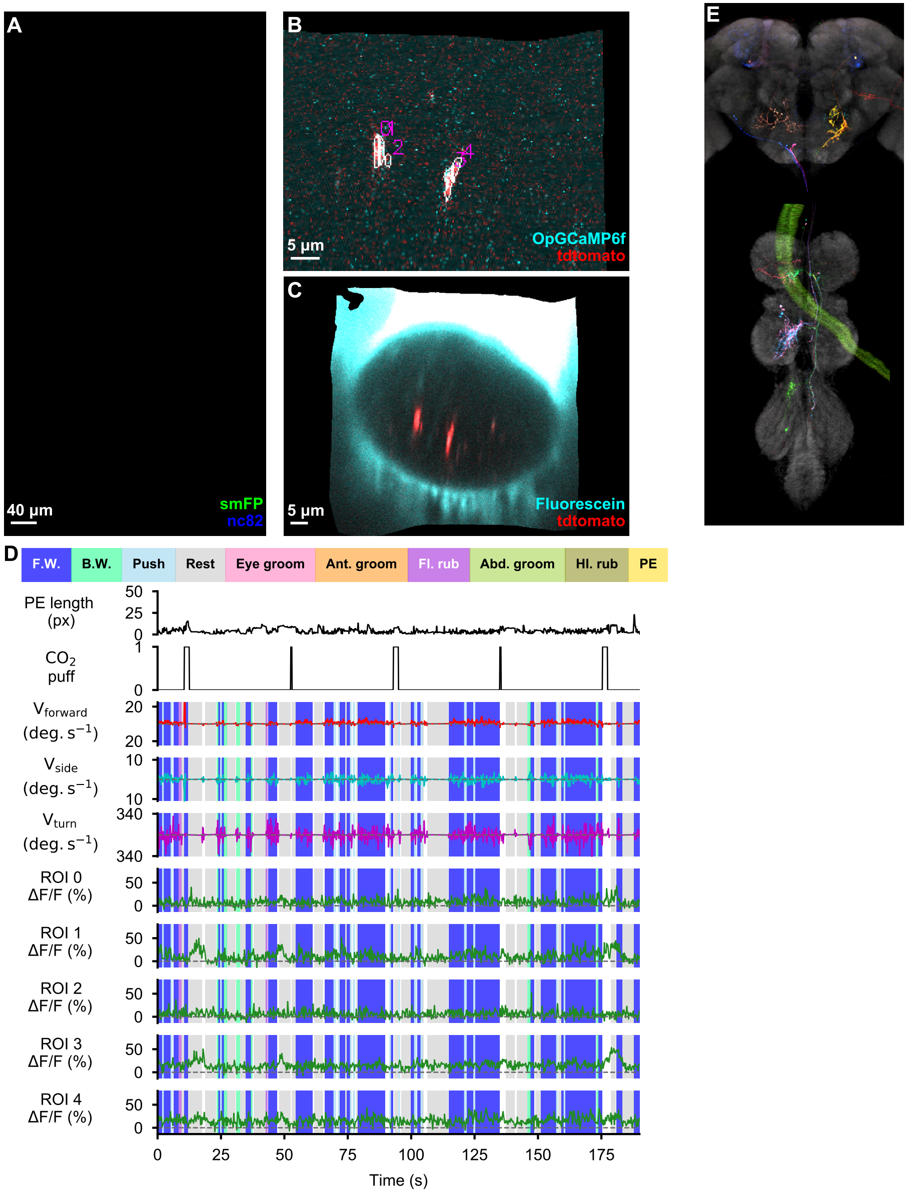

20190417-SS31480-tdTomGC6fopt-fly1-001

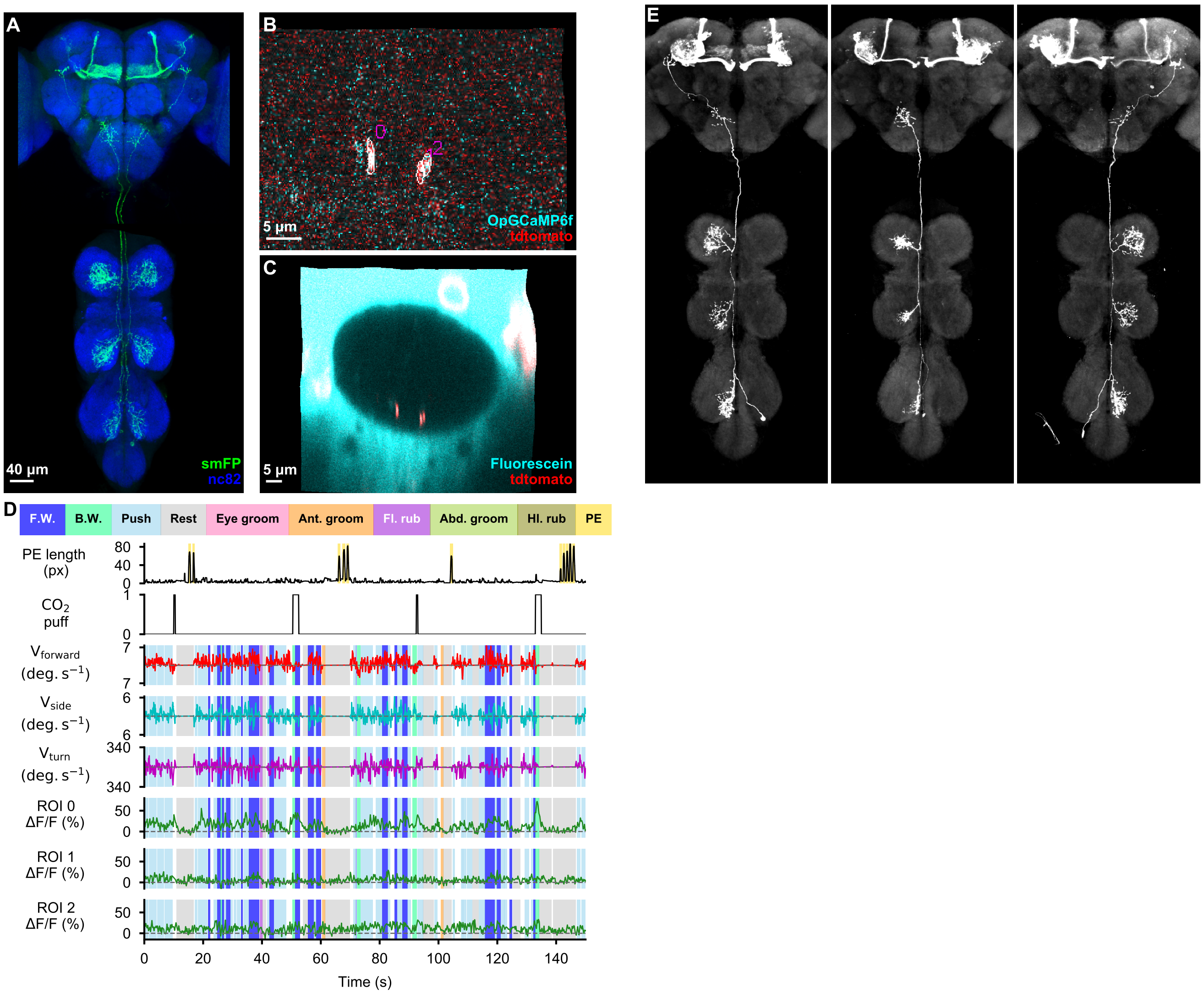

20190924-SS51021-tdTomGC6fopt-fly1-004

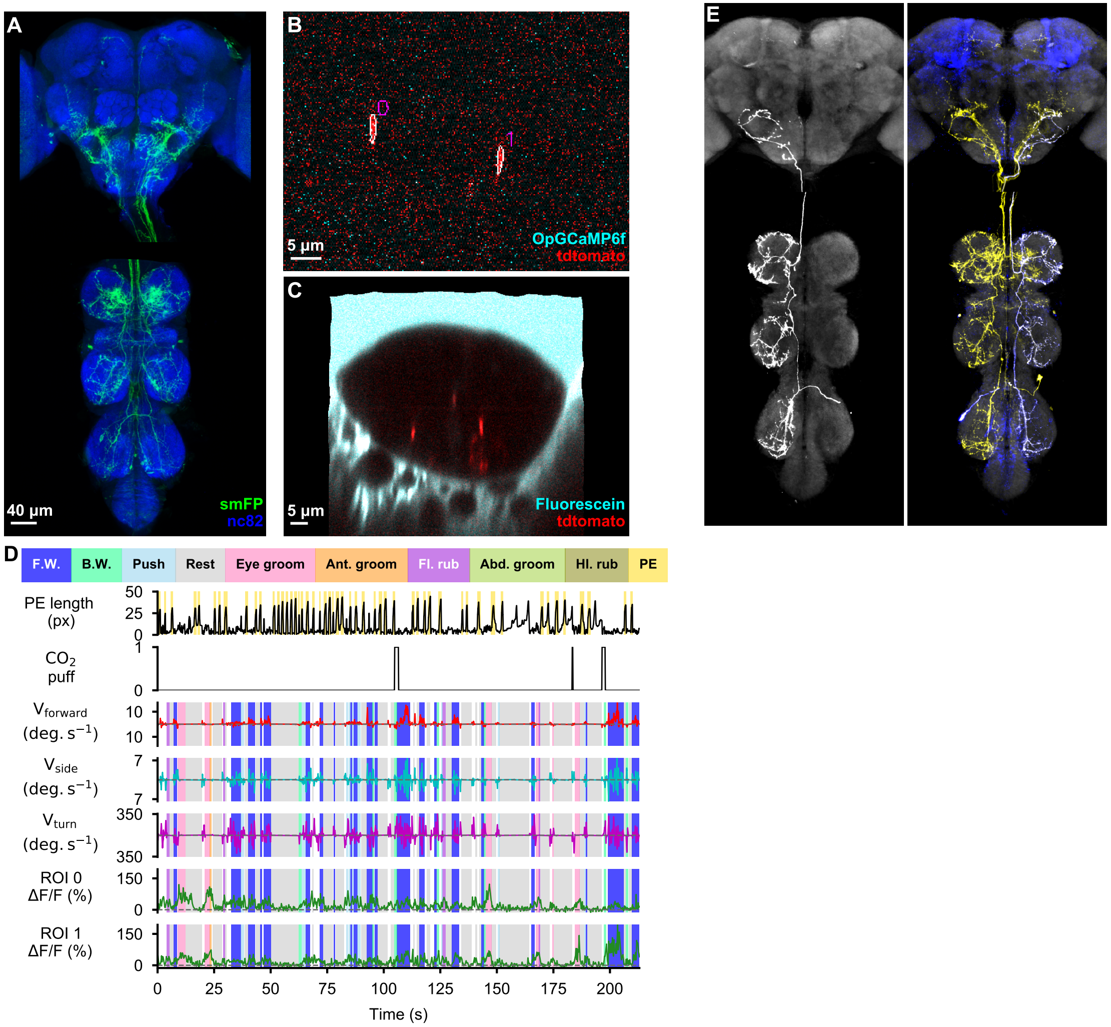

20190904-SS51017-tdTomGC6fopt-fly3-008

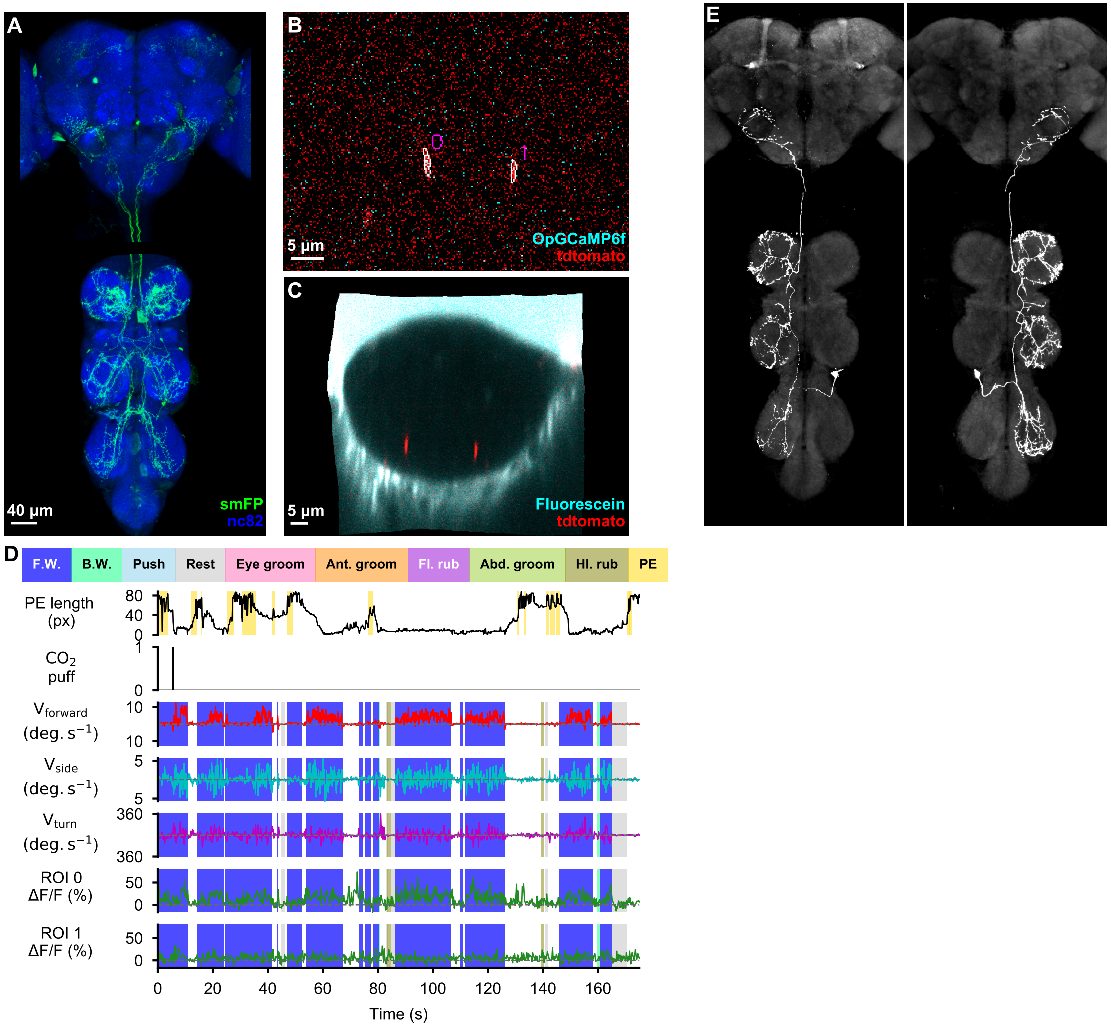

20190410-SS31456-tdTomGC6fopt-fly1-006

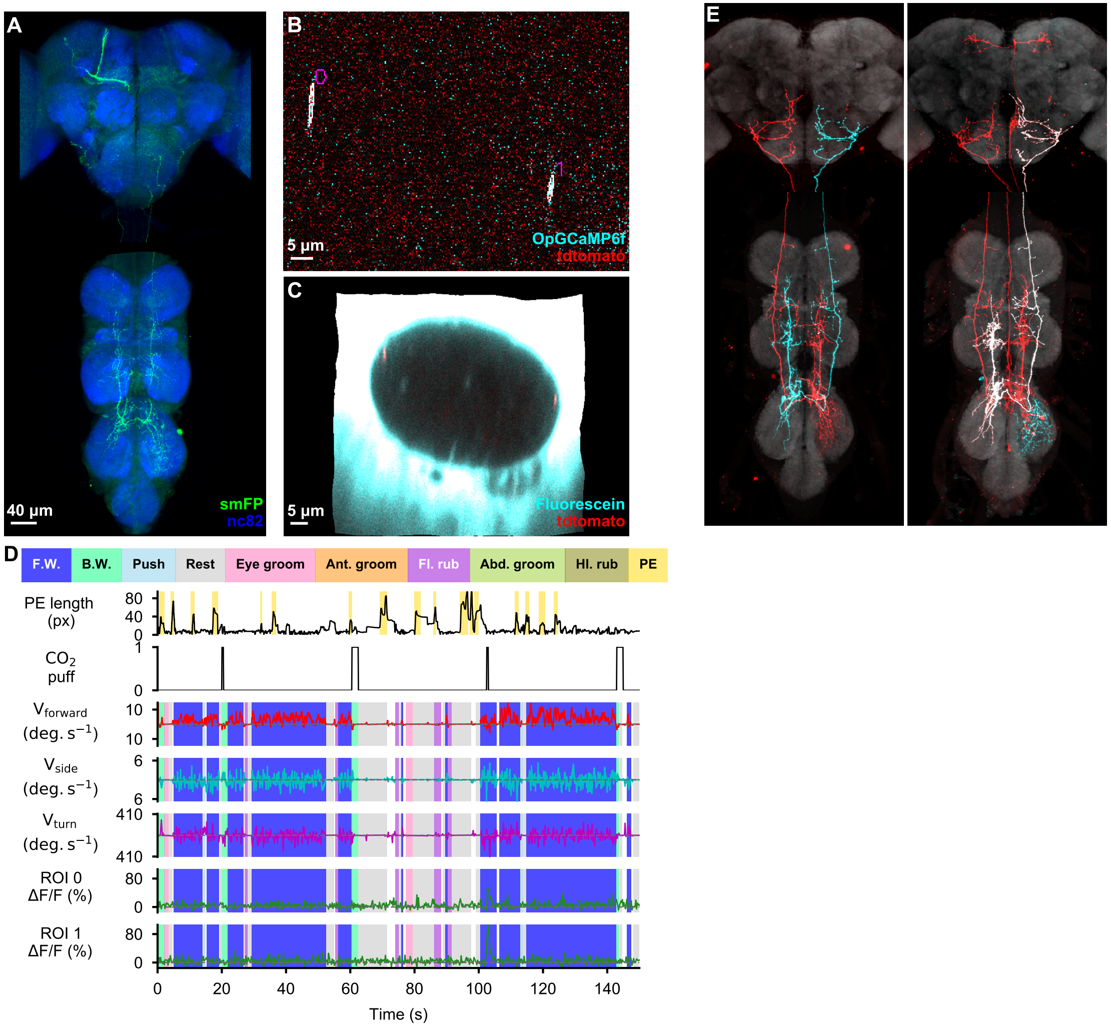

20190730-SS46233-tdTomGC6fopt-fly2-005

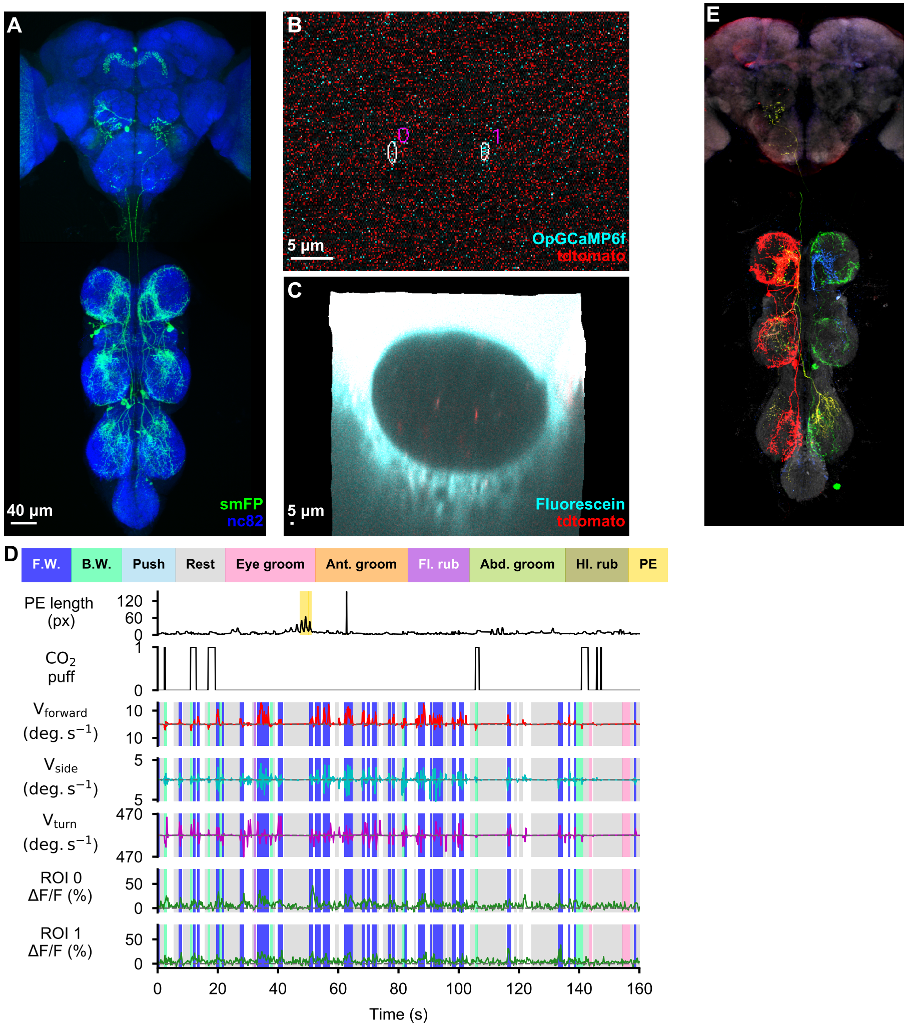

20190704-SS42749-tdTomGC6fopt-fly1-002

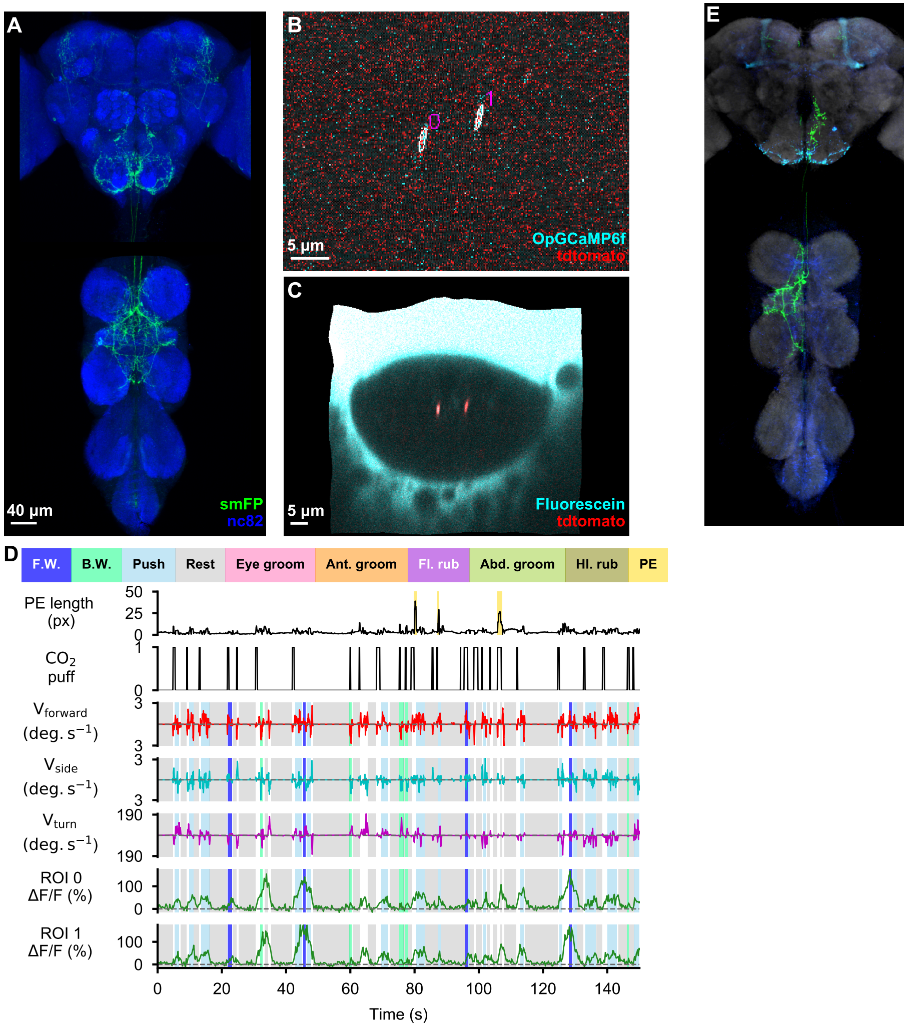

20190629-SS41815-tdTomGC6fopt-fly1-002

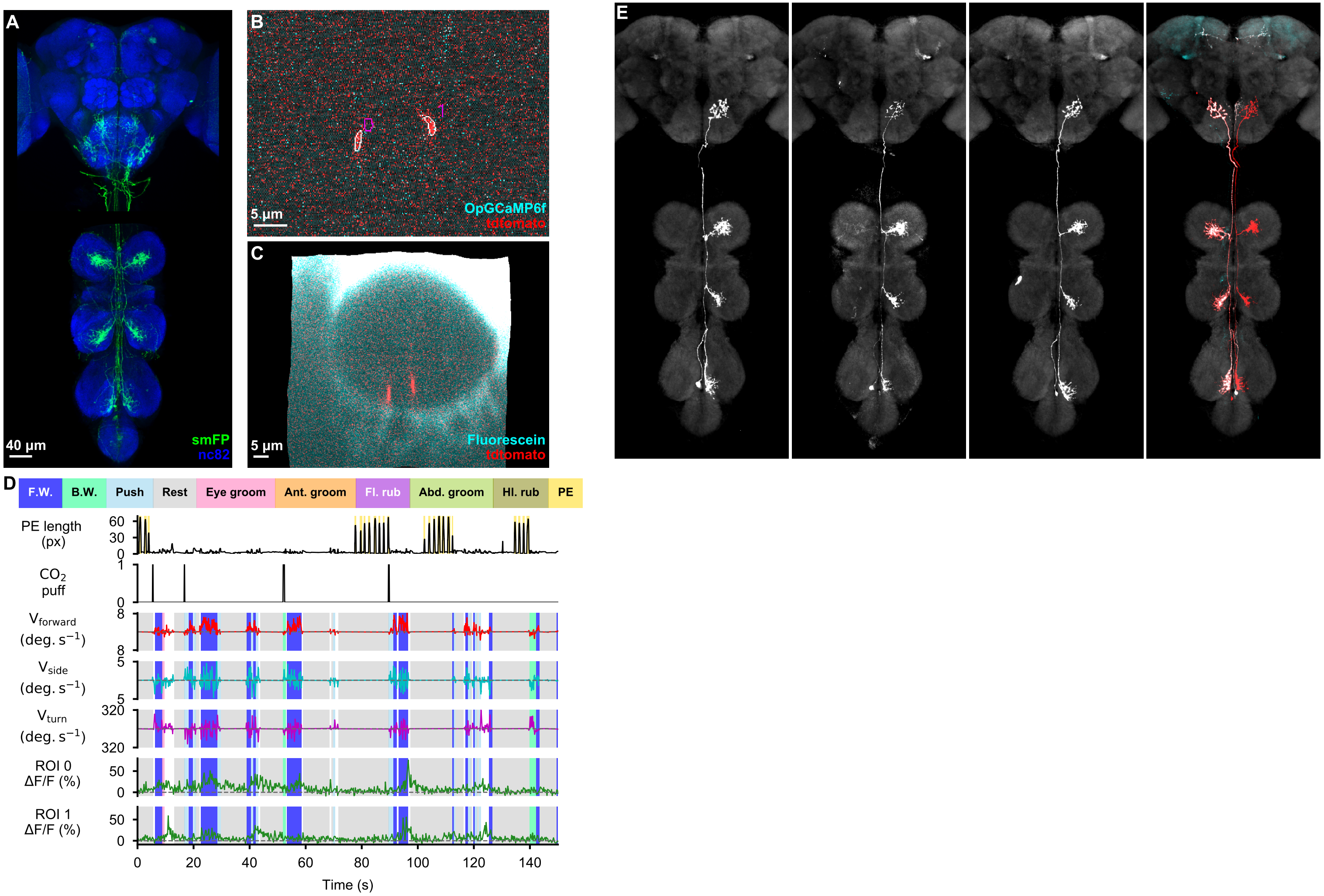

20190415-SS29633-tdTomGC6fopt-fly1-005

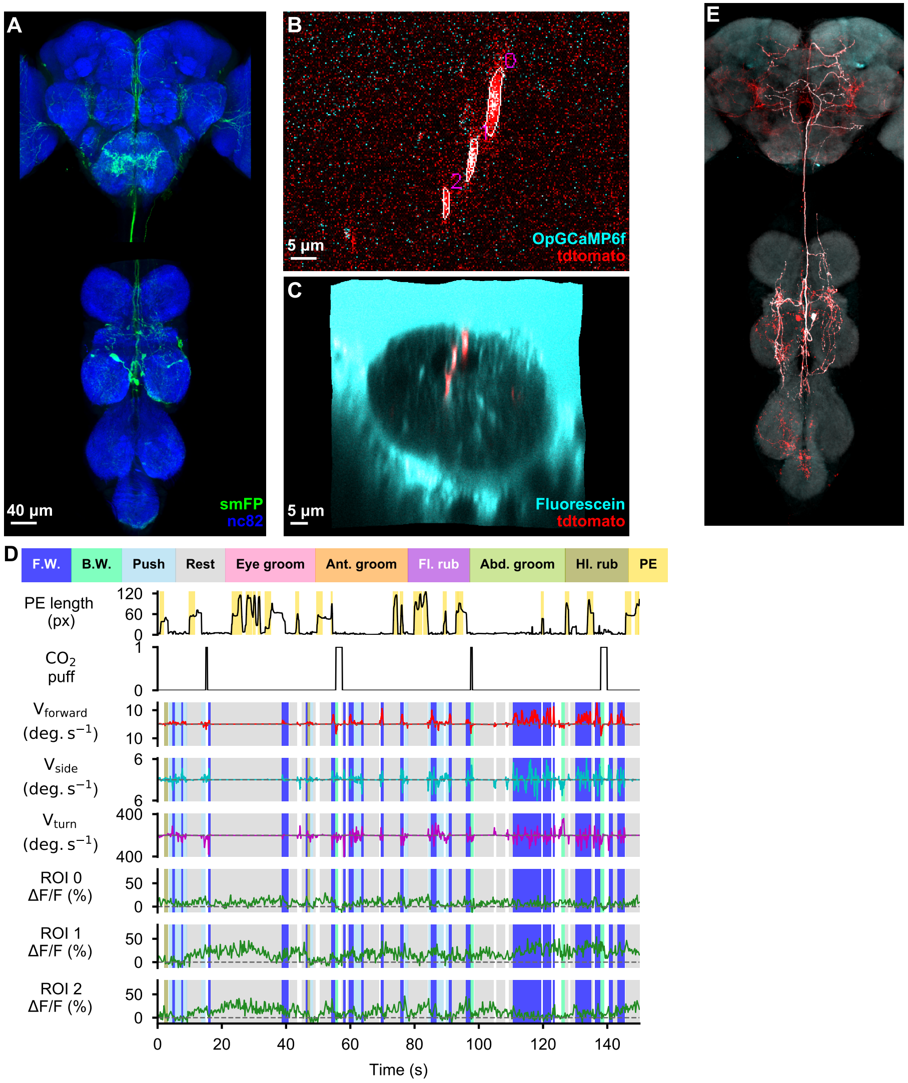

20181227-R87H02-tdTomGC6fopt-fly3-004

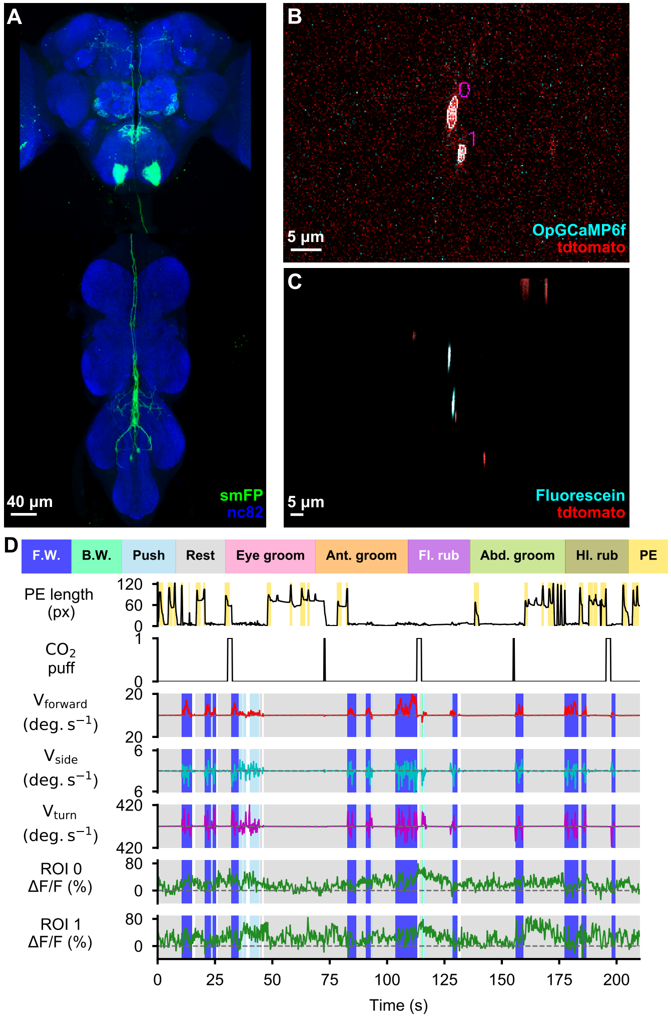

E

MCFO experiment was not performed.

20180720-MAN-tdTomGC6fopt-fly2-005

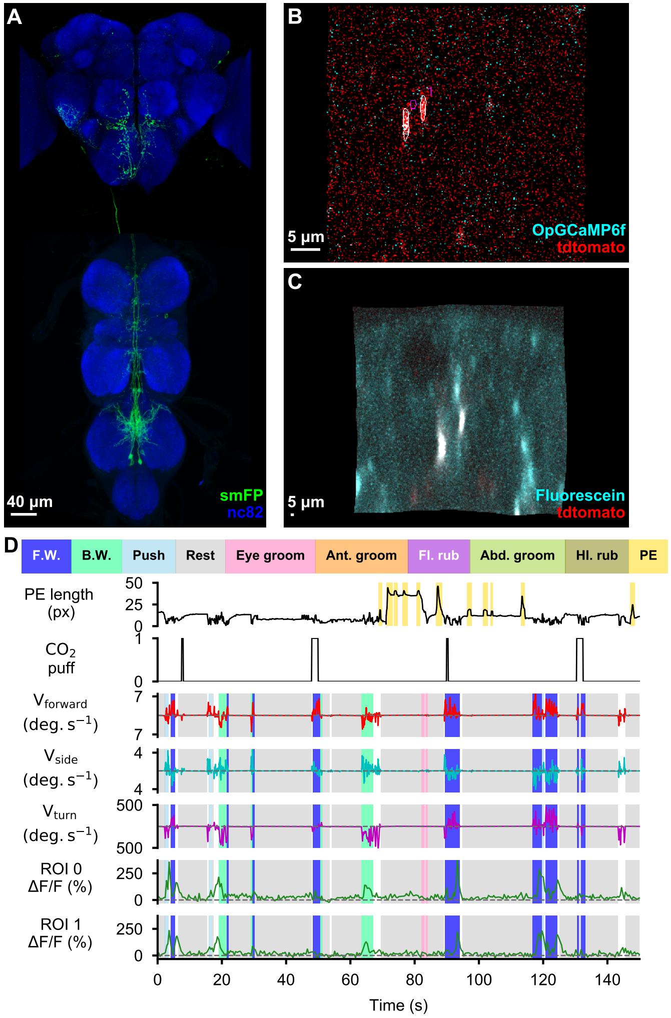

E

MCFO experiment was not performed.

20191001-SS49172-tdTomGC6fopt-fly1-001

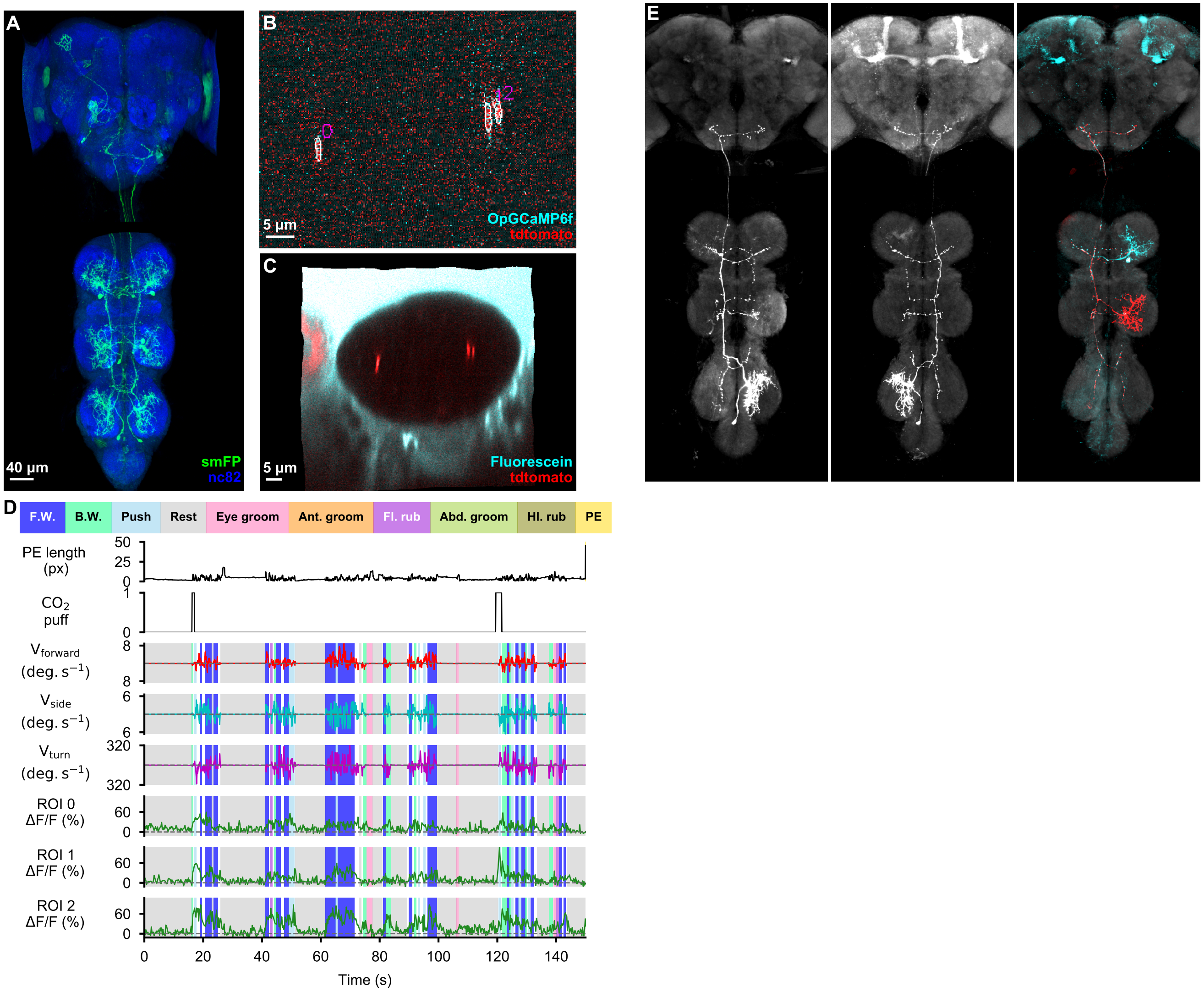

20181230-R36G04-tdTomGC6fopt-fly1-003

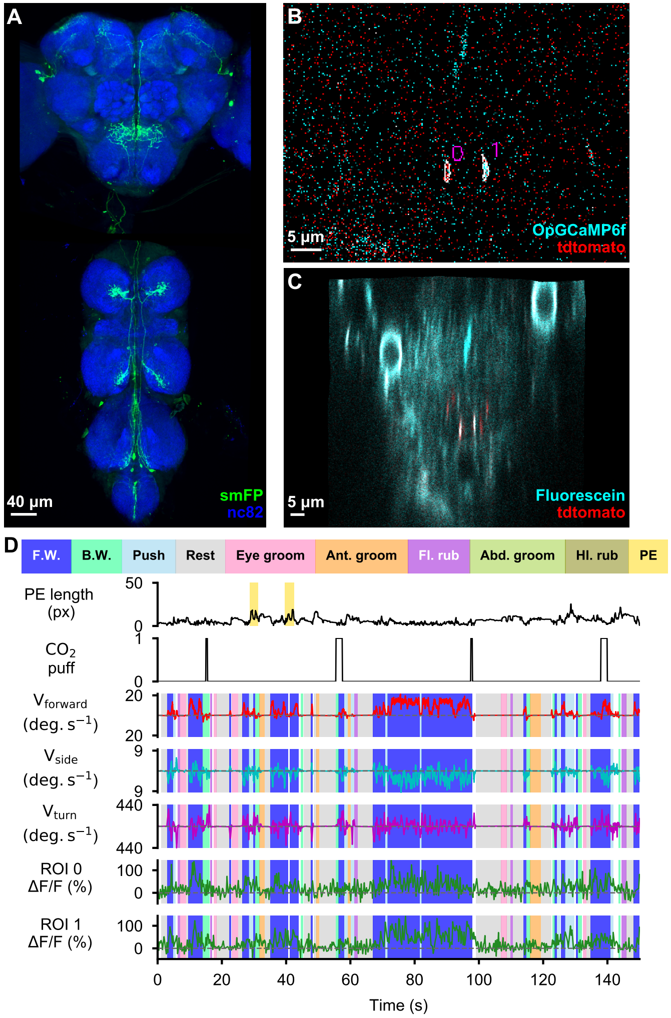

E

MCFO experiment was not performed.

20190105-R39G01-tdTomGC6fopt-fly1-003

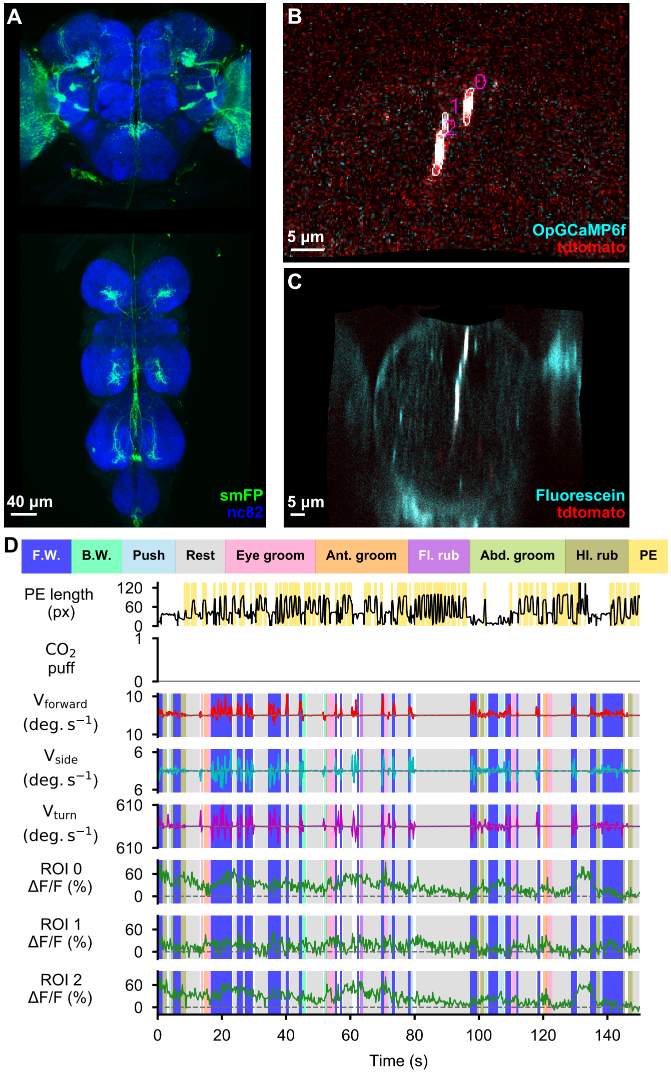

E

MCFO experiment was not performed.

20190408-SS31219-tdTomGC6fopt-fly1-002

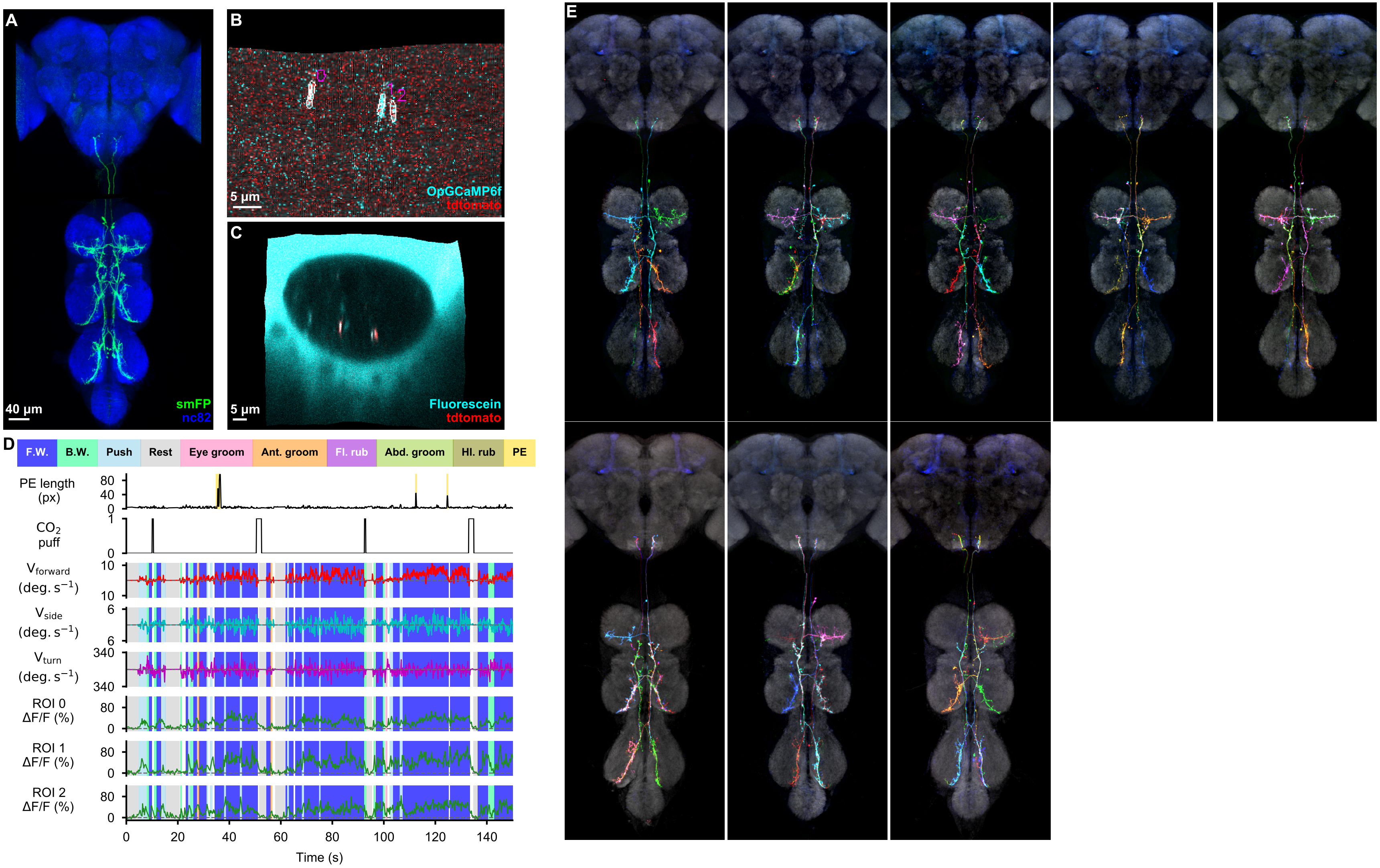

20181128-R30A08-tdTomGC6fopt-fly2-001

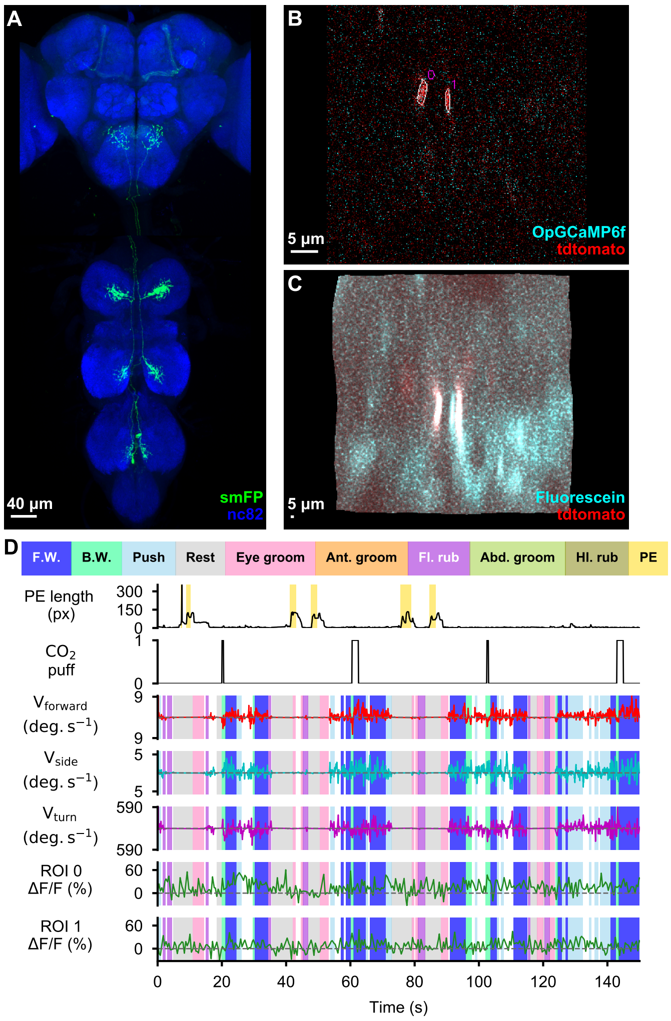

E

MCFO experiment was not performed.

20190717-SS44270-tdTomGC6fopt-fly1-004

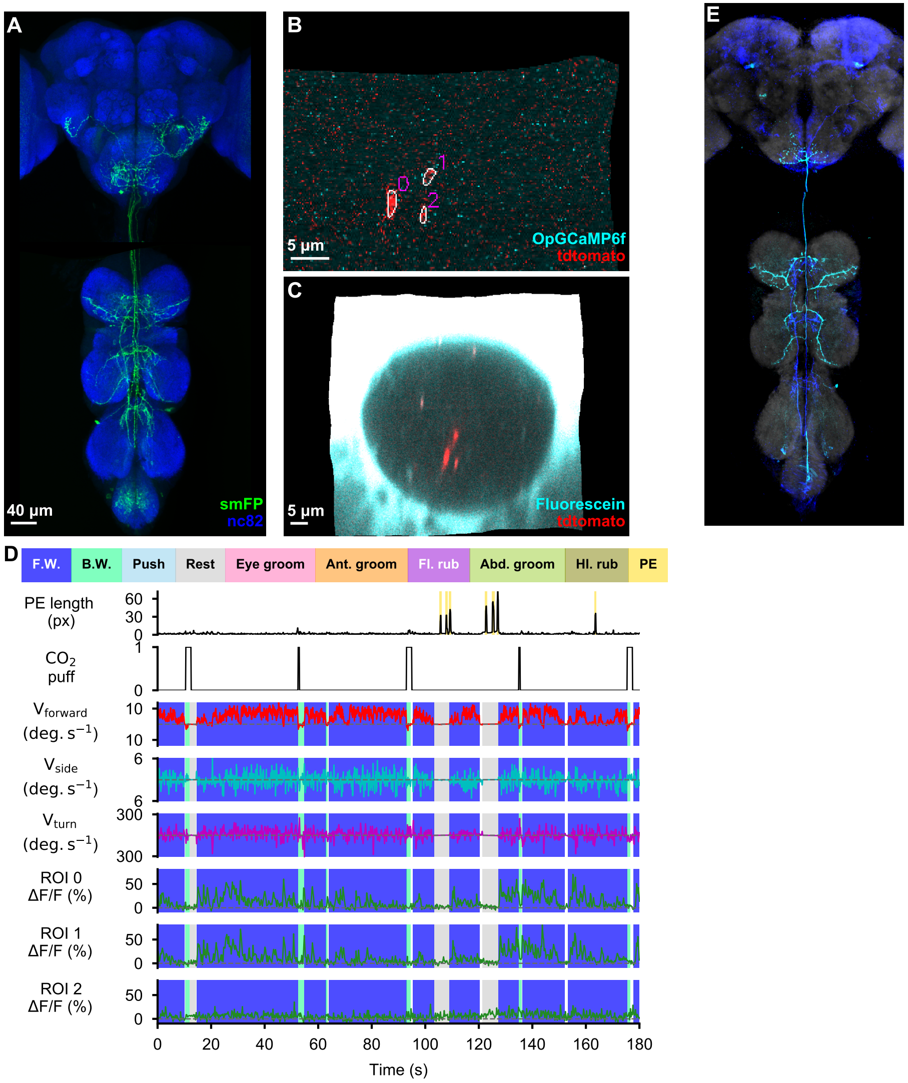

20190619-SS41605-tdTomGC6fopt-fly3-003

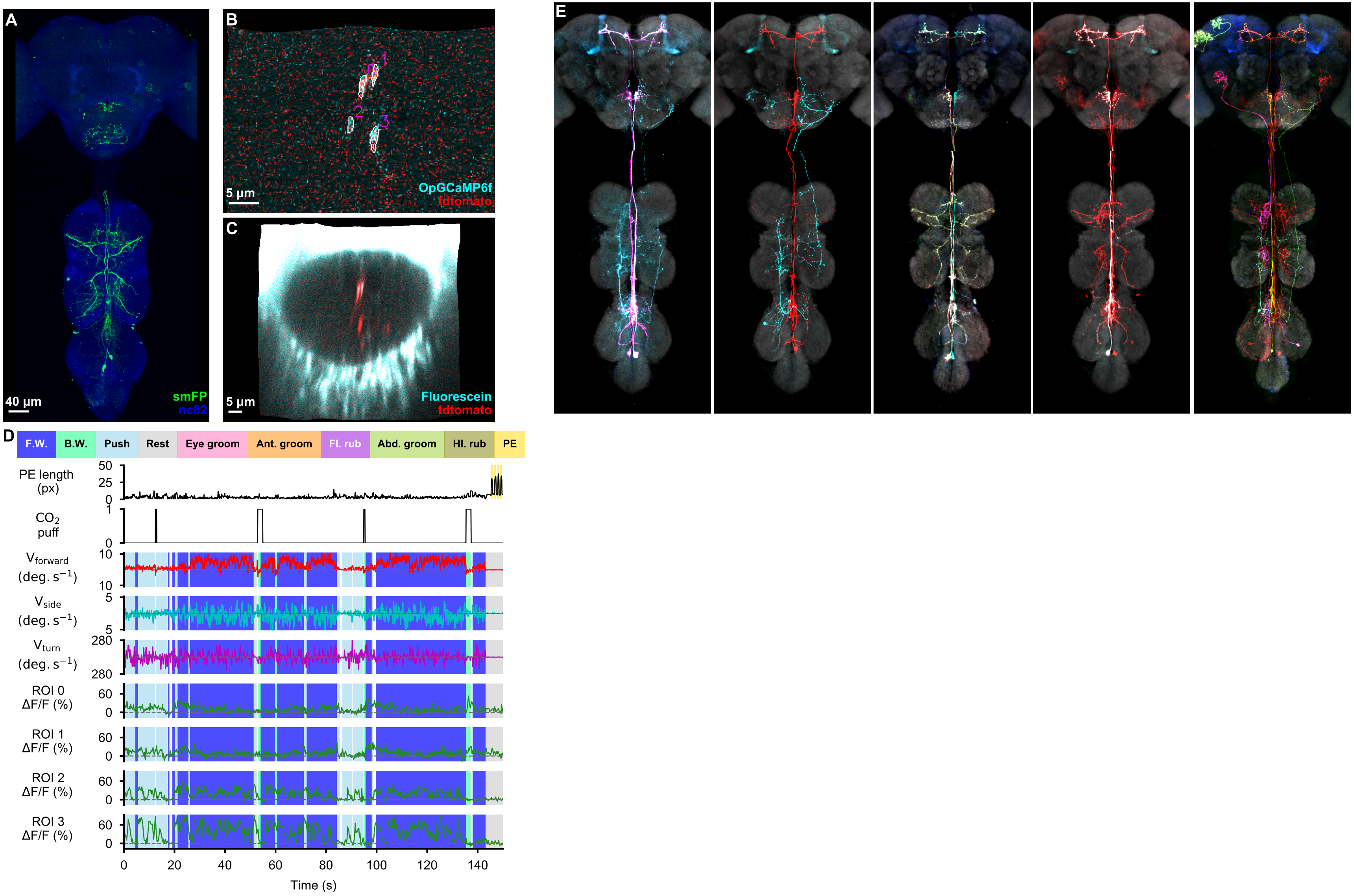

20190221-SS29579-tdTomGC6fopt-fly1-003

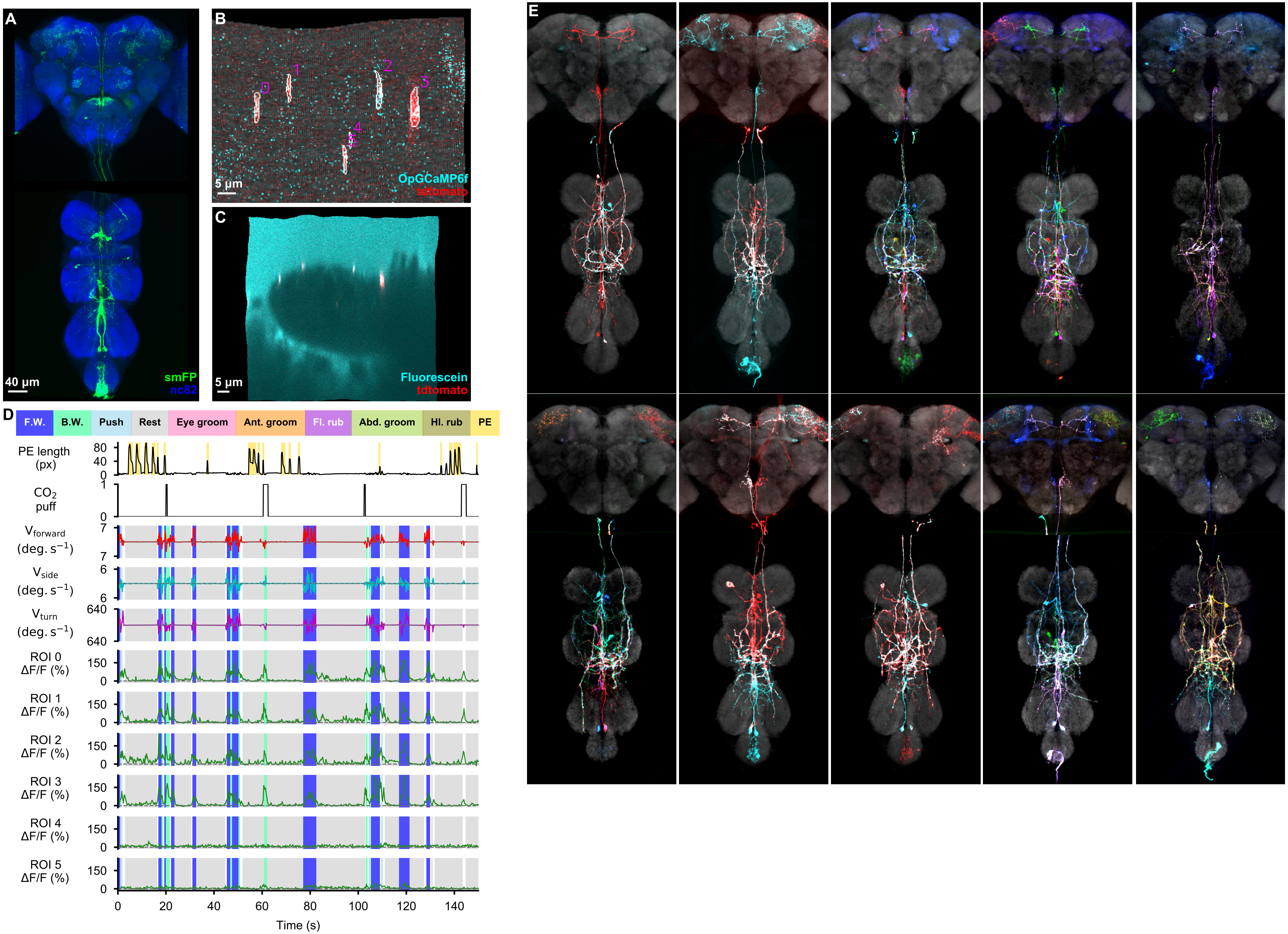

20191002-SS51046-tdTomGC6fopt-fly1-004

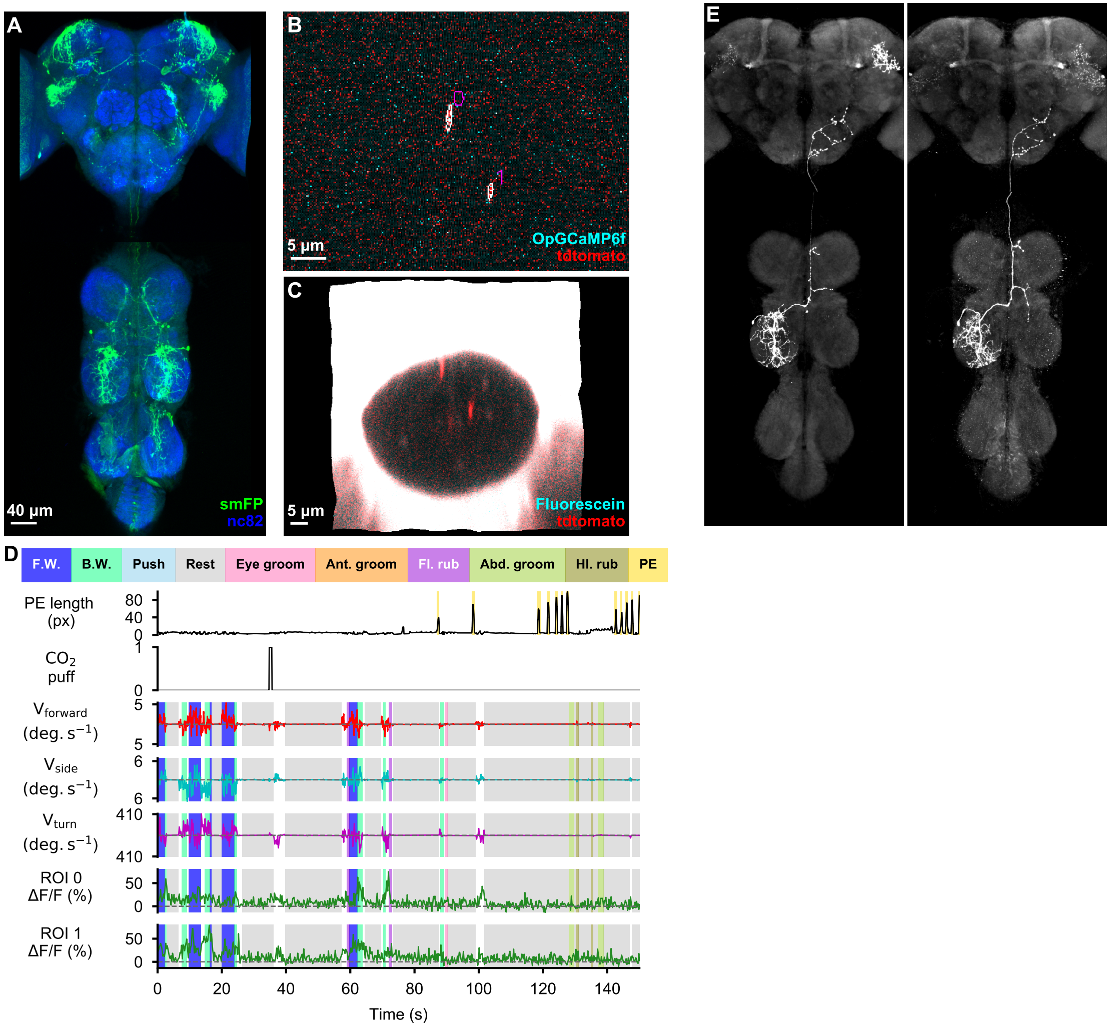

20190328-SS29893-tdTomGC6fopt-fly3-001

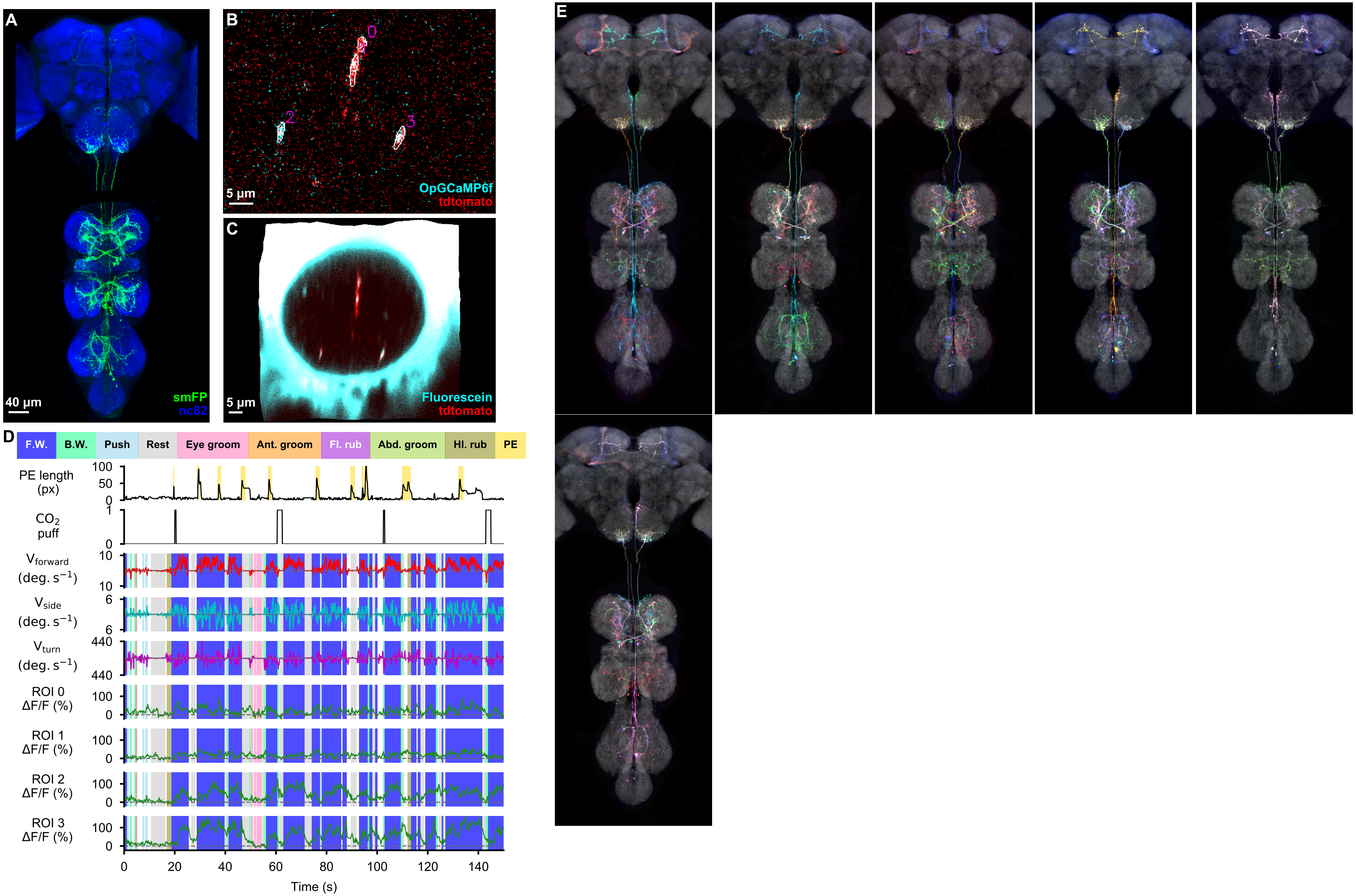

20190522-SS34574-tdTomGC6fopt-fly2-001

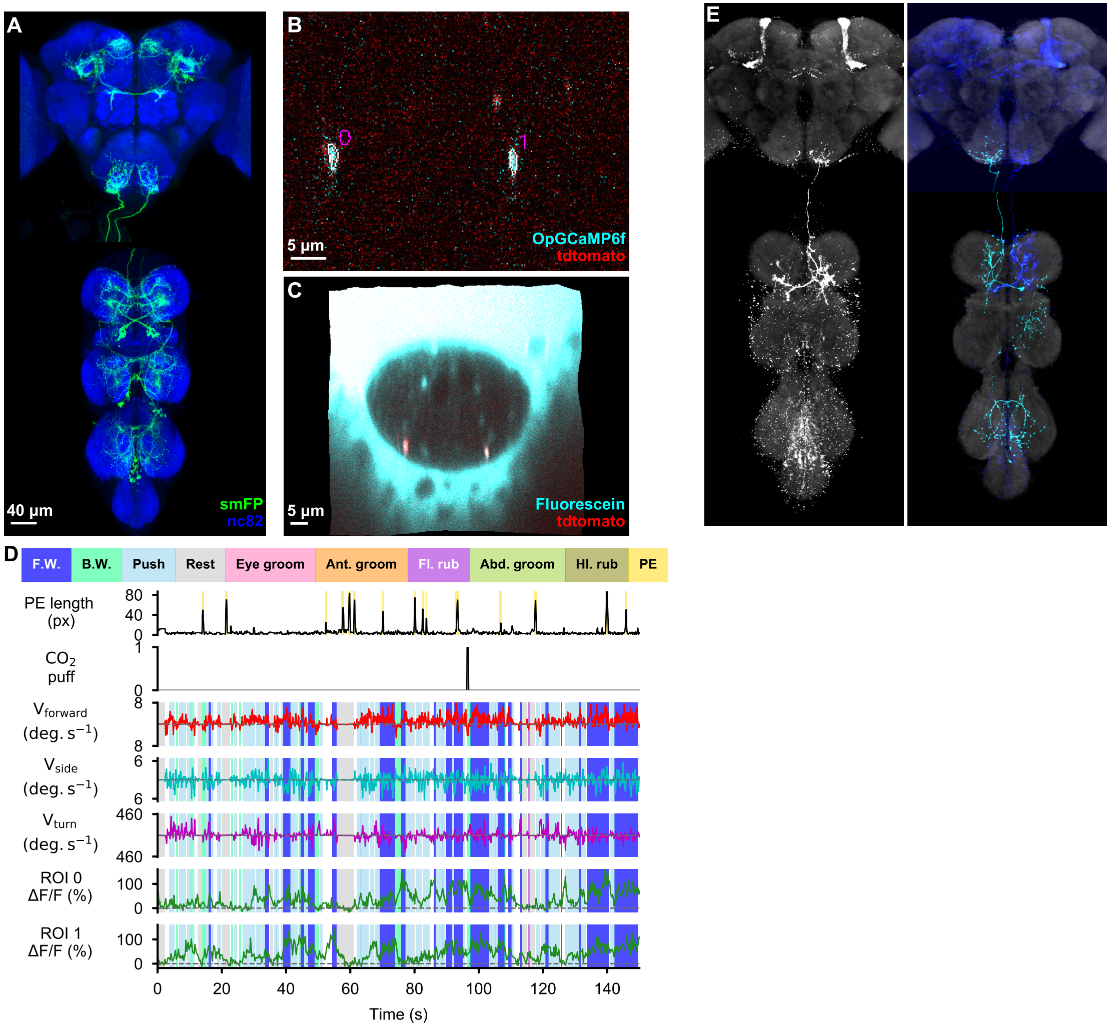

20181202-R70H06-tdTomGC6fopt-fly1-002

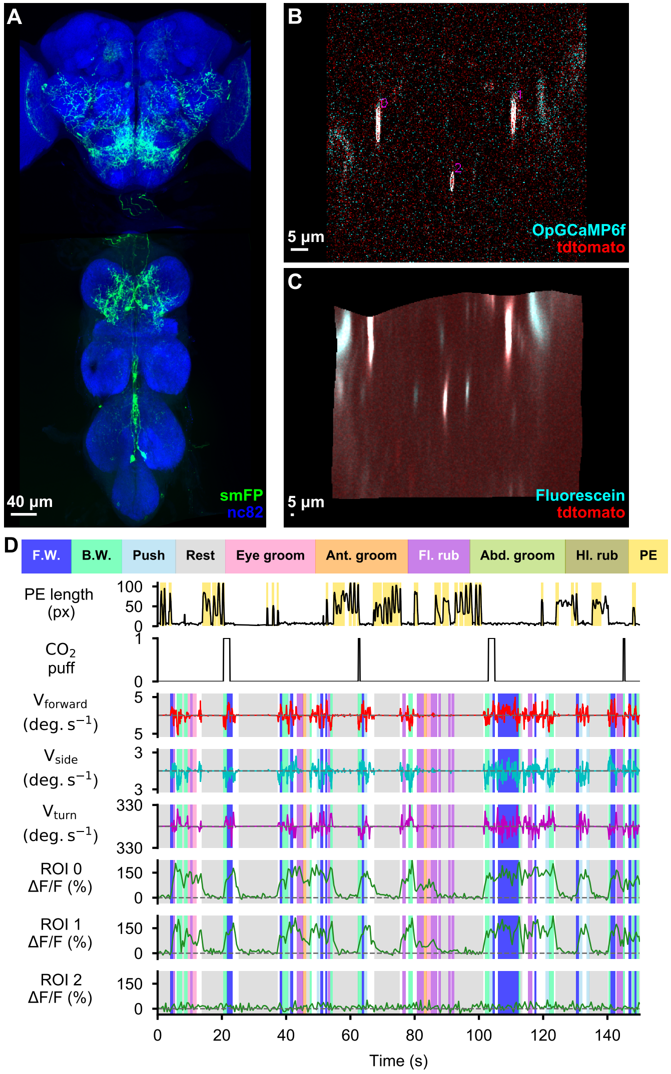

E

MCFO experiment was not performed.

20190703-SS42740-tdTomGC6fopt-fly2-011

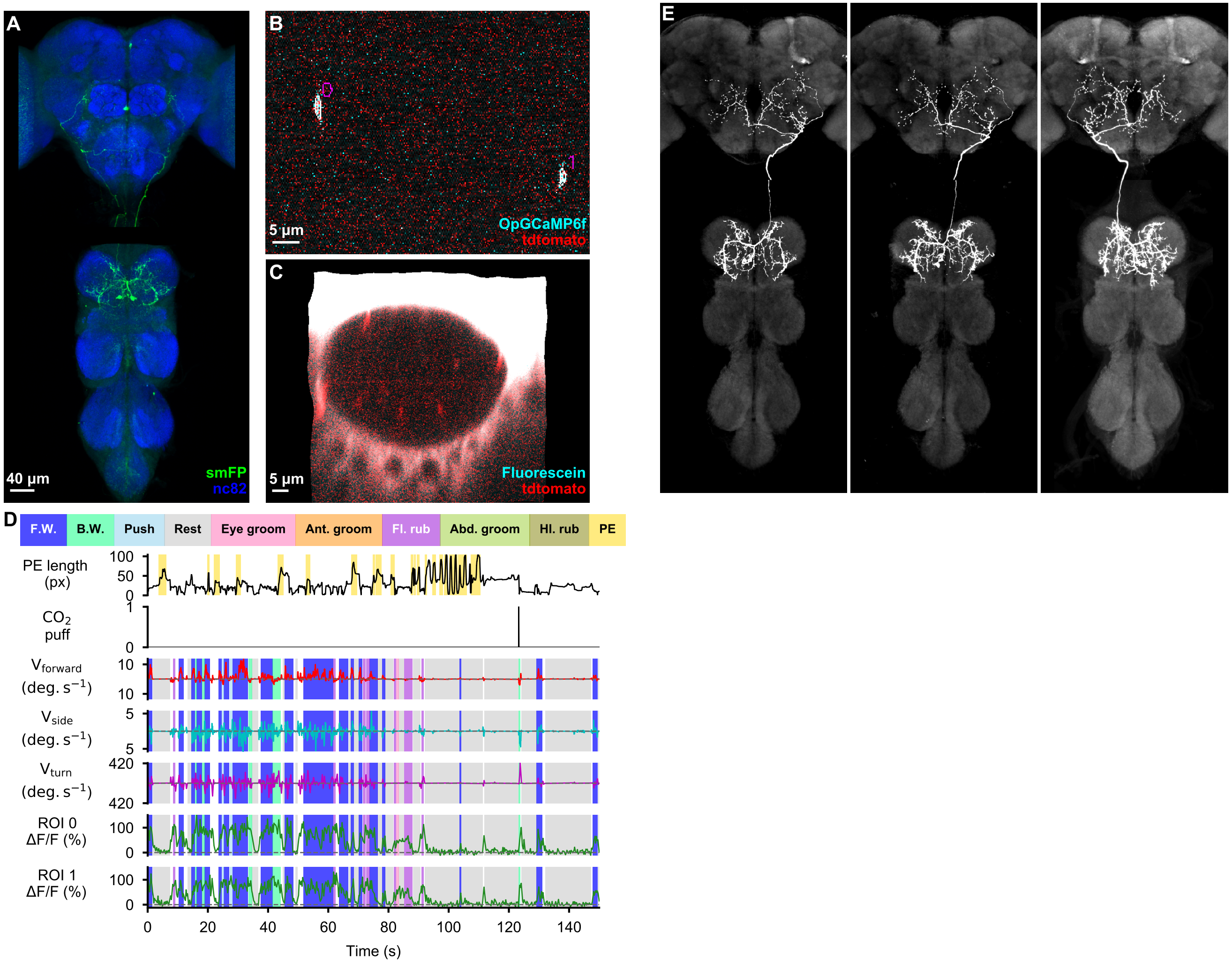

20190326-SS28596-tdTomGC6fopt-fly2-008

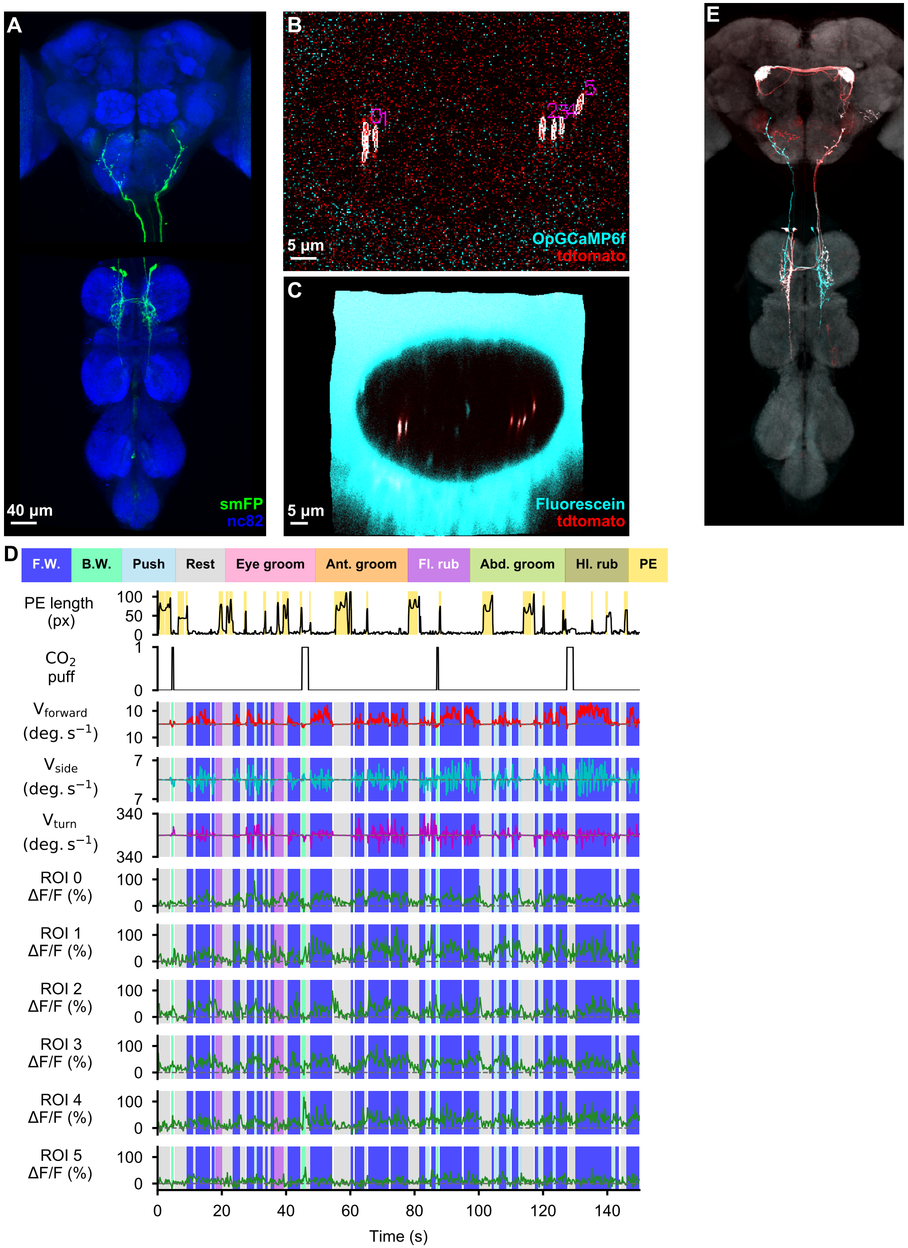

20190220-SS25469-tdTomGC6fopt-fly1-006

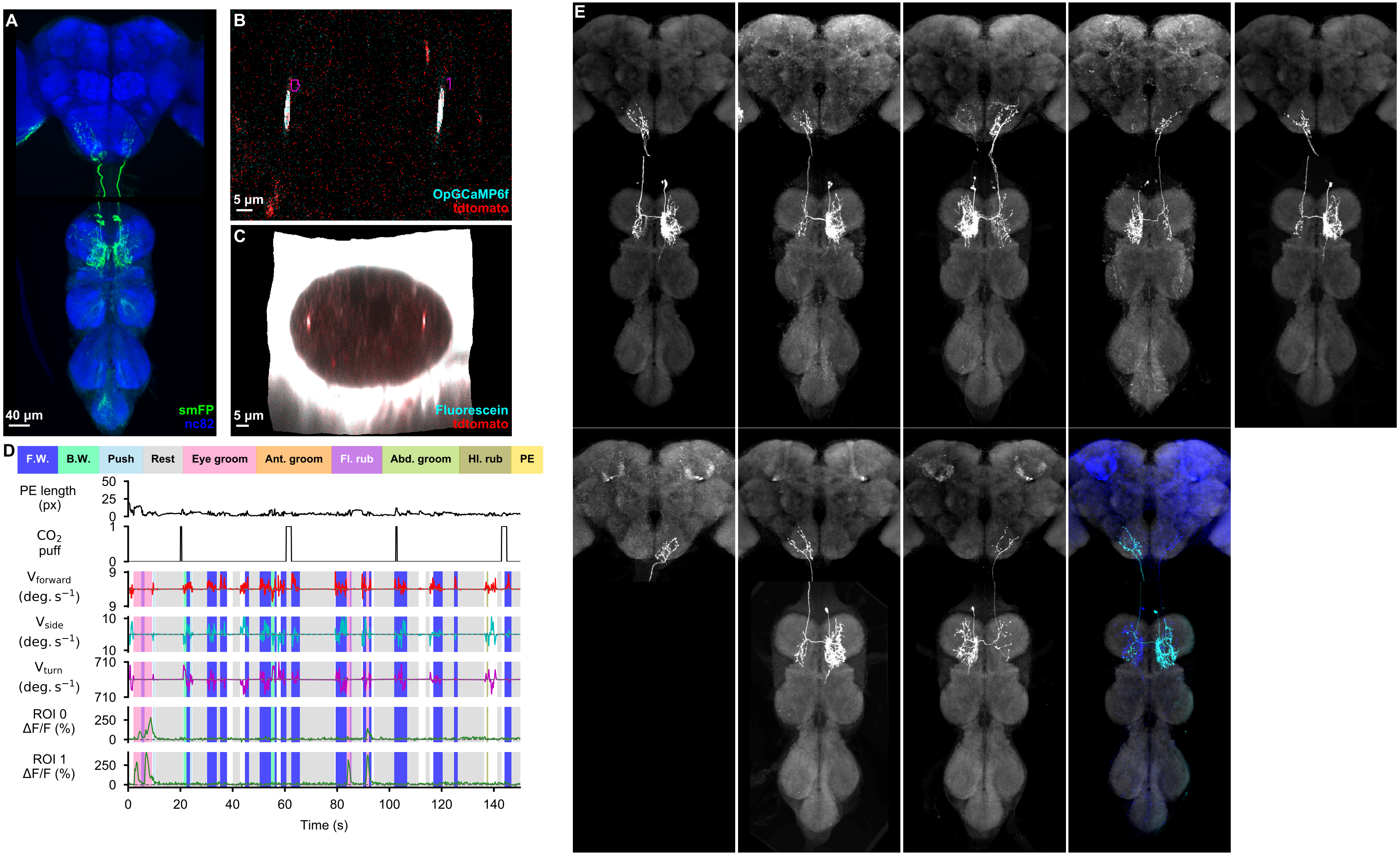

20190412-SS31232-tdTomGC6fopt-fly3-001

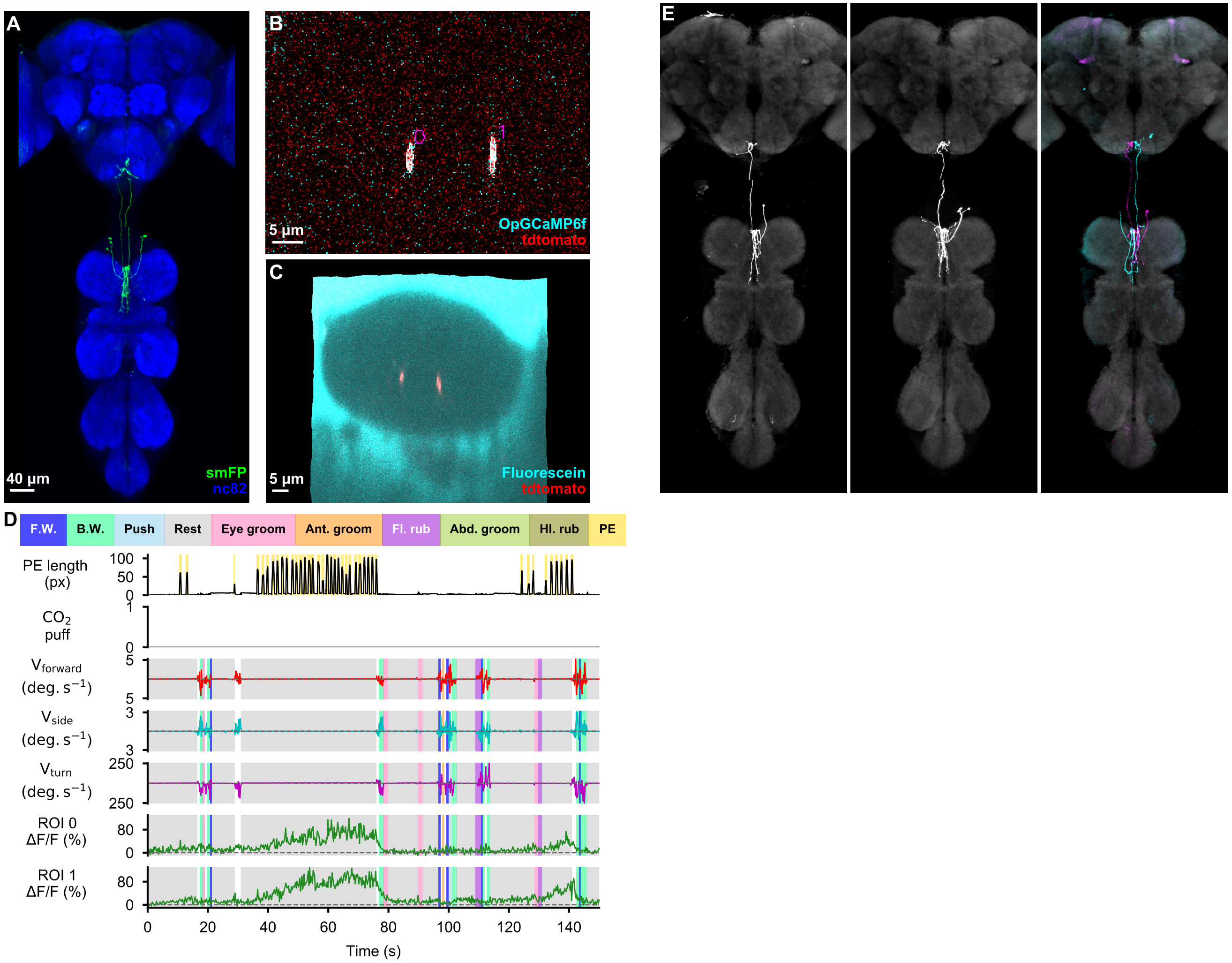

20190405-SS30303-tdTomGC6fopt-fly2-005

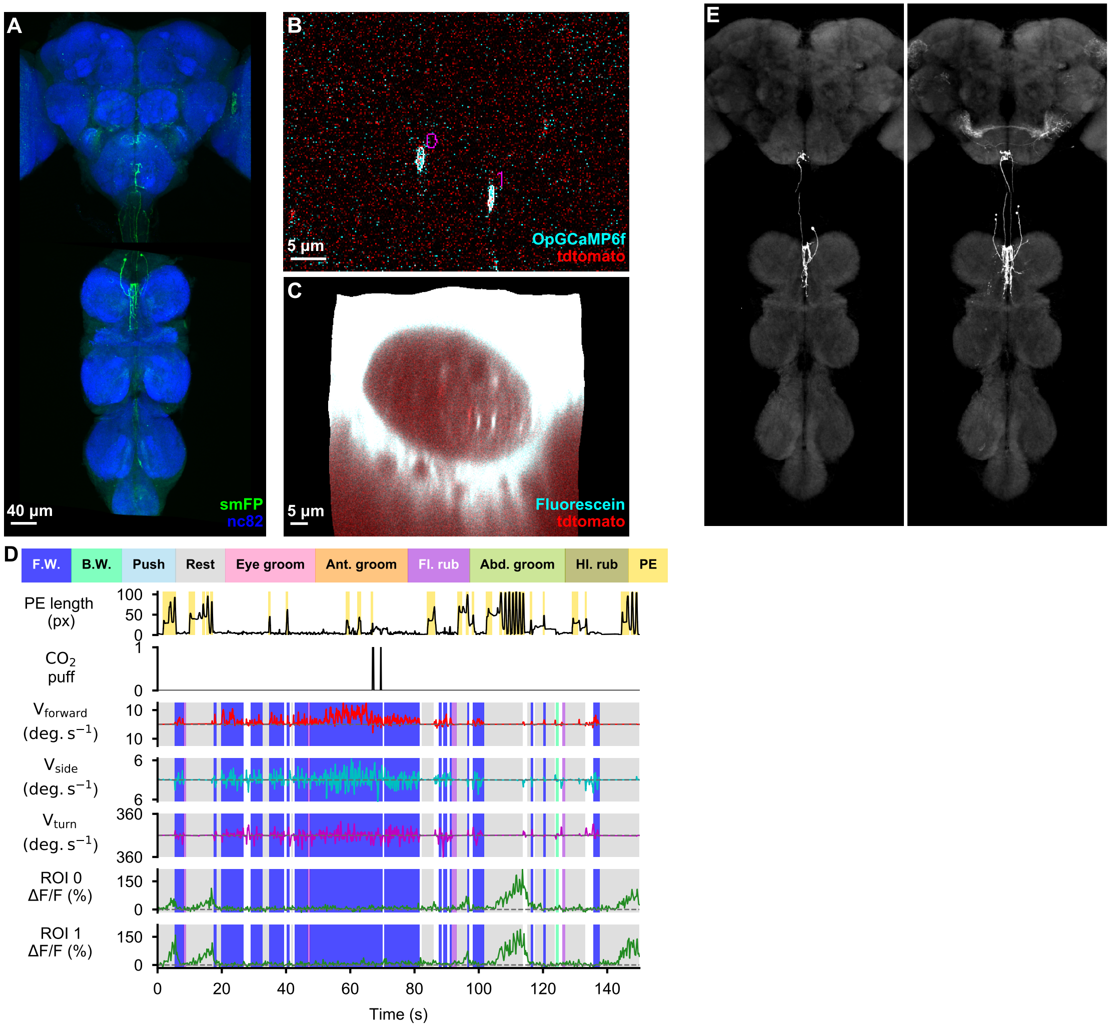

20180822-SS25451-tdTomGC6fopt-fly4-012

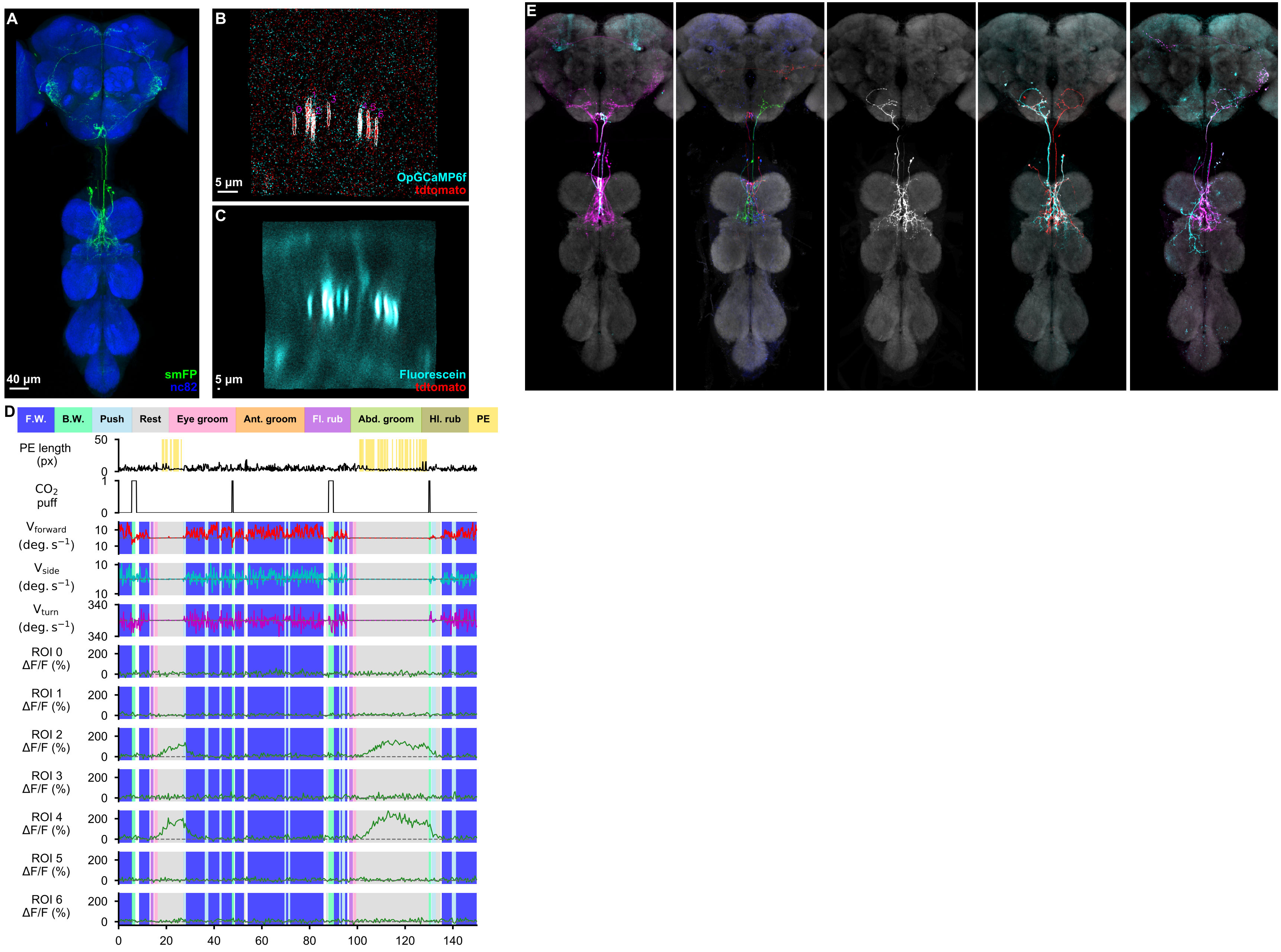

20190615-SS40134-tdTomGC6fopt-fly1-001

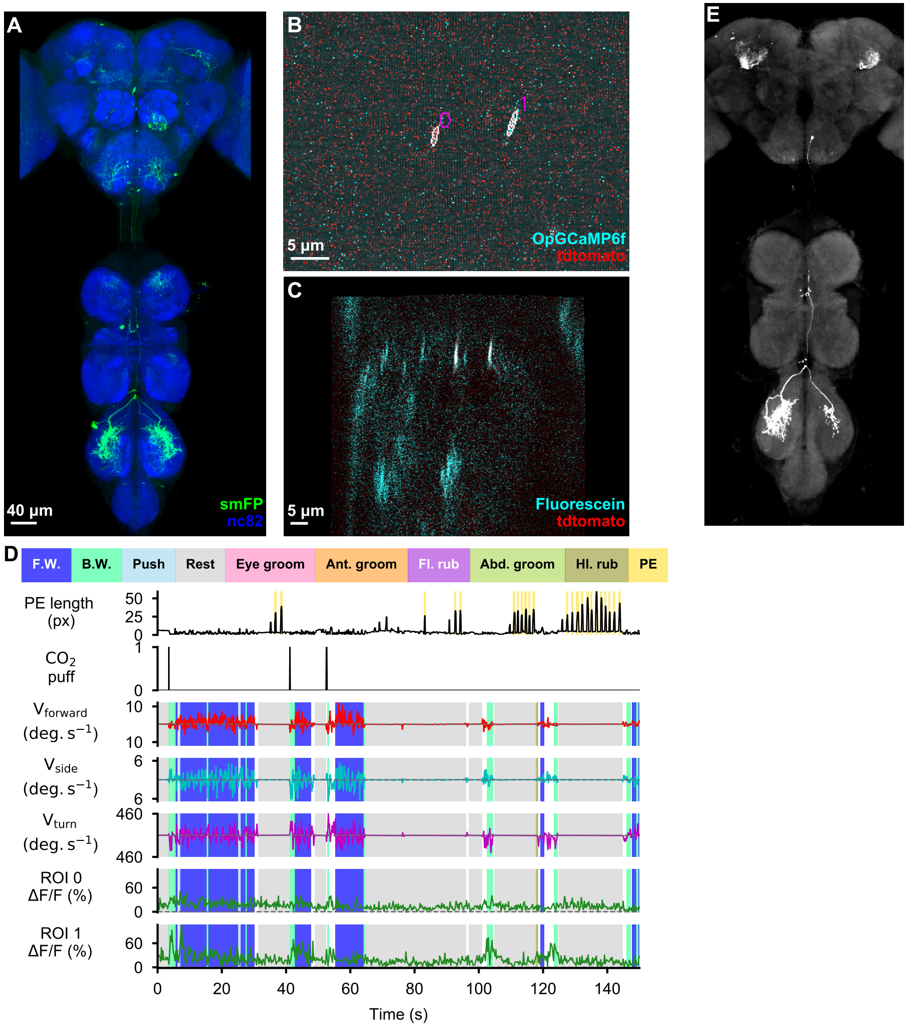

20190318-SS29621-tdTomGC6fopt-fly1-006

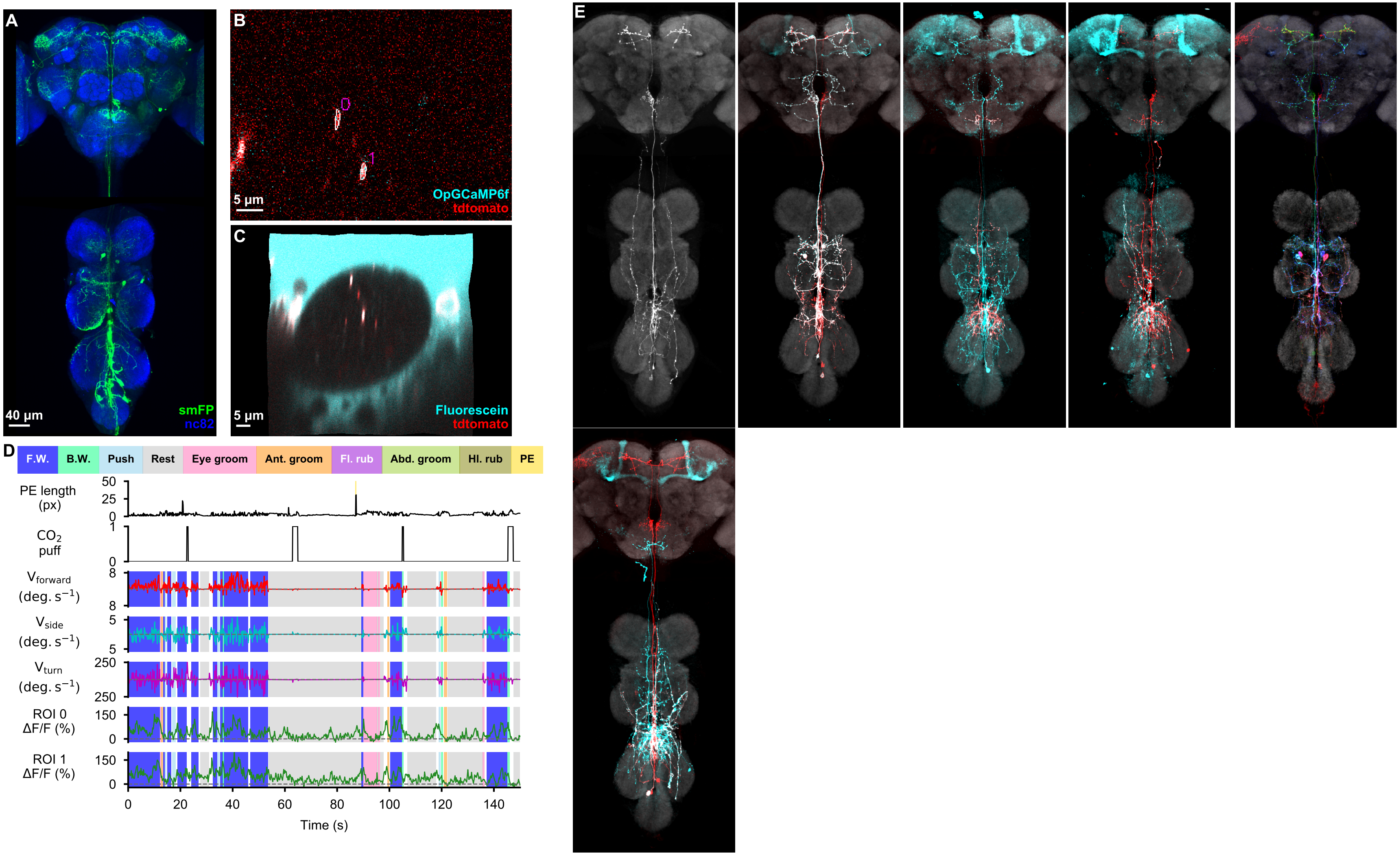

20190118-R69H10-tdTomGC6fopt-fly2-001

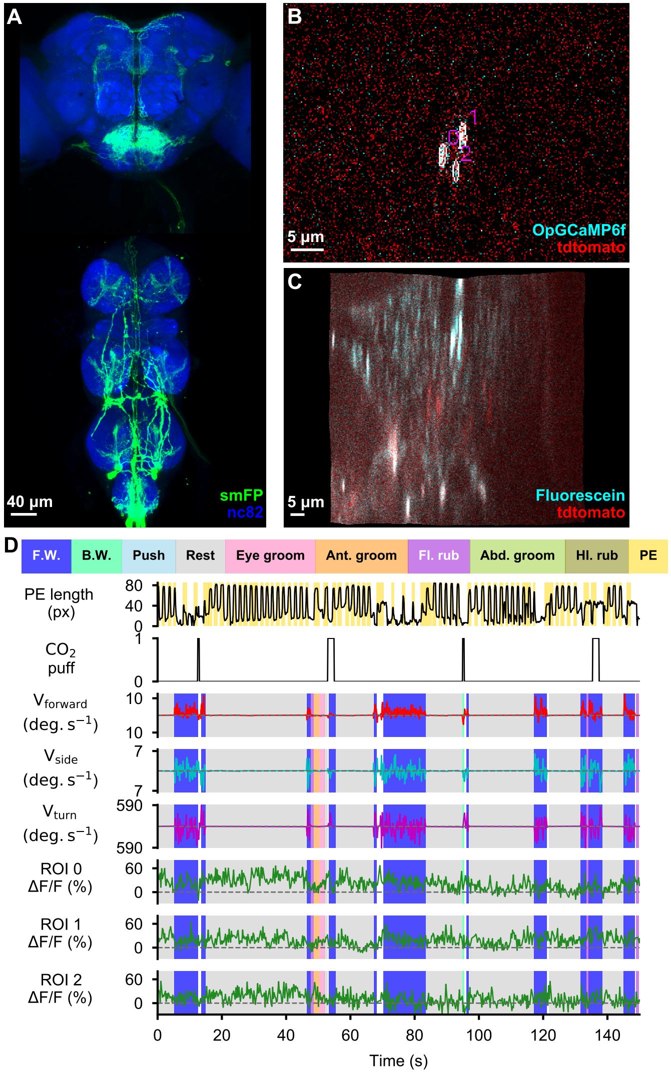

E

MCFO experiment was not performed.

20190910-SS51038-tdTomGC6fopt-fly1-005

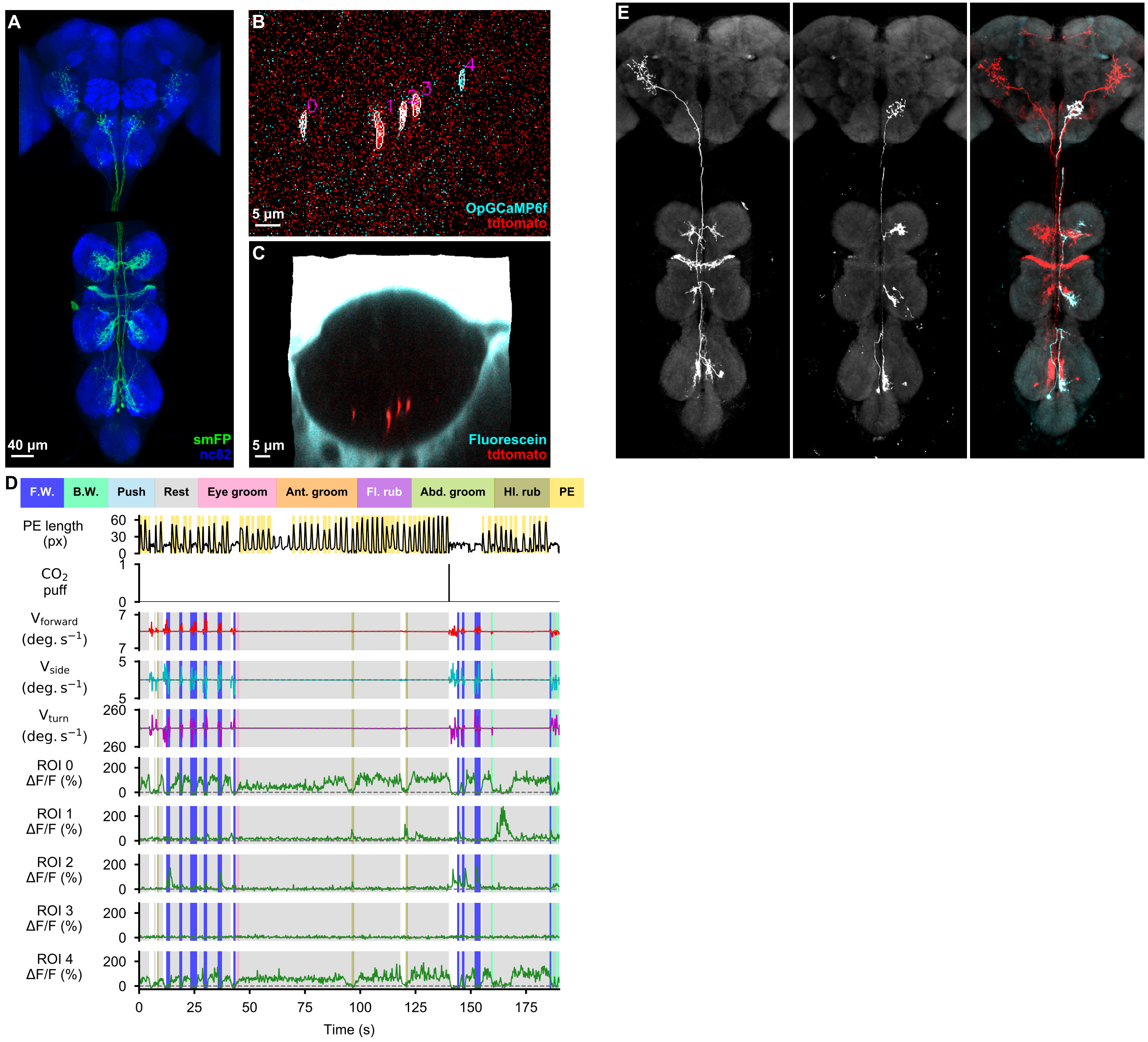

20190701-SS42008-tdTomGC6fopt-fly4-003

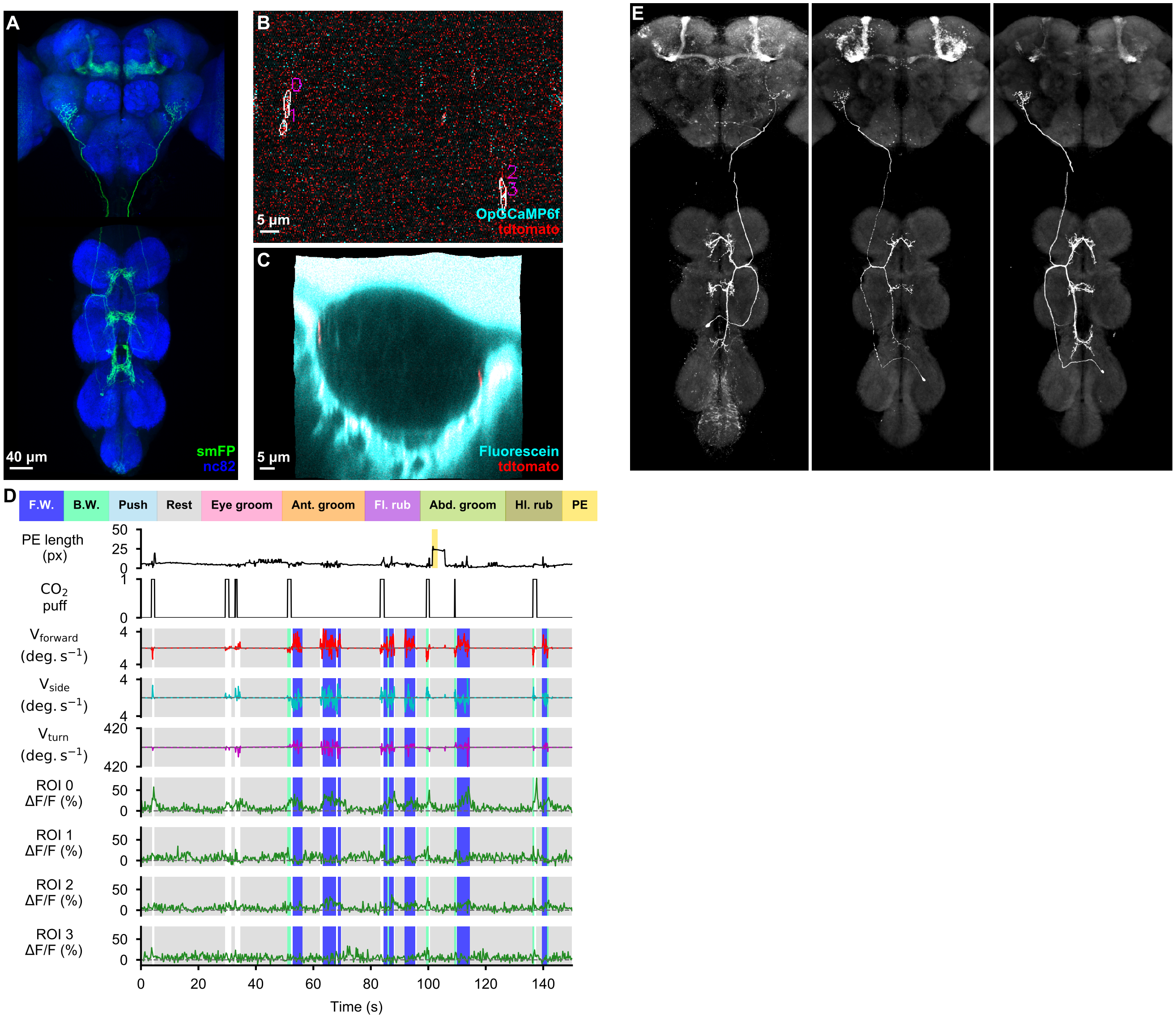

20190516-SS36118-tdTomGC6fopt-fly4-003

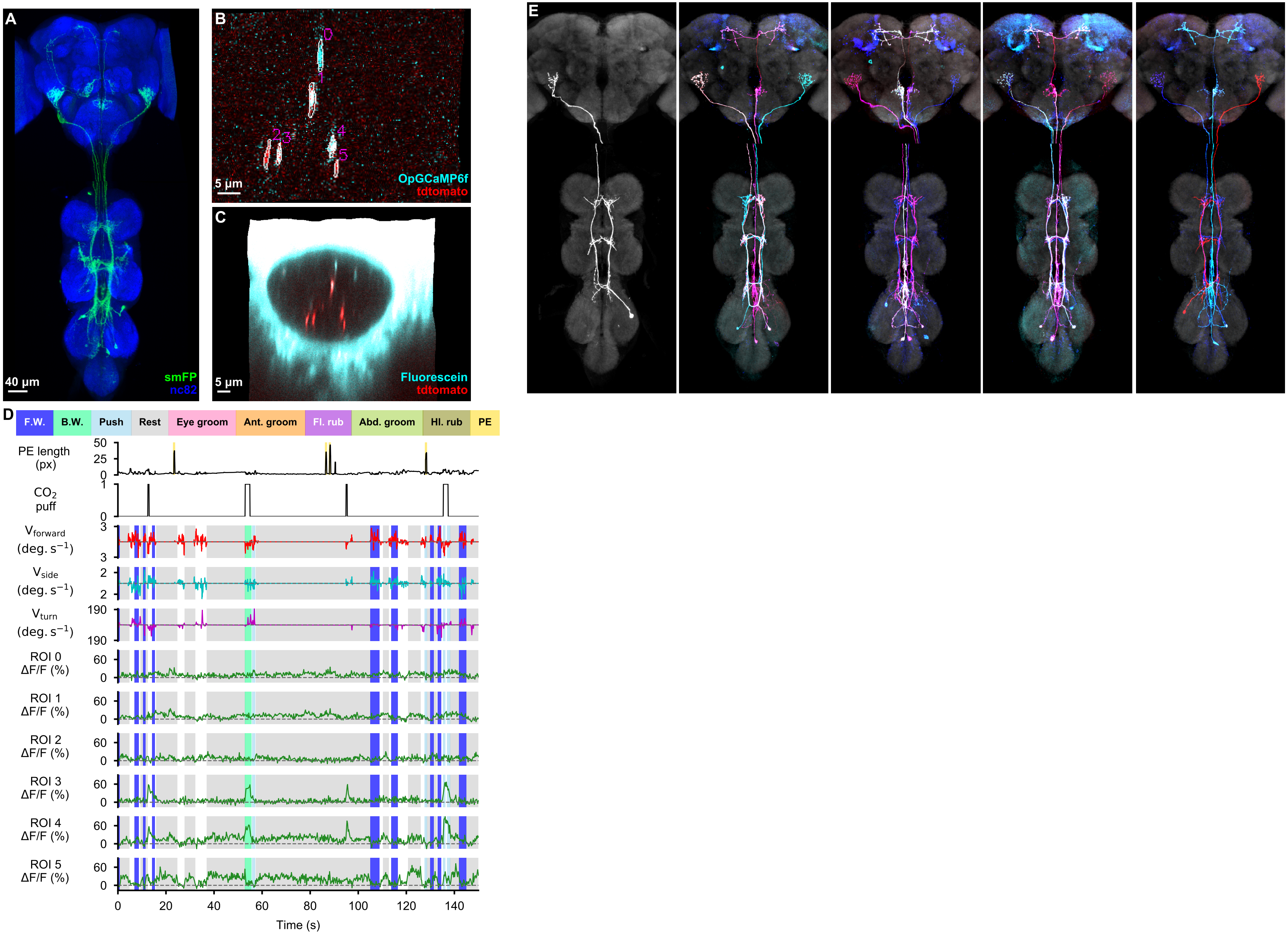

20190621-SS40619-tdTomGC6fopt-fly1-002

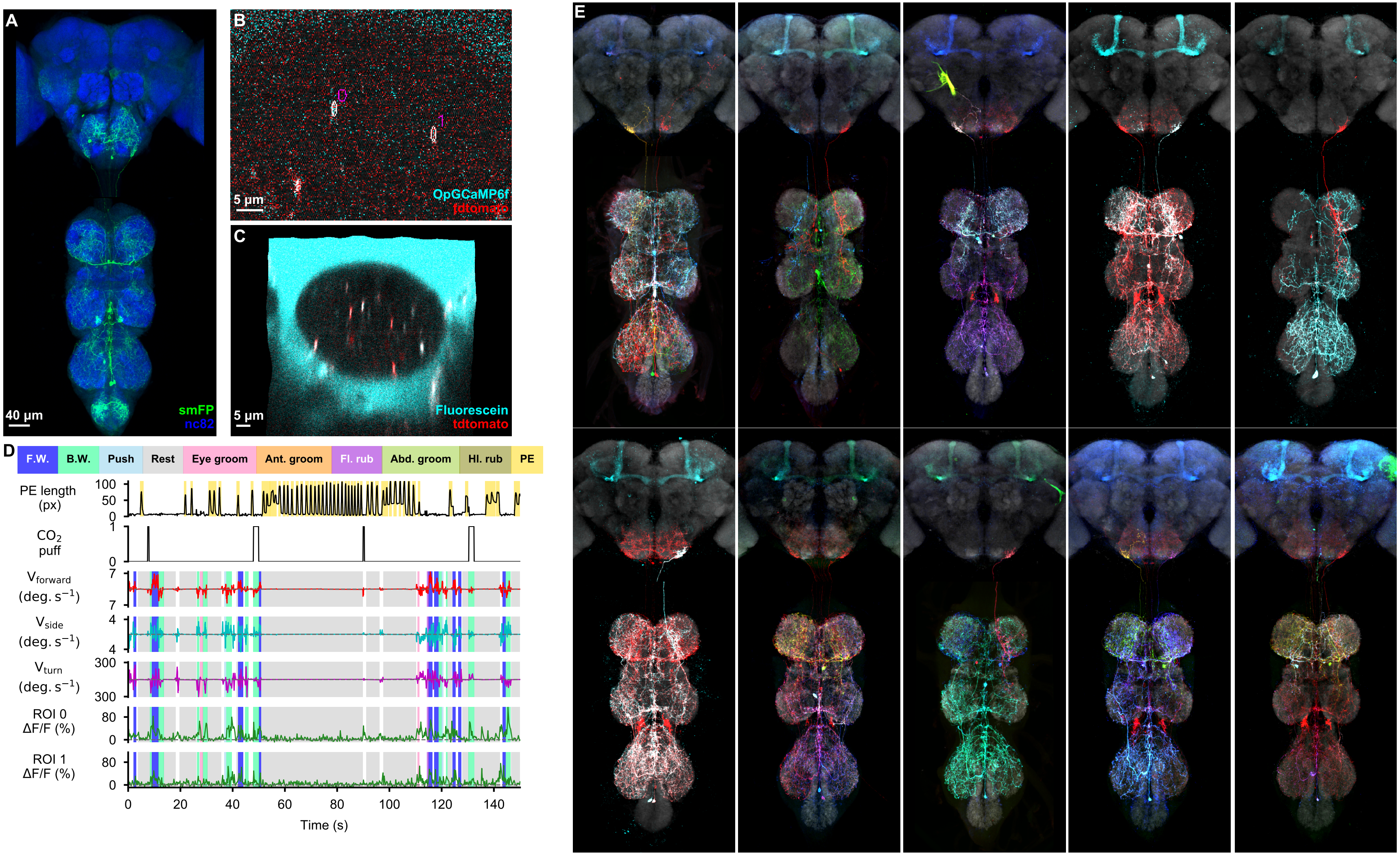

20190723-SS45363-tdTomGC6fopt-fly1-013

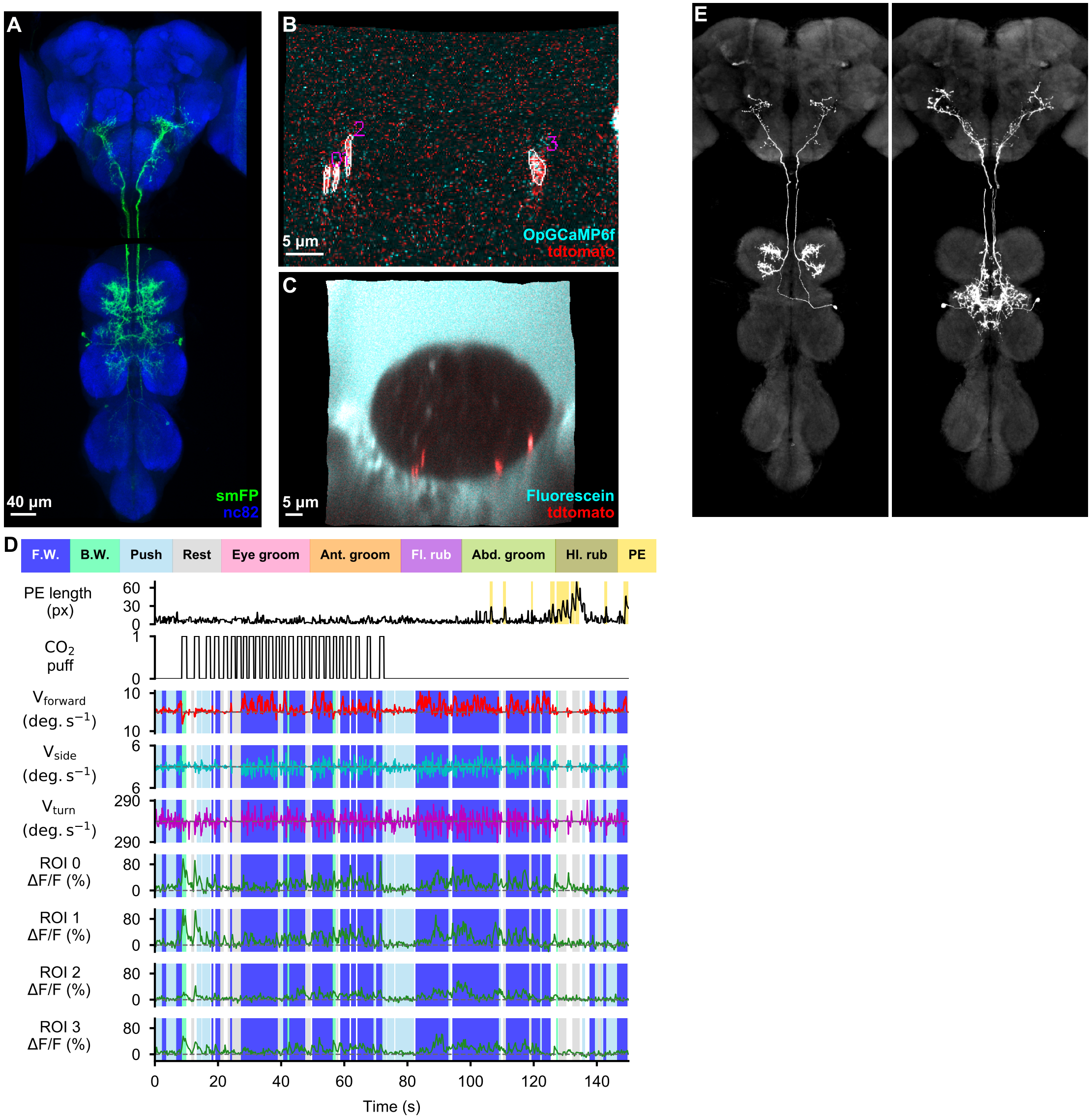

20190918-SS52147-tdTomGC6fopt-fly1-003

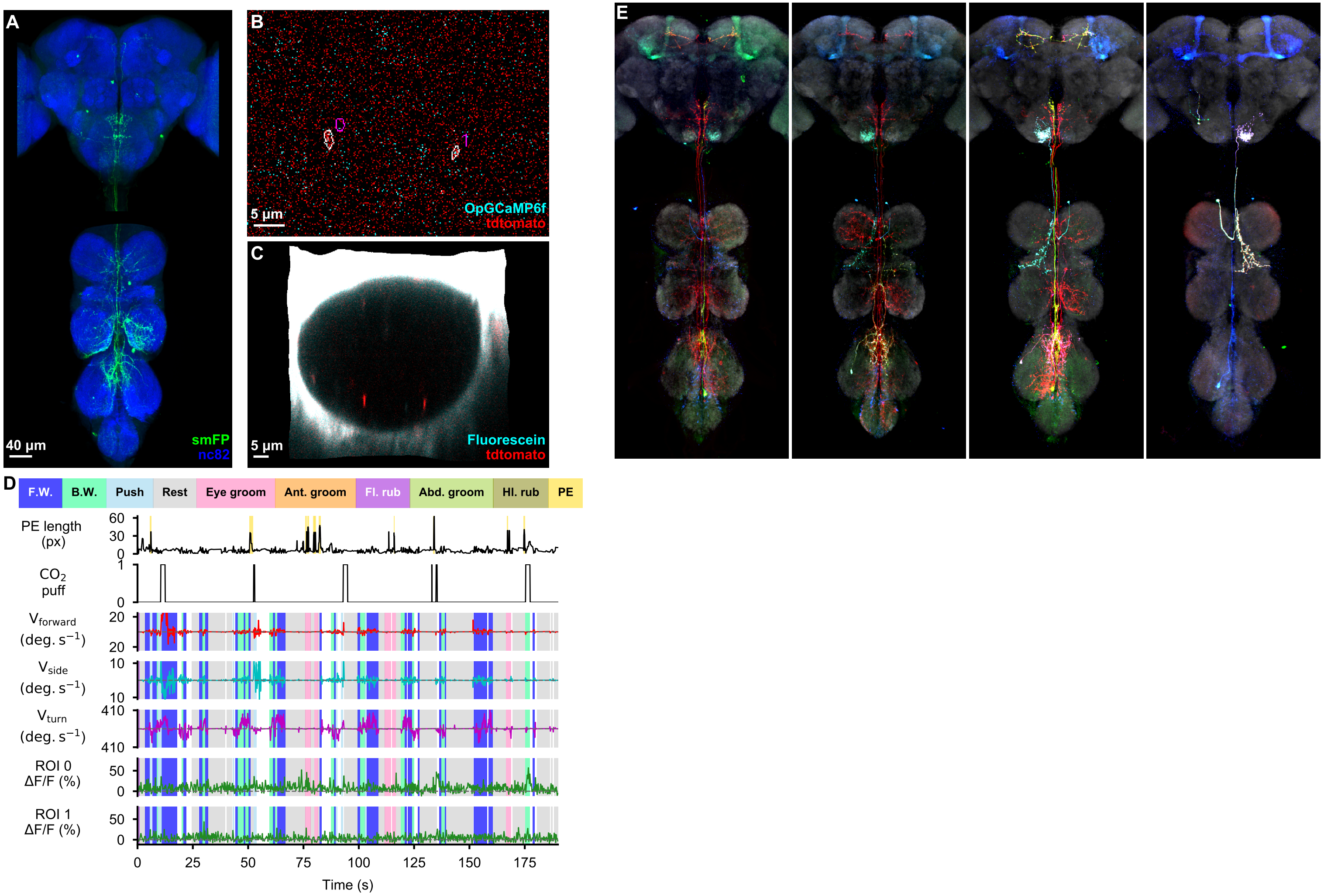

Supplement: Supplementary file 5 — Supplementary Data. Data for each examined driver line. [file 41593_2023_1281_MOESM5_ESM.pdf]
